# Stable Catechol Keto Tautomers in Cytotoxic Heterodimeric Cyclic Diarylheptanoids from the Seagrass *Zostera marina*

**DOI:** 10.1021/acs.orglett.1c02537

**Published:** 2021-09-07

**Authors:** Yan Li, Laura Grauso, Silvia Scarpato, Nunzio Antonio Cacciola, Francesca Borrelli, Christian Zidorn, Alfonso Mangoni

**Affiliations:** † Pharmazeutisches Institut, Abteilung Pharmazeutische Biologie, Christian-Albrechts-Universität zu Kiel, Gutenbergstraße 76, 24118, Kiel, Germany; § Dipartimento di Agraria, Università degli Studi di Napoli Federico II, Via Università 100, 80055 Portici (NA), Italy; ⊥ Dipartimento di Farmacia, Università degli Studi di Napoli Federico II, Via D. Montesano 49, 80131 Napoli, Italy; # Dipartimento di Medicina Veterinaria e Produzioni Animali, Università degli Studi di Napoli Federico II, Via Via F. Delpino, 80137 Napoli, Italy

## Abstract

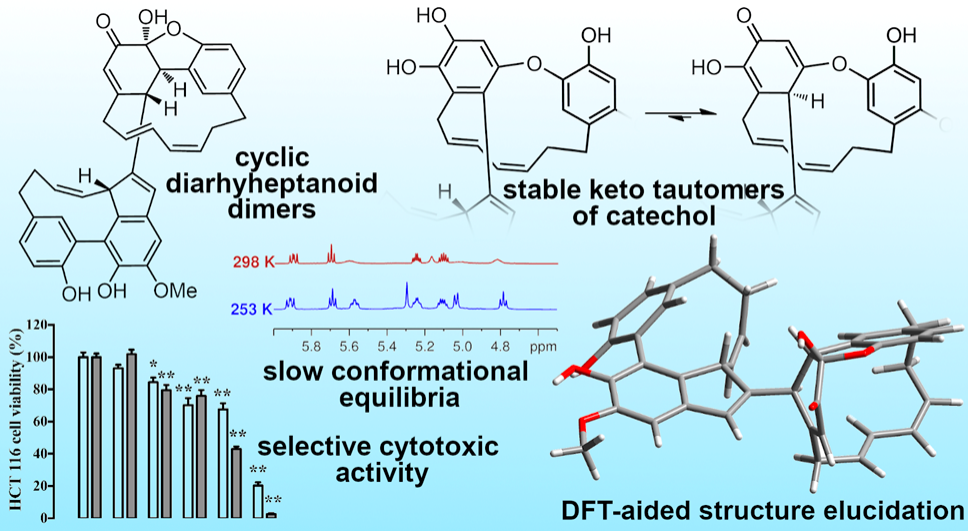

Two diarylheptanoid heterodimers, zosterabisphenones A (**1**) and B (**2**), were isolated from the seagrass *Zostera marina*. They feature unprecedented catechol keto tautomers, stable because of steric constraints. Their structure elucidation was based on extensive low-temperature NMR studies and ECD and MS data, with the essential aid of DFT prediction of NMR and ECD spectra. Zosterabisphenone B (**2**) was selectively cytotoxic against the adenocarcinoma colon cancer cell line HCT116 with IC_50_ 3.6 ± 1.1 μM at 48 h.

Diarylheptanoids are a class of natural products characterized by two benzene rings, usually bearing one or more hydroxyl groups, joined by a functionalized seven-carbon chain. Diarylheptanoids are widespread in plants, curcumin being the best known and commercially most important example.^1^ A smaller subset of diarylheptanoids comprises cyclic diarylheptanoids, in which the two aromatic rings are linked together directly (biphenyl type) or through an oxygen atom (diphenyl ether type). Due to their inherent steric strain, cyclic diarylheptanoids are frequently found to possess axial and/or planar chirality.^2,3^ Indeed, the smallest natural product that contains axial, planar, and point chirality elements in the same molecule is the cyclic diarylheptanoid tedarene B.^4^ When the energy barrier between the atropisomers is relatively low, axial or planar chirality is known to cause coalescent NMR signals.^4^

A number of diarylheptanoids have recently been reported from the common eelgrass, *Zostera marina* L. (Zosteraceae).^5,6^ These include zosteraphenol A (**3**) and B (**4**), two tetracyclic diarylheptanoids that experience equilibrium with minor atropisomers with opposite axial chiralities, resulting in ^1^H and ^13^C NMR spectra rich in coalescent signals.^6^ The nature of the rotameric equilibrium of zosteraphenols was fully determined using a combination of variable-temperature NMR measurements and DFT calculations.^6^

Here we report the isolation and structure elucidation of two unique diarylheptanoid dimers, zosterabisphenones A (**1**) and B (**2**) (Chart 1), putatively originating from oxidative coupling of two different cyclic diarylheptanoids. In both zosterabisphenones, one of the benzene rings is highly modified and is no longer aromatic, due to a tautomeric equilibrium disfavoring a catechol compared to its keto tautomer.

**Chart 1:**
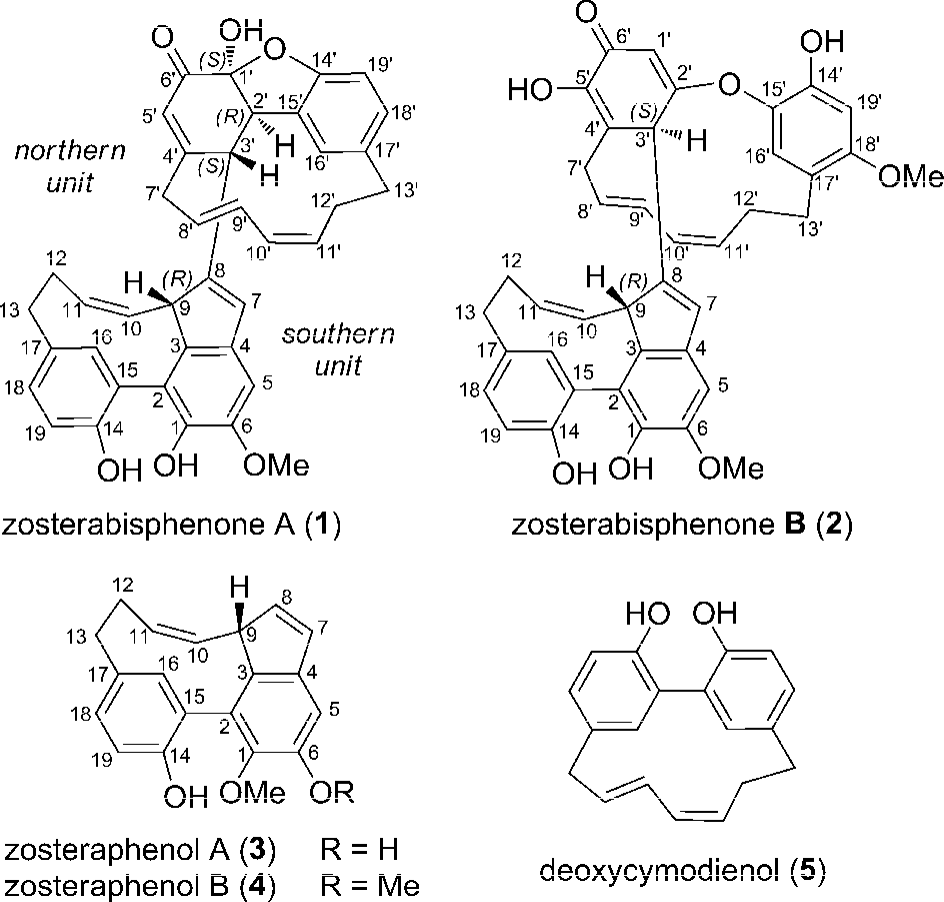


*Z. marina* (unrooted plants, freshly washed ashore and air-dried) was extracted with acetone, and the extract was subjected, in sequence, to SiO_2_ column chromatography, Sephadex LH-20 chromatography, and reversed-phase HPLC to give pure zosterabisphenone A (**1**, 4.6 mg) and B (**2**, 4.2 mg).

The molecular formula of zosterabisphenone A (**1**) was determined as C_39_H_34_O_6_ (23 unsaturations) from the [M + Na]^+^ ion at *m*/*z* 621.2235 in the high-resolution ESI mass spectrum and hinted to a dimeric diarylheptanoid structure. All NMR experiments of compound **1** were recorded at low temperature (253 K), because the ^1^H and ^13^C spectra recorded at room temperature showed many coalescent signals (Figure S10), similarly to the monomeric diarylheptanoids zosteraphenols A (**3**) and B (**4**) from the same source taxon.^6^ Indeed, examination of NMR data (Table S1) showed that one of the diarylheptanoid units (“southern unit”) was similar to zosteraphenol A (**3**), except that C-8 was not protonated, and therefore was supposed to be involved in linking the other diarylheptanoid unit. Another difference was that the methoxy group of compound **1** was located at C-6, and not at C-1 as in zosteraphenol A (**3**).

Signals of the second (“northern”) unit were indicative of the presence of a 1,2,4-trisubstituted benzene ring and of a hepta-2,4-diene-1,7-diyl chain. The remaining six carbons in the molecule, including two *sp*^3^ methine carbons and a carbonyl carbon atom, suggested an extensive modification of the second benzene ring of the northern unit. Extensive analysis of HMBC data (shown in Figure 1 and discussed in detail in the Supporting Information section) revealed that zosterabisphenone A (**1**) features an unprecedented cyclohexenedione tautomer of catechol, in which one carbonyl is involved in a cyclic hemiacetal function with the OH group at position 14′. Moreover, HMBC data established the C-8/C-3′ bond connecting the two diarylheptanoid units.

**Figure 1:**
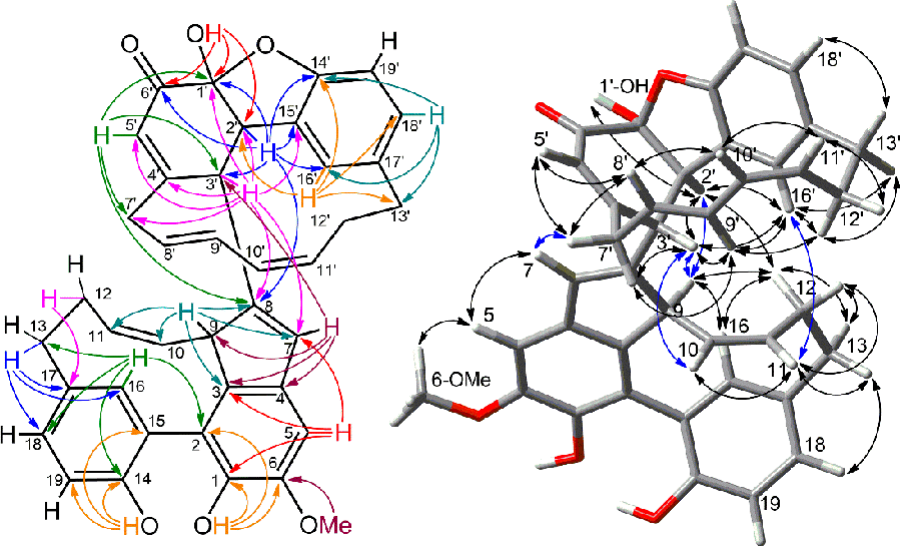
Most significant 2D NMR data of zosterabisphenone A (**1**). Left: HMBC correlations. Right: ROESY correlations.

The relative configuration of the three stereocenters on the northern unit (C-1′, C-2′, and C-3′) was determined by the ROESY correlation between OH-1′ and H-2′, pointing to their *cis* relationship, and by the ROESY correlations of H-3′ with H-9′ and H-16′, showing H-3′ pointing inward of the macrocycle and therefore establishing the relative configuration at C-3′ (Figure 1). However, the stereochemical relationship between the northern unit and the southern unit, also containing one stereocenter, could not be determined from NMR data. Therefore, structure elucidation of zosterabisphenone A (**1**) was completed with a detailed DFT study performed using the Gaussian 16 program (Revision C.01, Gaussian Inc., Wallingford CT, USA). This computational work, summarized below and described in detail in the Supporting Information section, further supported the structural features determined from NMR data and allowed the elucidation of the relative configuration between the two diarylheptanoid units and of the absolute configuration of the whole molecule.

An initial DFT study on the model compound **1n** (see Supporting Information) showed that the northern diarylheptanoid unit can adopt only one low-energy conformation. Based on this information and on the conformation of the southern unit determined in the previous work,^6^ models were generated for two diastereomers of compound **1**, differing in the relative configuration between the diarylheptanoid units, namely, (9*R*,1′*S*,2′*R*,3′*S*)-**1** (called just **1** in the following text) and (9*S*,1′*S*,2′*R*,3′*S*)-**1** (*epi*-**1** in the following text).

Conformation around the rotable bond C-8/C-3′, connecting the two units, was not obvious from spectroscopic data. Therefore, torsion about this bond was scanned in steps of 10°, and the resulting structures were optimized at the B3LYP/6-31G(d) level. This identified (Figure S2) one low-energy conformer for **1** and two low-energy conformers, separated by a nearly flat potential profile, for *epi*-**1**. The conformers were reoptimized at the B3LYP/6-31+G(d,p) level, giving the final structures that were used for NMR and ECD prediction.

Prediction of the ^1^H and ^13^C NMR chemical shifts^7^ allowed a confident selection between the alternative diastereomers **1** and *epi*-**1**. Isotropic shieldings were calculated^8^ at the PBE0/6-311+G(2d,p) level of theory, including the PCM continuous solvent model for chloroform,^9^ and were converted to chemical shifts using the conversion factors proposed by the Tantillo group^10^ for this level of theory (for *epi*-**1**, the Boltzmann-averaged chemical shifts over the two conformers were considered). Diastereomer **1** matched experimental chemical shifts remarkably better (RMSD of 1.66 ppm for ^13^C and 0.113 ppm for ^1^H) than *epi*-**1** (RMSD of 1.92 ppm for ^13^C and 0.148 ppm for ^1^H). In addition, some predicted chemical shifts of *epi*-**1** (C-9, H-5′, and H-7′ )showed large deviations (Figure S3). Finally, DP4+ analysis^11^ of the predicted chemical shifts showed a 100.00% probability for **1** to be the correct stereoisomer.

DFT prediction of NMR parameters also provided a solid support to the unique structure of zosterabisphenone A (**1**),^12^ in that the accuracy of the prediction was remarkably better than the expected accuracy of the method (reported as 2.45 for ^13^C and 0.15 ppm for ^1^H)^10^ and similar to the accuracy obtained for zosteraphenol A in our previous work.^6^ Further support to structure, configuration, and conformation of the two diarylheptanoid units of **1** came from DFT prediction of ^1^H–^1^H scalar coupling, calculated according to the suggestions of Bally and Rablen.^13^ The predicted couplings were in excellent agreement with the observed multiplicity of ^1^H NMR signals (Table S12); in particular, the predicted coupling between the vicinal protons H-2′ and H-3′ (1.1 Hz), in turn linked to the −82.5° torsion angle between them, nicely fit the singlet resonance observed for the two protons. Finally, all the observed ROESY cross peaks (Figure 1) were associated with protons which are spatially close in the determined DFT minimum energy conformation.

Absolute configuration of zosterabisphenone A was determined by DFT prediction of its ECD spectrum^14^ at the ωB97XD/6-31+G(d,p) level of theory, using the PCM model for the solvent. The predicted ECD spectrum, generated using the SpecDis program,^15^ was in good agreement with the experimental ECD spectrum (Figure S17), thus defining the (9*R*,1′*S*,2′*R*,3′*S*) absolute configuration for zosterabisphenone A.

The reason for the stability of the enone tautomer **1** compared to its aromatic tautomer **1a** (Scheme 1) can be ascribed to steric reasons. In the catechol tautomer **1a**, the bulky southern unit at C-4′ and the C_7_ bridge would collide unless the aromatic ring largely deviates from planarity. Indeed, the DFT energy of the aromatic tautomer **1a** was found to be 7.95 kcal/mol higher than that of **1**, and in the optimized structure of **1a** the dihedral angle for the two ortho bonds at C-2′ and C-3′ (C-15′/C-2′/C-3′/C-8) was as large as 40° (Figure S8 and Table S7). The steric strain is much lower in the enone tautomer **1**, where the C-3′/C-4′ single bond poses no constraints on a C-15′/C-2′/C-3′/C-8 dihedral angle of 90°. Moreover, the *sp*^3^ hybridization of the two atoms at the ring junction C-1′ and C-2′ further reduces the steric strain of the northern cyclic diarylheptanoid unit because it allows the cyclohexenone and the benzoxolane planes to be nearly perpendicular (Figures 1 and S2). The release of steric strain in **1** compared to **1a** was estimated as 25.1 kcal/mol using the isodesmic reaction shown in Scheme S1.

**Scheme 1:**
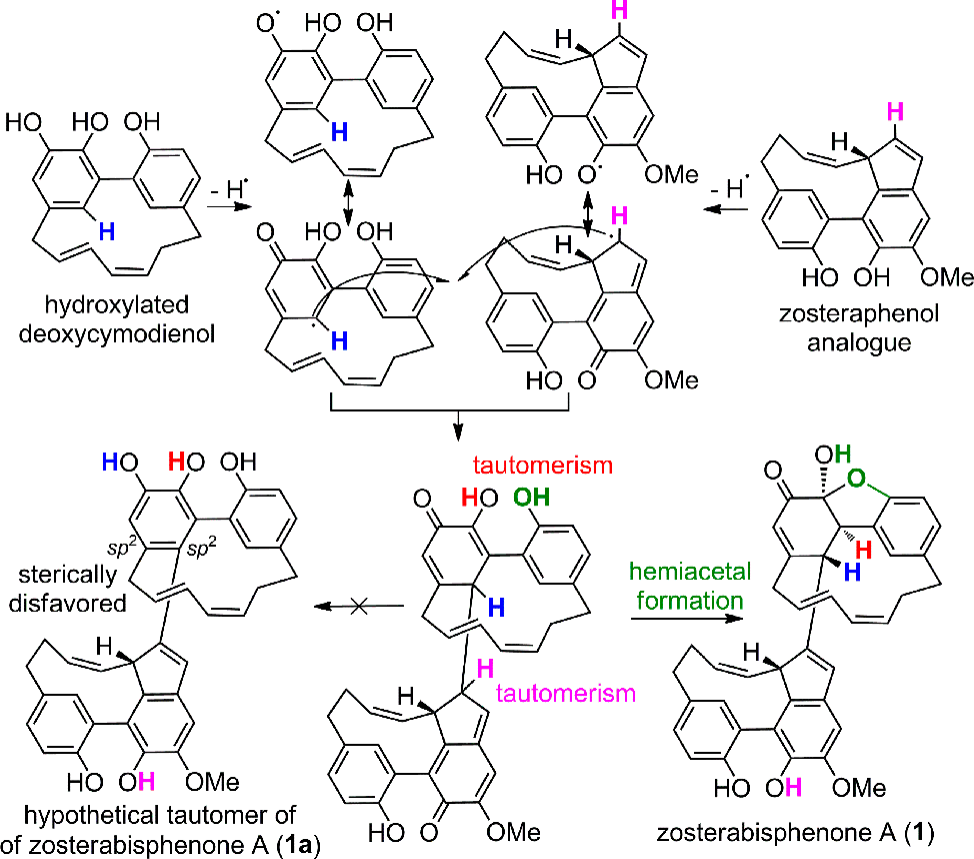
Putative Biosynthetic Pathways of Zosterabisphenone A (**1**)

Zosterabisphenone A presumably originates from the coupling of a zosteraphenol A analogue with a free 1-OH and a hydroxylated analogue of deoxycymodienol (**5**). A possible mechanism for its biosynthesis (Scheme 1) involves radical oxidative coupling, followed by tautomerism to enedione, and hemiacetal formation. This mechanism requires a free OH group at C-1. Interestingly, the two monomeric zosteraphenols A and B isolated from *Z. marina* have both a methoxy group at C-1 and therefore cannot react in this way.

The molecular formula of zosterabisphenone B (**2**) was determined as C_40_H_36_O_8_ ([M + H]^+^ at *m*/*z* 645.2471, 23 unsaturations), with one more carbon atom compared to zosterabisphenone A, in accordance with the presence of an additional methoxy group evidenced by the ^1^H NMR spectrum. Coalescent signals were present in the ^1^H and ^13^C NMR spectra of **2** recorded at room temperature and persisted at 253 K; only at 238 K all signals were sharp enough to allow for structure elucidation. The southern diarylheptanoid unit was the same as in zosterabisphenone A, with similar ^1^H and ^13^C chemical shifts (Table S2). The northern unit contained a hepta-2,4-diene-1,7-diyl chain, easily identified from the COSY spectrum, as in zosterabisphenone A. However, analysis of HMBC correlation peaks (Figure 2), including magnitude of ^1^H–^13^C couplings^16^ (see Supporting Information for details), showed that the 1,2,4-trisubstituted benzene ring of zosterabisphenone A was replaced by a 1,2,4,5-tetrasubstituted, trioxygenated benzene ring, and that the second ring in the northern diarylheptanoid unit was a cross-conjugated cyclohexadienone. Further, C-2′ and C-15′ were not linked directly as in zosterabisphenone A but were connected through an ether bridge. This structural assignment and the overall correctness of structure **2** were later supported by DFT chemical shift prediction (see below) and by ^1^H–^1^H scalar coupling prediction (Table S13), both showing an excellent agreement with the experiment.

**Figure 2:**
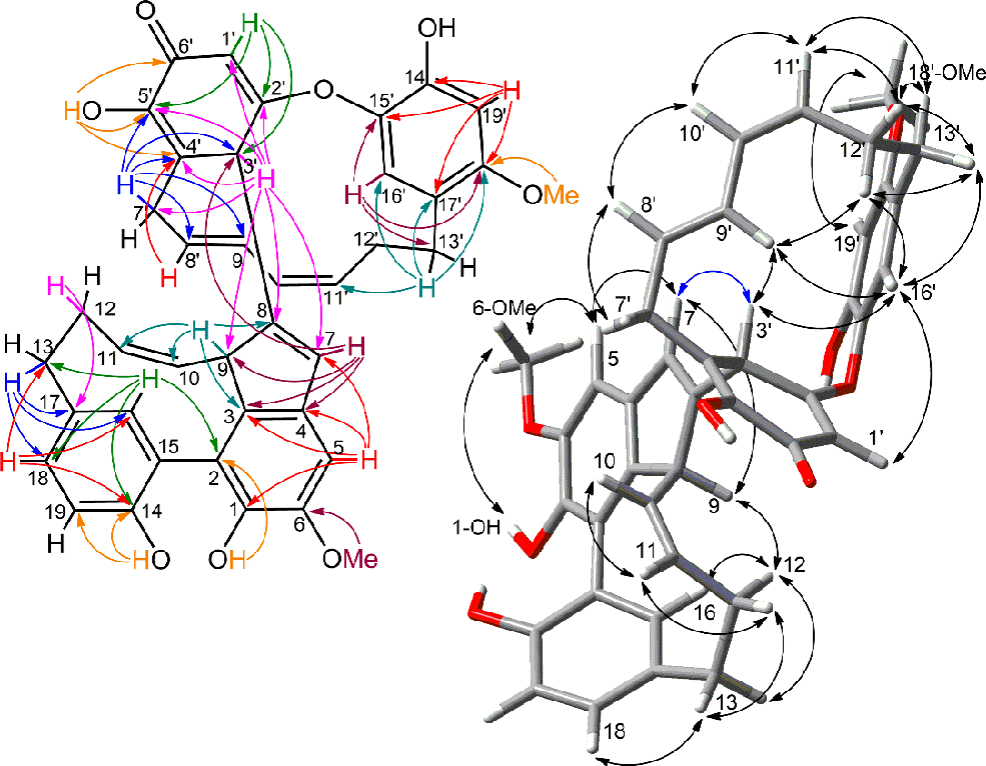
Most significant 2D-NMR data of zosterabisphenone B (**2**). Left: HMBC correlations. Right: ROESY correlations.

Zosterabisphenone B (**2**) contains two stereocenters, C-9 and C-3′, one on each diarylheptanoid unit. Their relative configuration was determined using the same protocol as described for compound **1**, involving preparation of DFT-optimized models of the northern diarylheptanoid unit in accordance with the observed NOEs (see Supporting Information for details), assembly of the two possible diastereomers **2** [the (9*R*,3′*S*) stereoisomer] and *epi*-**2** [the (9*R*,3′*R*) stereoisomer] and their DFT optimization; scan of possible conformers about the C-8/C-3′ bond (Figure S6), and reoptimization of the two conformers found for each diastereomer. The ^1^H and ^13^C chemical shifts of **2** and *epi*-**2** were calculated and compared with experimental data. While the accuracy of the predicted ^13^C chemical shifts was similar (RMSD of 2.05 ppm for **2** and 2.08 for *epi*-**2**), the accuracy of ^1^H chemical shifts was clearly better for **2** (RMSD of 0.126 ppm for **2** and 0.149 for *epi*-**2**) (Figure S7). Consistently, DP4+ analysis provided a 100.00% probability of **2** being the correct stereoisomer. Finally, absolute configuration of zosterabisphenone B (**2**) was determined by prediction of the ECD spectrum. The predicted spectrum of (9*R*,3′*S*)-**2** was in good agreement with the experimental ECD spectrum of **2** (Figure S27).

Like zosterabisphenone A, zosterabisphenone B (**2**) is a stable keto tautomer of a catechol, and again its stability can be ascribed to the unfavorable steric interactions of the hypothetical aromatic tautomer **2a** (Chart S1). Another intriguing structural feature of zosterabisphenone B is the very unusual *meta*,*meta* ether bridge present in its northern unit, compared to the *meta*,*para* coupling present in virtually all the known ether-bridged cyclic diarylheptanoids,^3^ including isotedarene A also found in *Z. marina*.^5^ Only one cyclic diarylheptanoid with a *meta*,*meta* ether bridge has been reported so far in the literature,^17^ plus another whose structure has been later revised to *meta*,*para*.^18^ Further, the absence of an OH group at C-1′ should be noted, because the carbon *para* to the C_7_ chain is normally oxygenated in diarylheptanoids.^1^ Because of these structural peculiarities, the biosynthesis of zosterabisphenone B (**2**) and the structure of the monomeric precursor of its northern unit remain obscure.

To assess the antitumor activity of zosterabisphenones, their effects on the cell viability of two cell lines (HCT116 and Hep G2 cells) were evaluated by using the MTT assay.^19^ Zosterabisphenone B (**2**) reduced, in a concentration- and time-dependent manner, the viability rate of HCT116 cells up to 48 h of exposure, reaching 97.4% inhibition at 10 μM (IC_50_ 3.9 ± 1.2 μM at 24 h, 3.6 ± 1.1 μM at 48 h). Conversely, it affected the Hep G2 cell viability only at the highest concentration tested, thus suggesting a selective effect on HCT116 cells compared to Hep G2 (Figure 3, Table S17). Zosterabisphenone A (**1**) showed weak cytotoxic effects on HCT116 only at the highest concentration and time period tested, and no effects on Hep G2 (Figure S28, Table S16).

**Figure 3:**
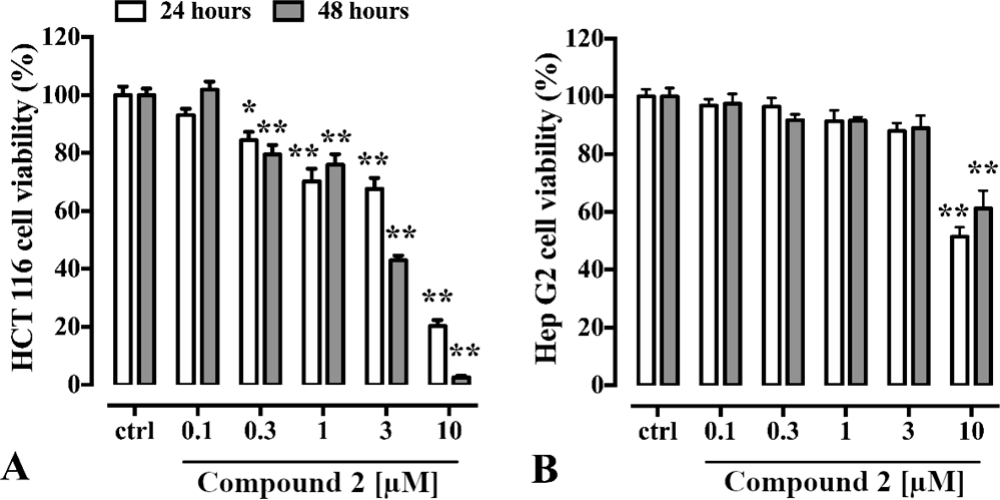
Cytotoxic effects of zosterabisphenone B (**2**) (0.1–10 μg/mL, 24 h and 48 h exposure) on HCT116 (A) and Hep G2 (B) cells. Cell viability rate (expressed as percentage) was investigated by using the MTT assay. Each bar represents the mean ± SEM of three independent experiments (including 5–6 replicates for each treatment). * *p* < 0.01 and ** *p* < 0.0001 vs control (ctrl, i.e., untreated cells).

The seagrass *Z. marina* is a common and easily accessible seagrass that has been studied by many groups so far, and it is surprising that the presence of such a variety of diarylheptanoids has been undetected until now. The conformational equilibria experienced by many of them, leading to coalescent signals in NMR experiments performed at room temperature, may have had a role in this. Because of its selective cytotoxic effects on HCT116 cells and the abundance of its natural source, zosterabisphenone B (**2**) can be proposed as a lead compound for the development of new antitumor drugs in colorectal cancer. Further experiments to investigate its selectivity (tumor cells vs normal cells) and evaluate its mechanism of action are in progress and will be reported in the due course.

## Supporting Information Available

Additional details on structure elucidation; experimental methods and characterization data; computational methods; computational results with additional figures (reactions, structures, and cytotoxicity data); tables with NMR data, Cartesian coordinates, rotatory strengths, and cell viability of **1** and **2**; ESI, NMR, HSQC, HMBC COSY, ROESY, and UV spectra of **1** and **2** (PDF)
