# Supplementary material for: Stable Catechol Keto Tautomers in Cytotoxic Heterodimeric Cyclic Diarylheptanoids from the Seagrass Zostera marina
Source: Org Lett. 2021 Sep 7;23(18):7134–8. doi: 10.1021/acs.orglett.1c02537 (PMC8453622; doi:10.1021/acs.orglett.1c02537)
Supplement: Supplementary file 1 — ol1c02537_si_001.pdf [file ol1c02537_si_001.pdf]

Supporting Information for

Stable catechol keto tautomers in cytotoxic heterodimeric cyclic diarylheptanoids from the seagrass *Zostera marina*

Yan Li,[†,‡] Laura Grauso,[§,‡] Silvia Scarpato,[⊥] Nunzio Antonio Cacciola,[#] Francesca Borrelli,[⊥] Christian Zidorn,[†] and Alfonso Mangoni*[,⊥]

[†] Pharmazeutisches Institut, Abteilung Pharmazeutische Biologie, Christian-Albrechts-Universität zu Kiel, Gutenbergstraße 76, 24118, Kiel, Germany
[§] Dipartimento di Agraria, Università degli Studi di Napoli Federico II, Via Università 100, 80055 Portici (NA), Italy
[⊥] Dipartimento di Farmacia, Università degli Studi di Napoli Federico II, Via D. Montesano 49, 80131 Napoli, Italy
[#] Dipartimento di Medicina Veterinaria e Produzioni Animali, Università degli Studi di Napoli Federico II, Via Via F. Delpino, 80137 Napoli, Italy
[‡] These authors contributed equally to this work.

Table of Contents

**Details of structure elucidation of zosterabisphenone A (1)**		2
**Details of structure elucidation of zosterabisphenone B (2)**		4
**Experimental Section**		7
**Chart S1**	The hypothetical aromatic tautomers of zosterabisphenone A (**1a**) and of zosterabisphenone B (**2a**).	10
**Scheme S1**	The isodesmic reaction used to estimate the release of steric strain in **1** compared to **1a**	10
**Figure S1**	The model compound **1n** for the northern diarylheptanoid unit of zosterabisphenone A	2
**Figure S2**	DFT energy of zosterabisphenone A (**1**) and its C-9 epimer *epi*-**1** as a function of the torsion angle about the C-9/C-8/C-3'/C-2' bond	3
**Figure S3**	Deviations of calculated ^{13}C and ^{1}H NMR chemical shifts of zosterabisphenone A (**1**) and its C-9 epimer *epi*-**1**	4
**Figure S4**	Sections of the ^{13}C-coupled HMBC spectrum of zosterabisphenone B (**2**) used to estimate the magnitude of ^{1}H-^{13}C coupling constants of H-16' and H-19'	5
**Figure S5**	The model compound **2n** for the northern diarylheptanoid unit of unit of zosterabisphenone B	5
**Figure S6**	DFT energy of zosterabisphenone B (**2**) and its C-9 epimer *epi*-**2** as a function of the torsion angle about the C-9/C-8/C-3'/C-2' bond	6
**Figure S7**	Deviations of calculated ^{13}C and ^{1}H NMR chemical shifts of zosterabisphenone B (**2**) and its C-9 epimer *epi*-**2**	7
**Figure S8**	Minimum energy conformation of the aromatic tautomer **1a** of zosterabisphenone A	11
**Figure S9**	High-resolution ESI mass spectrum of zosterabisphenone A (**1**)	11
**Figure S10**	^{1}H NMR spectrum of zosterabisphenone A (**1**) recorded at 298 K and 253 K (700 MHz, $CDCl_3$)	12
**Figure S11**	^{13}C NMR spectrum of zosterabisphenone A (**1**) recorded at 253 K (175 MHz, $CDCl_3$)	13
**Figure S12**	HSQC spectrum of zosterabisphenone A (**1**) recorded at 253 K (700 MHz, $CDCl_3$)	14
**Figure S13**	HMBC spectrum of zosterabisphenone A (**1**) recorded at 253 K (700 MHz, $CDCl_3$)	15
**Figure S14**	Expansion of the HMBC spectrum of zosterabisphenone A (**1**) recorded at 253 K (700 MHz, $CDCl_3$)	16
**Figure S15**	COSY spectrum of zosterabisphenone A (**1**) recorded at 253 K (700 MHz, $CDCl_3$)	17
**Figure S16**	ROESY spectrum of zosterabisphenone A (**1**) recorded at 253 K (700 MHz, $CDCl_3$)	18
**Figure S17**	Calculated and experimental UV and ECD spectra of zosterabisphenone A (**1**) in acetonitrile	19
**Figure S18**	High-resolution ESI mass spectrum of zosterabisphenone B (**2**)	20
**Figure S19**	^{1}H NMR spectrum of zosterabisphenone B (**2**) recorded at 298 K and 238 K (700 MHz, $CDCl_3$)	21
**Figure S20**	^{13}C NMR spectrum of zosterabisphenone B (**2**) recorded at 238 K (175 MHz, $CDCl_3$)	22
**Figure S21**	HSQC spectrum of zosterabisphenone B (**2**) recorded at 238 K (700 MHz, $CDCl_3$)	23
**Figure S22**	HMBC spectrum of zosterabisphenone B (**2**) recorded at 238 K (700 MHz, $CDCl_3$)	24
**Figure S23**	Expansion of the HMBC spectrum of zosterabisphenone B (**2**) recorded at 238 K (700 MHz, $CDCl_3$)	25
**Figure S24**	Expansion of the HMBC spectrum of zosterabisphenone B (**2**) recorded at 238 K (700 MHz, $CDCl_3$)	26
**Figure S25**	COSY spectrum of zosterabisphenone B (**2**) recorded at 238 K (700 MHz, $CDCl_3$)	27
**Figure S26**	ROESY spectrum of zosterabisphenone B (**2**) recorded at 238 K (700 MHz, $CDCl_3$)	28
**Figure S27**	Calculated and experimental UV and ECD spectra of zosterabisphenone B (**2**) in acetonitrile	29
**Figure S28**	Cytotoxic effects of zosterabisphenone A (**1**) and B (**2**) on HCT116 and Hep G2 cells	30
**Table S1**	^{1}H and ^{13}C NMR data of zosterabisphenone A (**1**) (700 MHz, 253 K, $CDCl_3$)	31
**Table S2**	^{1}H and ^{13}C NMR data of zosterabisphenone B (**2**) (700 MHz, 238 K, $CDCl_3$)	32
**Table S3**	Cartesian coordinates of the lowest-energy conformer of zosterabisphenone A (**1**)	33
**Table S4**	Cartesian coordinates of the lowest-energy conformers of the C-9 epimer of zosterabisphenone A (*epi*-**1**)	34
**Table S5**	Cartesian coordinates of the lowest-energy conformers of zosterabisphenone B (**2**)	35
**Table S6**	Cartesian coordinates of the lowest-energy conformers of the C-9 epimer of zosterabisphenone B (*epi*-**2**)	36
**Table S7**	Cartesian coordinates and predicted chemical shifts of the lowest-energy conformer of the aromatic tautomer of zosterabisphenone A (**1a**)	37
**Table S8**	Experimental and predicted chemical shifts of zosterabisphenone A (**1**)	38
**Table S9**	Experimental and predicted chemical shifts of the two conformers of the C-9 epimer of zosterabisphenone A (*epi*-**1**)	39
**Table S10**	Experimental and predicted chemical shifts of the two conformers of zosterabisphenone B (**2**).	40
**Table S11**	Experimental and predicted chemical shifts of the two conformers of the C-9 epimer of zosterabisphenone B (*epi*-**2**)	41
**Table S12**	Experimental multiplicity of ^{1}H NMR signals and predicted ^{1}H-^{1}H *J* couplings of zosterabisphenone A (**1**)	42
**Table S13**	Experimental multiplicity of ^{1}H NMR signals and predicted ^{1}H-^{1}H *J* couplings of zosterabisphenone B (**2**)	43
**Table S14**	Rotatory strengths (length formalism) calculated for zosterabisphenone A (**1**)	44
**Table S15**	Rotatory strengths (length formalism) calculated for the two conformers of zosterabisphenone B (**2**)	45
**Table S16**	Effects of zosterabisphenone A (**1**) on the viability of HCT116 and Hep G2 cells	47
**Table S17**	Effects of zosterabisphenone B (**2**) on the viability of HCT116 and Hep G2 cells	47
**References**		48

Details of structure elucidation of zosterabisphenone A (**1**)

The molecular formula of zosterabisphenone A (**1**) was determined as $C_{39}H_{34}O_6$ (23 unsaturations) from the $[M+Na]^+$ ion at *m/z* 621.2235 in the high-resolution ESI mass spectrum and hinted to a dimeric diarylheptanoid structure. Examination of NMR data (Table S1) showed that one of the diarylheptanoid units ("southern unit") was similar to zosteraphenol A (**3**), except that C-8 was not protonated, and therefore was supposed to be involved in linking the other diarylheptanoid unit. Another difference was that the methoxy group of compound **1** was located at C-6, and not at C-1 as in zosteraphenol A (**3**). The southern unit, closely related to zosteraphenol A (**3**), showed the same conformational equilibrium previously observed for **3** and discussed in detail in ref. 6, leading to coalescence of many protons and carbons in the NMR spectra recorded at room temperature. In the spectra recorded at 253 K, where the conformational equilibrium was slower, all signals were sharp enough for structure elucidation.

Signals of the second ("northern") unit suggested the presence of a 1,2,4-trisubstituted benzene ring (C-14' to C-19'), similar to the corresponding ring in the southern unit, and of a hepta-2,4-diene-1,7-diyl chain (C-7' to C-13'). The linkage between C-13' and C-17' was clearly shown by HMBC correlations of H-16' and H-18' with C-13' (Table S1 and Figure 1 in the main text). The remaining six carbons in the molecule, including two sp^3 methine carbons and a carbonyl carbon atom, suggested an extensive modification of the second benzene ring of the northern unit. The sp^3 methine carbon at δ 50.2 (C-2') was linked to C-15', as indicated by the HMBC correlation of H-2' with C-14', C-15', and C-16'. The other sp^3 methine carbon at δ 46.1 (C-3') was linked to C-2', as established by the HMBC correlations of H-3' with C-2' and C-15'. The multiplicity of the vicinal protons H-2' and H-3', both resonating as singlets, was later explained by the dihedral angle close to 90° between the two protons. The remaining three sp^2 carbon atoms (δ 189.1, C, C-6'; δ 122.6, CH, C-5'; δ 163.3, C, C-4') suggested an α,β-unsaturated ketone, linked to C-3' through its β carbon atom based on the HMBC correlations of H-2' with C-4' and of H-3' with C-4' and C-5' (Figure 1).

The last carbon atom to be assigned was a non-protonated carbon resonating at δ 103.9 (C-1'). It was assigned as a hemiacetal carbon atom connecting C-2' with C-6' and defining a cyclohexenone ring, based on the HMBC correlations (among others) of the OH proton at 1' and C-1', C-2', and C-6'. The bond between C-4' and C-7', and the bond C-8/C-3' joining the two diarylheptanoid units, were supported by the HMBC correlations of H-7, H-2', H-3, and H-5' (Figure 1). Finally, the ether bridge between C-1' and C-14' could not be proven by HMBC, but was the only possible way to close the structure and satisfy the 23 unsaturations implied by the molecular formula.

Structure **1** contains four stereogenic centers, namely C-9, C-1', C-2', and C-3'. The relative configuration of the three stereocenters on the northern unit was determined by a ROESY correlation between OH-1' and H-2', pointing to their *cis* relationship, and by the ROESY correlations of H-3' with H-9' and H-16' establishing the relative configuration at C-3' (Figure 1). The relative configuration of the two diarylheptanoid units to each other and the absolute configuration of the whole molecule were determined by a detailed DFT study, which also fully supported the planar structure and the stereochemistry of the two diarylheptanoids.

The favored conformation of the southern diarylheptanoid unit, which is identical for all zosterabisphenones and is closely related to zosteraphenols, was known from the previous study on zosteraphenols and confirmed by ROESY data (Figure 1 in the main text and Table S1). Therefore, a model of the southern unit was generated starting from the lowest energy conformer zosteraphenol B (**4**) and removing the methyl group at O-1, followed by DFT optimization of the resulting molecule.

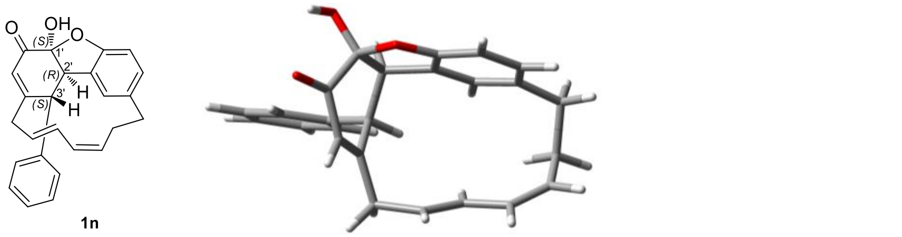

1n

Figure S1. The model compound **1n** for the northern diarylheptanoid unit of unit of zosterabisphenone A and its lowest-energy conformation at the B3LYP/6-31+G(d,p) level.

A model of the northern diarylheptanoid unit of zosterabisphenone A, with the southern unit replaced by a phenyl ring and with the relative configuration at C-1', C-2', and C-3' as determined by NMR studies (**1n**, Figure S1) was subjected to molecular dynamics (MD) conformational search (the 1'*S*,2'*R*,3'*S* enantiomer was tentatively used). The MD simulation was set at 2000 K to allow possible slow conformational changes to occur in the 10-ns duration of the simulation; previous studies[4] on tedarene B showed that this protocol can reproduce conformational changes with $k < 1\ s^{-1}$, and therefore can adequately sample the full conformational space of model compound **1n**. The 200 minimized structures produced by the conformational search protocol identified 16 unique conformers for **1n** in a range of 7.76 kcal/mol. The geometries of these conformers were then refined by DFT optimization at the B3LYP/6-31+G(d,p) level. This caused several conformers to converge, leaving only 7 unique conformers after geometry optimization. The geometry of the lowest-energy conformer was in full agreement with ROESY data (Table S1); in contrast, the second-lowest-energy conformer (ΔE = 1.42

kcal/mol) was not supported by the ROESY data; the remaining 5 conformers had high DFT energies (ΔE > 5 kcal/mol) and were not significantly populated at room temperature. Therefore, only the lowest energy conformation was considered for the northern unit.

Models for the two possible diastereomers of compound **1** were generated, namely (9*R*,1'*S*,2'*R*,3'*S*)-**1** (called just **1** in the following text) and (9*S*,1'*S*,2'*R*,3'*S*)-**1** (*epi*-**1** in the following text), differing in the relative configuration between the diarylheptanoid units. Conformation around the rotable bond C-8/C-3' was not obvious from spectroscopic data. Therefore, the dihedral angle C-9/C-8/C-3'/C-2' was scanned in steps of 10°, and the resulting structures were optimized at the B3LYP/6-31G(d) level.

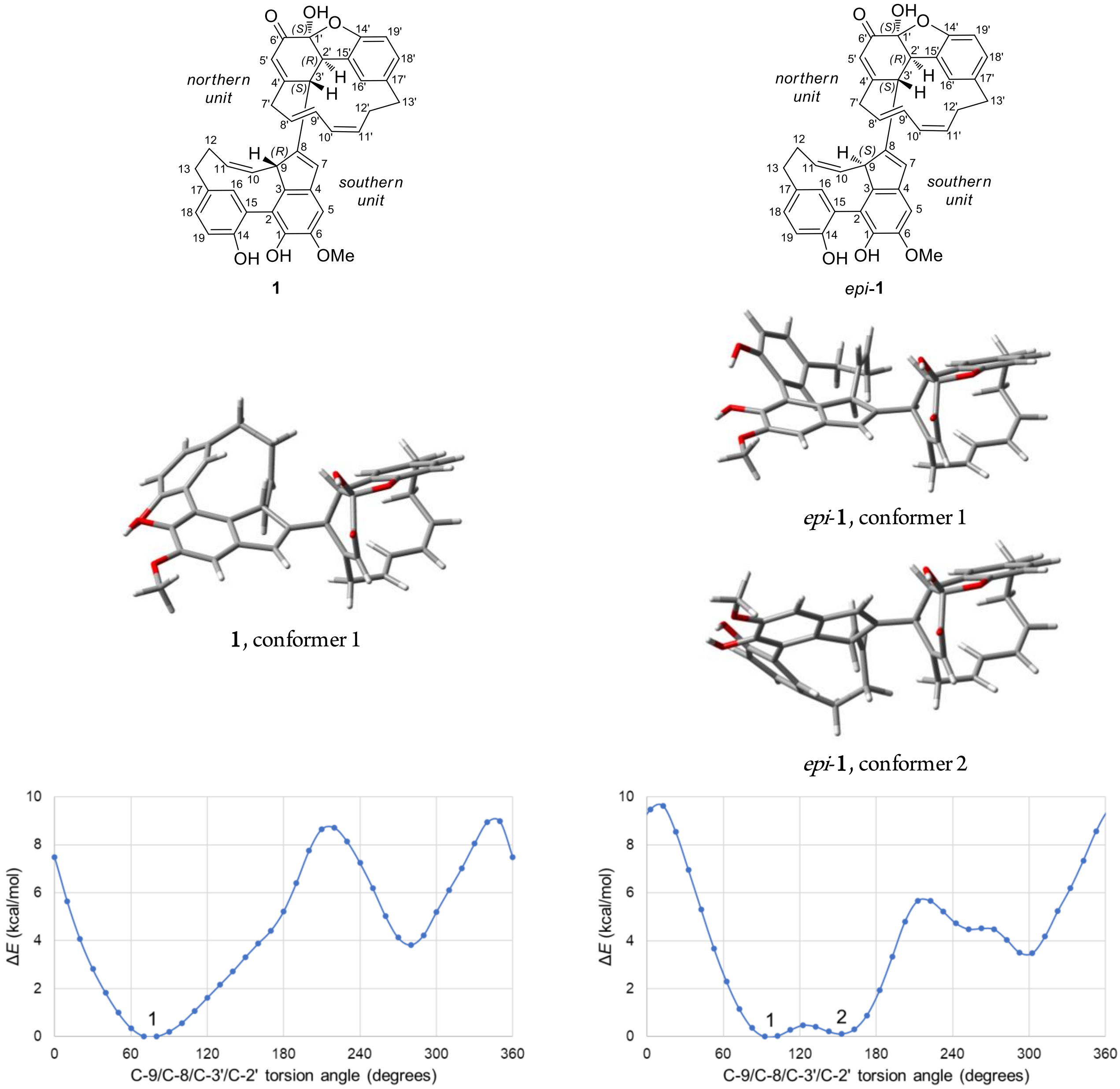

Figure S2. DFT energy of zosterabisphenone A (**1**) and its C-9 epimer *epi*-**1** as a function of the torsion angle about the C-9/C-8/C-3'/C-2' bond, i.e. the bond that connects the two cyclic diarylheptanoid units. The significantly populated minimum energy conformations are shown above the graphs.

Analysis of the results (Figure S2) showed for **1** the existence of two minima at 80° and 280°, the latter being 3.8 kcal/mol higher in energy, and therefore not significantly populated; for *epi*-**1**, three minima were found, two close low energy minima at 93° (ΔE = 0) and 153° (ΔE = 0.12 kcal/mol), separated by a nearly flat potential profile, and one high-energy minimum (ΔE = 3.5 kcal/mol). Finally, the low-energy conformer of **1** and the two low-energy conformers of *epi*-**1** were re-optimized at the B3LYP/6-31+G(d,p) level, and the resulting geometries (Table S3 and S4) were used for subsequent calculations.

NMR isotropic shieldings were calculated[8] at the PBE0/6-311+G(2d,p) level of theory, including the PCM continuous solvent model[9] for chloroform (Table S8 and S9; for *epi*-**1**, the Boltzmann-averaged isotropic shieldings over the two conformers were considered). Isotropic shieldings were converted to chemical shifts using the conversion factors determined by the Tantillo group[10] for this level of

theory (^{1}H: slope −1.0958, intercept 31.7532; ^{13}C: slope −1.0533, intercept: 187.3123; these and other scaling factors may also be found in the website http://cheshirenmr.info). Diastereomer **1** matched experimental chemical shifts better (RMSD of 1.66 ppm for ^{13}C and 0.113 ppm for ^{1}H) than *epi*-**1** (RMSD of 1.92 ppm for ^{13}C and 0.148 ppm for ^{1}H). In addition, some predicted chemical shifts of *epi*-**1** (C-9, H-5', and H-7') showed large deviations (Figure S3).

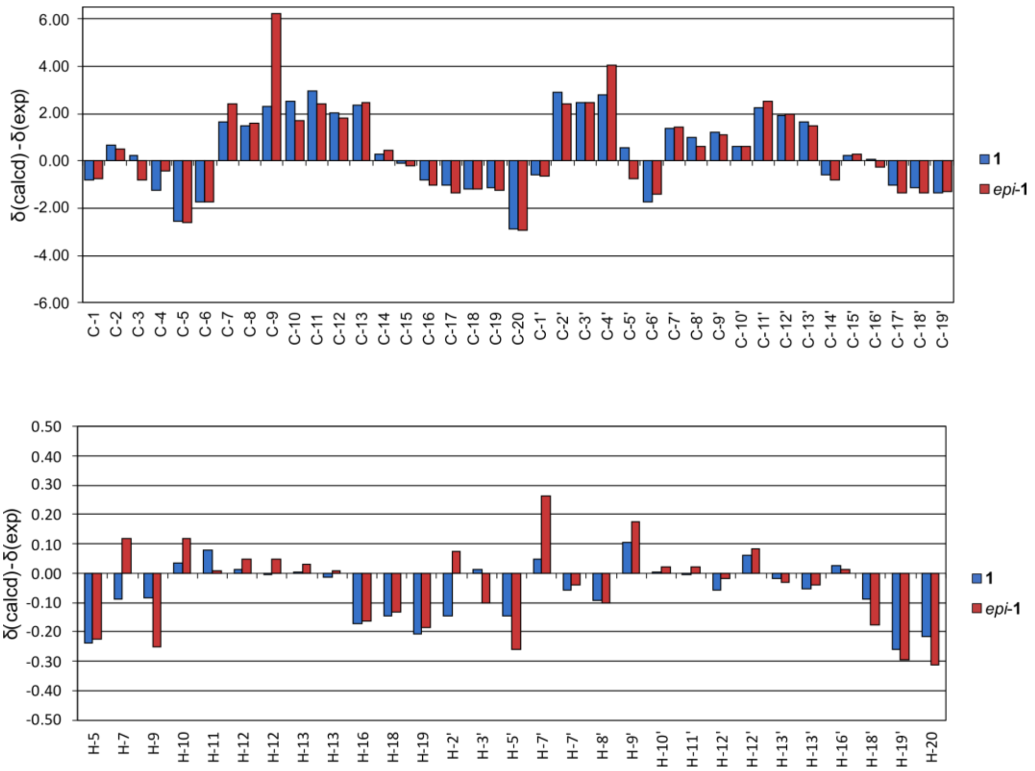

Figure S3. Deviations from experimental values of calculated ^{13}C (top panel) and ^{1}H (bottom panel) NMR chemical shifts of zosterabisphenone A (**1**) and its C-9 epimer *epi*-**1**.

Moreover, DP4+ analysis[11] of the predicted chemical shifts showed a 100.00% probability for **1** to be the correct stereoisomer. As a further support for structure and conformation of **1**, the ^{1}H-^{1}H scalar couplings were calculated according to the suggestions of Bally and Rablen,[13] i.e. considering only the Fermi contact terms calculated at the B3LYP/6-31G(d,p) level of theory in vacuo, and scaling them by a factor of 0.9117. The results are reported in Table S12.

The ECD spectrum of zosterabisphenone A was calculated[14] at the ωB97XD/6-31+G(d,p) level, with the PCM continuous model[9] for the solvent, acetonitrile, used for the experimental measurement (Table S14). The predicted spectrum was generated using the SpecDis program,[15] which was also used to optimize the half-band width σ (0.51 eV) and the UV correction (+27 nm). The predicted ECD spectrum was in good agreement with the experimental spectrum (Figure S17), thus defining the (9*R*,1'*S*,2'*R*,3'*S*) configuration for zosterabisphenone A.

Details of structure elucidation of zosterabisphenone B (**2**)

The molecular formula of zosterabisphenone B (**2**) was determined as $C_{40}H_{36}O_8$ ([M+H]$^+$ at *m/z* 645.2471, 23 unsaturations). All the ^{1}H and ^{13}C NMR spectra of **2** were recorded 238 K. The southern diarylheptanoid unit was identical to that present in zosterabisphenone A, with similar ^{1}H and ^{13}C chemical shifts (Table S2). Like for zosterabisphenones A and B, the northern unit contained a hepta-2,4-diene-1,7-diyl chain, easily identified from the COSY spectrum. The presence of a 1,2,4,5-tetrasubstituted, trioxygenated benzene ring was shown by the HMBC correlations of the two para protons H-16' and H-19' and of protons at C-13' (Figure 2 in the main text, Table S2). The assignment of the non-protonated carbon atoms of the ring was not trivial and was based on the different magnitude of $^{2}J_{CH}$ (1.0-3.0 Hz) and $^{3}J_{CH}$ (6.5-8.5 Hz) known to exist in aromatic rings.[16] The ^{1}H-^{13}C coupling constants of H-16 and H-19 could be estimated from the rows of the HMBC spectrum at δ 154.9, 144.7, 136.4, and 121.7, thus allowing assignments of these ^{13}C signals, respectively, to C-18', C-14', C-15', and C-17' (Figure S3).

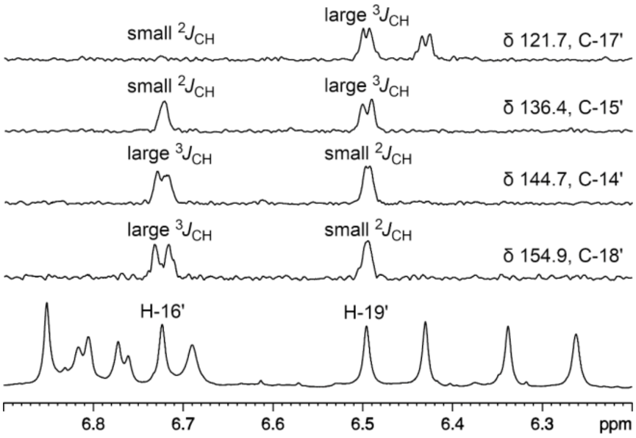

Figure S4. Sections of the ^{13}C-coupled HMBC spectrum of zosterabisphenone B (**2**) were used to estimate the magnitude of ^{1}H-^{13}C coupling constants of H-16' and H-19', and therefore to distinguish between the smaller $^2J_{CH}$ and the larger $^3J_{CH}$.

The second ring in the northern diarylheptanoid unit was a cross-conjugated cyclohexadienone. Unequivocal sequential assignment of the carbons of this ring was based on HMBC data (Figure 2 in the main text, Table S2) as detailed below. The cross peak of H-8' with C-4' assigned C-4' as the carbon linked to the C_7 chain. The cross peaks of H-7' with C-3', C-4', and C-5' assigned C-5' and the sp^3 carbon atom C-3' (δ 42.3). The cross peaks of OH-5' with C4', C-5', and C-6' assigned the carbonyl carbon atom C-6' (δ 183.2) and defined an α-hydroxy enone system. The cross peaks of H-3' with C-1', C-2', C-4', and C-5' assigned C-1' and C-2' and therefore defined the second double bond in the ring; C-2' was strongly deshielded (δ 175.4) by being β to the carbonyl group *and* linked to O. Finally, the cross peaks of H-1' with C-2', C-3', and C-5' confirmed previous assignments and closed the six-membered rings. Further HMBC correlations of H-3' with C-7, C-8, and C-9 clearly showed the C-8/-C-3' connection between the two diarylheptanoid units.

At this stage, only two of the oxygen atoms present in the molecular formula were still available for the three oxygenated carbon atoms C-2', C14', and C-15', and only one further OH signal (which unfortunately showed no HMBC or ROESY correlations) was present in the spectrum. This implied and ether bridge between C-2' and either C-14' or C-15'. The ether bridge was located between C-2' and C-15' because of the weak but clear NOESY cross peak between H-1' and H-16', which was geometrically impossible in the alternative structure. This structural assignment and the overall correctness of structure **2** were then supported by DFT chemical shift prediction (see below) and by ^{1}H-^{1}H scalar coupling prediction (Table S13), both showing an excellent agreement with the experiment. The relative configuration between the two diarylheptanoid units of zosterabisphenone B (**2**) was determined using a DFT-based approach similar to that described above for zosterabisphenone A (**2**).

A simplified model (**2n**) of the northern diarylheptanoid unit of zosterabisphenone B, with the southern unit replaced by a phenyl ring (Figure S5) was subjected to high-temperature MD conformational search (the 3'*S* enantiomer was tentatively used). This identified 29 unique conformers in a range of 11.54 kcal/mol, whose geometry was optimized by DFT at the B3LYP/6-31+G(d,p) level. The lowest-energy conformer was in full agreement with the ROESY data (Table S2); in contrast, the second-lowest energy conformer (ΔE = 1.70 kcal/mol) was not supported by the ROESY data; the remaining conformers had high DFT energies (ΔE > 4 kcal/mol). Therefore, only the lowest energy conformation was considered for the northern unit.

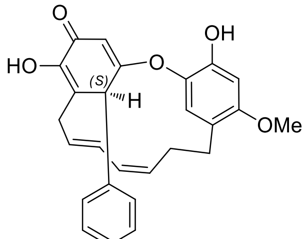

2n

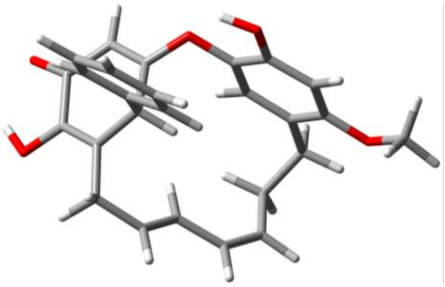

Figure S5. The simplified model compound **2n** for the northern diarylheptanoid unit of unit of zosterabisphenone B and its lowest-energy conformation at the B3LYP/6-31+G(d,p) level.

The two possible diastereomers of zosterabisphenone B were generated by replacing the phenyl group of **2n** with either enantiomer of the southern unit, to give **2** (the 9*R*,3'*S* stereoisomer) and *epi*-**2** (the 9*S*,3'*S* stereoisomer). The torsion angle around the C-8/C-3' bond was scanned as described above for **1**/*epi*-**1**. The resulting graph of the potential energy vs. dihedral angle (Figure S6) showed two low-energy conformers for **2** and two low-energy conformers for *epi*-**2**.

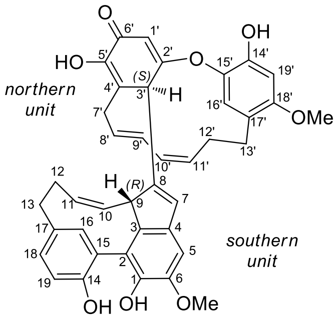

2

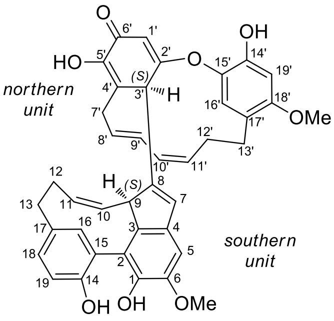

epi-**2**

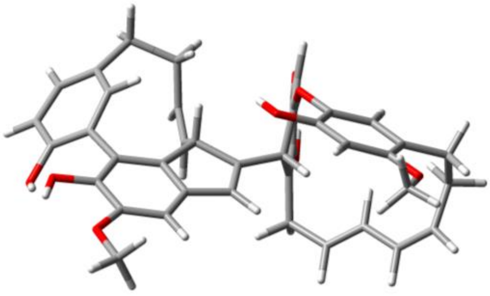

2, conformer 1

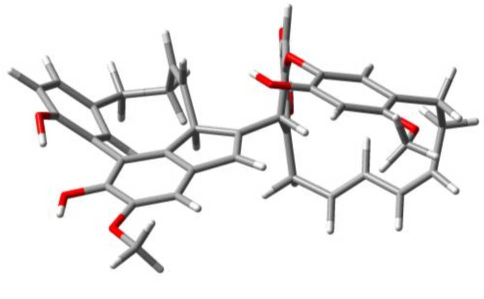

epi-**2**, conformer 1

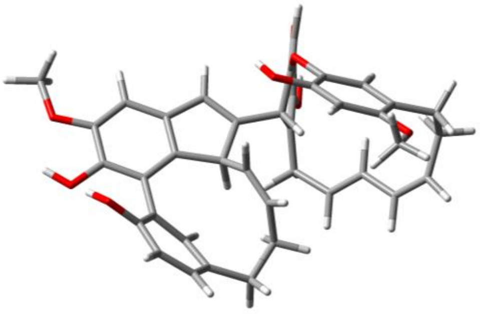

2, conformer 2

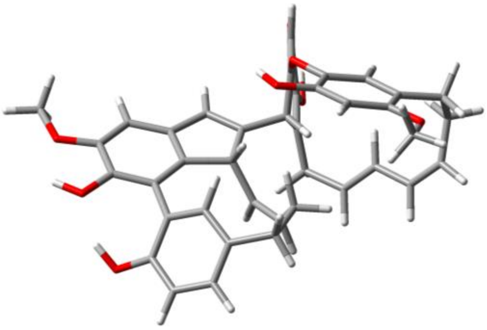

epi-**2**, conformer 2

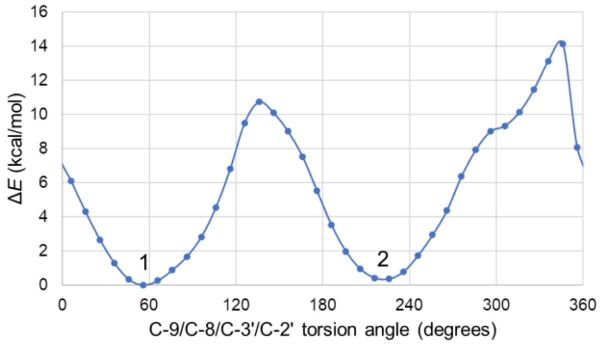

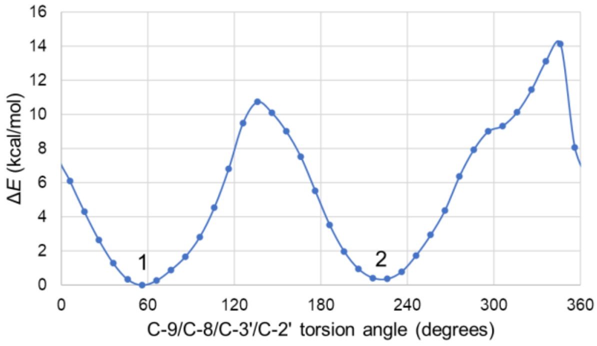

Figure S6. DFT energy of zosterabisphenone B (**2**) and its C-9 epimer *epi*-**2** as a function of the torsion angle about the C-9/C-8/C-3'/C-2' bond, i.e. the bond that connects the two cyclic diarylheptanoid units. The significantly populated minimum energy conformations are shown above the graphs.

The conformers of **2** and *epi*-**2** were reoptimized at the B3LYP/6-31+G(d,p) level, and the resulting geometries (Tables S5 and S6) were used for NMR and ECD prediction. NMR isotropic shieldings were calculated at the PBE0/6-311+G(2d,p)/PCM level, Boltzmann-averaged over the two conformers of each compound (Table S10 and S11), and converted to chemical shifts using the conversion factors discussed above. The accuracy of the predicted ^{13}C chemical shifts was similar (RMSD of 2.05 ppm for **2** and 2.08 for *epi*-**2**), but the accuracy of ^{1}H chemical shifts were clearly better for **2** (RMSD of 0.126 ppm for **2** and 0.149 for *epi*-**2**) (Figure S7). Consistently, DP4+ analysis provided a 100.00% probability of **2** being the correct stereoisomer.

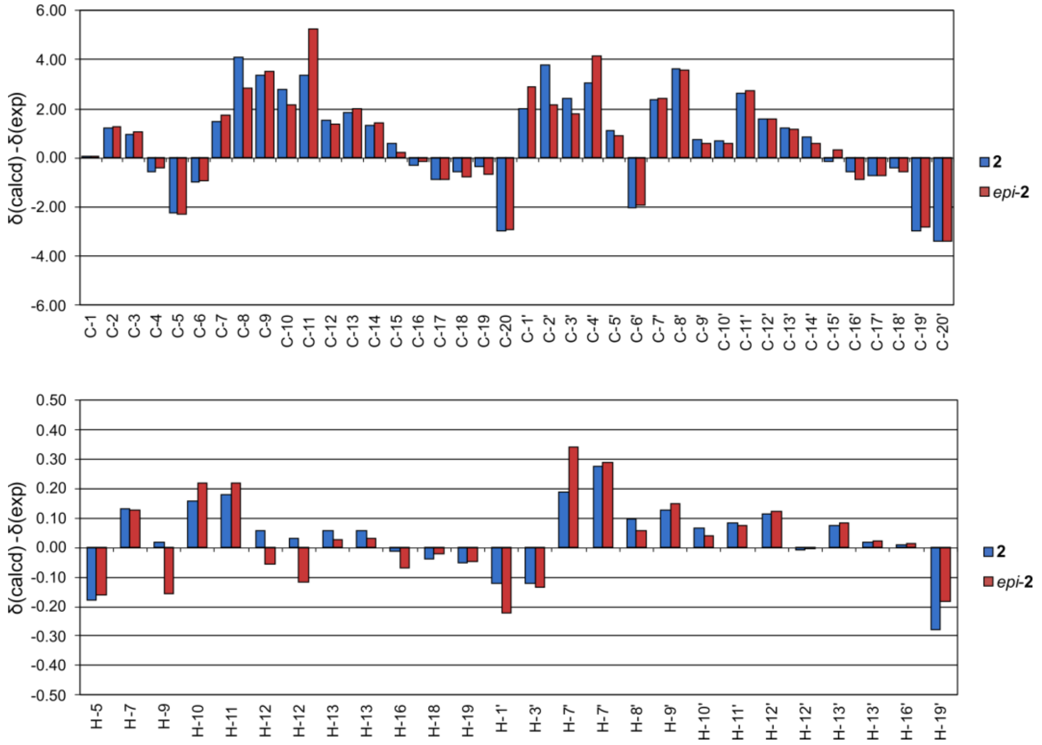

Figure S7. Deviations from experimental values of calculated ^{13}C (top panel) and ^{1}H (bottom panel) NMR chemical shifts of zosterabisphenone B (**2**) and its C-9 epimer *epi*-**2**.

The ECD spectrum of zosterabisphenone B (**2**) was calculated at the ωB97XD/6-31+G(d,p) level, with the PCM continuous model for acetonitrile (Table S15). The predicted spectrum was generated using half-band width $\sigma = 0.30$ eV and the UV correction = +9 nm in the SpecDis program. The predicted ECD spectrum was in good agreement with the experimental spectrum (Figure S27), thus defining the (9*R*,3'*S*) configuration for zosterabisphenone B (**2**).

Experimental section

General methods. Optical rotations were measured on a Jasco P-200 polarimeter at 589 nm using a 10 cm cell. UV spectra were recorded on a Jasco V-530 spectrophotometer. ECD spectra were recorded on a Jasco 715 spectropolarimeter. High-resolution LC-ESI mass experiments was performed on a Thermo LTQ Orbitrap XL mass spectrometer coupled to a Thermo Ultimate 3000 UPLC system. NMR spectra were recorded on a 700 MHz Bruker Avance Neo spectrometer equipped with a cold probe and a BCU-II variable temperature unit. Chemical shifts were referenced to the solvent peaks at δ_H 7.26 and δ_C 77.0 for $CDCl_3$.

Extraction and Isolation. *Zostera marina* L. (Zosteraceae), common eelgrass, is a perennial rhizomateous marine monocot of 30 to 100 cm length. Whole plants of *Zostera marina* L. were collected at the coast close to the Olympiazentrum Schilksee, Kiel, Schleswig-Holstein, Germany in December 2018 (coordinates: N 54°25'39.0", E 10°10'17.5"; alt.: 0 m). The collected plants were unrooted plants freshly washed ashore. A voucher specimen is preserved in the Herbarium of the Institute of Botany, Kiel University (voucher code: YL-20181222A-1; KIEL0005004).

Air-dried, ground whole plants of *Z. marina* (1.75 kg) were extracted with acetone at room temperature (five times, 5.5 L each), yielding 18.3 g of residue after evaporation of the solvent *in vacuo*. The crude extract was subjected to silica gel column chromatography on a 7.5 × 50 cm column, eluted in sequence with hexane/dichloromethane (7:3), hexane/dichloromethane (5:5), dichloromethane/acetone (7:3), dichloromethane/acetone (5:5), and acetone (1 L of each mixture) to yield 24 subfractions.

Fraction N (1.25 g), containing zosterabisphenones A (**1**) and B (**2**), was further separated by medium pressure silica gel column chromatography, performed using a PrepChrom C-700 system (Büchi, Essen, Germany) with a silica gel column (Sepacore Silica 40–63 µm, 80 g, 194 × 31 mm) at a flow rate of 20.0 ml/min. The employed linear gradients were 0 min, hexane; 30min, hexane/CH_2Cl_2 (8:2); 80min, hexane/CH_2Cl_2 (1:1); 81 min, CH_2Cl_2; 120 min, CH_2Cl_2/MeOH (8:2), run time 2 hours, yielding a total of 15 fractions. Fraction N10 (107 mg, eluting with 70% hexane and 30% CH_2Cl_2) contained compound **1**, fraction N11 (114 mg, eluting with 68% hexane and 32% CH_2Cl_2) contained compound **2**.

Fractions N10 and N11 were further purified by Sephadex LH-20 column chromatography (2 × 100 cm) using CH_2Cl_2/acetone (85:15) as eluants. Partially purified compounds **1** (23.5 mg) and **2** (21.2 mg) were finally purified by semi-preparative reversed-phase HPLC using a Waters e2695 instrument and a Nucleodur C_8 column (1 × 25 cm), flow rate 2 ml/min, UV detection at 210 and 254 nm, and mixtures of MeOH and 0.025% formic acid in water as the mobile phase in isocratic separation. Pure zosterabisphenone A (**1**, 4.6 mg) was eluted isocratically with 75% MeOH (Rt 40-46 min) and pure zosterabisphenone B (**2**, 4.2 mg) was eluted with 70% MeOH (Rt 40-46 min).

Zosterabisphenone A (1): $[\alpha]_D^{25}$ –140 (*c* 0.01, ACN); ^{1}H NMR and ^{13}C NMR data, see Table S1; UV/Vis (ACN): λ_{max} (ε) 291 (8500), 196 nm (29300); ECD (ACN): λ_{max} ($\Delta\varepsilon$) 347 (+2.3), 319 (+3.9), 296 (–9.5), 237 (–20.4), 214 (+7.6), 202 nm (–8.1); HRMS (ESI/Orbitrap) m/z: $[M+Na]^+$ calcd for $C_{39}H_{34}O_6Na^+$ 621.2248, found 621.2235; $[M+H-H_2O]^+$ calcd for $C_{39}H_{33}O_5^+$ 581.2323, found 581.2311; $[M+H]^+$ calcd for $C_{39}H_{35}O_6^+$ 599.2428, found 599.2418; $[M+NH_4]^+$ calcd for $C_{39}H_{38}O_6N^+$ 616.2694, found 616.2683; $[M+K]^+$ calcd for $C_{39}H_{34}O_6K^+$ 637.1987, found 637.1974; $[M+HCOO+Ca]^+$ calcd for $C_{40}H_{35}O_8Ca^+$ 683.1952, found 683.1940.

Zosterabisphenone B (2): $[\alpha]_D^{25}$ –55 (*c* 0.01, ACN); UV/Vis (ACN): λ_{max} (ε) 294 (10400), 230 (30000), 200 nm (40700); ECD (ACN): λ_{max} ($\Delta\varepsilon$) 314 (+9.9), 280 (–17.4), 259 (–17.2), 240 (+12.2), 218 (–35.8), 192 nm (+26.2); ^{1}H NMR and ^{13}C NMR data, see Table S3; HRMS (ESI/Orbitrap) m/z: $[M+H]^+$calcd for $C_{40}H_{37}O_8^+$ 645.2483, found 645.2470; $[M+Na]^+$ calcd for $C_{40}H_{36}O_8Na^+$ 667.2302, found 667.2289; $[M+K]^+$ calcd for $C_{40}H_{36}O_8K^+$ 683.2042, found 683.2029; $[M+HCOO+Ca]^+$ calcd for $C_{41}H_{37}O_{10}Ca^+$ 729.2007, found 729.1996.

General computational methods. Conformational search was performed using molecular dynamics (MD) with the INSIGHT II/Discover package (BIOVIA, 5005 Wateridge Vista Drive, San Diego, CA 92121, USA). All the MD simulations were performed at 2000 K to allow possible slow conformational changes to occur in the short duration of the simulation,[6] constraining the geometry of double bonds to prevent *cis*/*trans* isomerization. The effect of the solvent (chloroform) was approximated by using a dielectric constant of 4.81. The search protocol involved a 10-ns MD simulation in the CFF91 force field. The coordinates were saved every 50 ps and subsequently minimized in the same force field, giving 200 minimized structures, which were used as input for the subsequent quantum-mechanical calculations.

Density functional theory (DFT) calculations were performed using the program Gaussian 16 (Revision C.01, Gaussian Inc, Wallingford CT, USA), using the B3LYP/6-31+G(d,p) level of theory for structure optimization, the Gauge Invariant Atomic Orbitals (GIAO) method[8] at the PBE0/6-311+G(2d,p) level of theory and the PCM solvent model for prediction of NMR chemical shifts, and the time-dependent DFT (TDDFT) method at the ωB97XD/6-31+G(d,p) level of theory and the PCM solvent model for ECD prediction. Proton-proton NMR scalar couplings were calculated according to the suggestions of Bally and Rablen:[13] calculations were

performed at the B3LYP/6-31G(d,p) level of theory in vacuo, and only the Fermi contact terms were calculated, which were then scaled by a factor of 0.9117.

The NMR isotopic shielding computed for each H or C nucleus was converted into a chemical shift using the scaling factors proposed by the Tantillo group[10] for the level of theory used (^{1}H: slope −1.0958, intercept 31.7532; ^{13}C: slope −1.0533, intercept: 187.3123). When more than one conformer were significantly populated, the weighted mean of the chemical shifts and scalar couplings of individual conformers was calculated using Boltzmann statistics (T = 253 K).

The predicted ECD curves were obtained using the program SpecDis v. 1.71,[15] adjusting the parameters σ and UV shift for the best fit between the predicted and experimental spectra. When more than one conformer were significantly populated, the weighted mean of the ECD curves of individual conformers was calculated using Boltzmann statistics (T = 298 K).

Cell cultures. The human adenocarcinoma colon cancer cell line (HCT116) and the human liver colon cancer cell line (Hep G2) were purchased from ATCC (Manassas, VA, USA). HCT116 and Hep G2 cells were cultured in McCoy's 5A medium and Minimal Essential Medium (Euroclone, MI, Italy), respectively. Both cell lines were supplemented with 10% Fetal Bovine Serum (FBS) plus 2mM glutamine and 100UI penicillin and 100 μg/ml streptomycin. Cells were maintained under standard cell culture conditions at 37 °C in a humidified atmosphere of 5% CO_2 in air.

Cell viability assay (MTT assay). Compounds **1** and **2** were dissolved in pure EtOH (Sigma Aldrich, St Louis, MO, USA). Final EtOH concentration in cell culture medium never exceeded 0.1% (v/v) and equal amounts of the solvent were added to control cells (untreated cells). Cell viability was assessed by using the tetrazolium dye [3-(4,5-dimethylthiazol-2-yl)-2,5-diphenyltetrazolium bromide] (MTT) assay as previously described.[19] Briefly, HCT116 (5×10^3) cells or Hep G2 cells (1×10^4) cells were seeded in culture media which contained 10% FBS on 96-well plates and left to adhere for 24 h. After that, the cells were incubated with increasing concentrations (0-10 μM) of zosterabisphenones in cell culture medium containing 1% FBS for 24 or 48 h. Control cells received only the vehicle (EtOH). After the indicated incubation period times, MTT (0.25 mg/mL) was added into each well according to the manufacturer's instructions for 1 h at 37 °C. Thereafter, 100 μL of DMSO was added into each well and the absorbance (OD) was measured at 570 nm using a microplate reader (BioTek™ Cytation™ 3, Winooski, VT, USA).

Statistical analysis. Statistical analysis, performed using GraphPad Prism 7.0 (GraphPad software, San Diego, CA, USA), was determined by two-way analysis of variance (ANOVA) followed by a Turkey-Kramer multiple comparisons test. The concentration of compounds that produced 50% inhibition of cell viability (EC_{50}) was calculated by nonlinear regression analysis using the equation for a sigmoid concentration-response curve. $P < 0.05$ was considered significant. Data were expressed as the mean ± mean standard error (SEM) of n experiments.

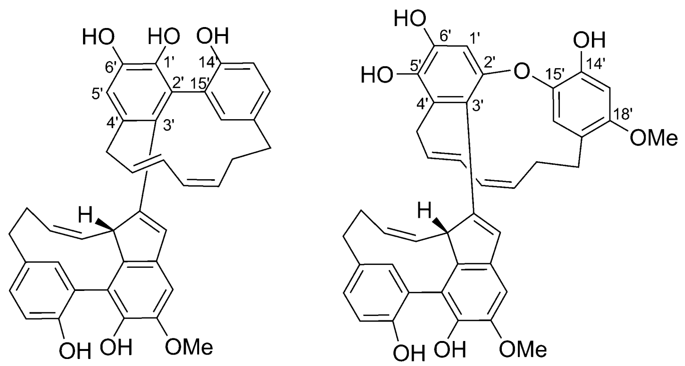

hypothetical aromatic tautomer of zosterabisphenone A (**1a**)

hypothetical aromatic tautomer of of zosterabisphenone B (**2a**)

Chart S1

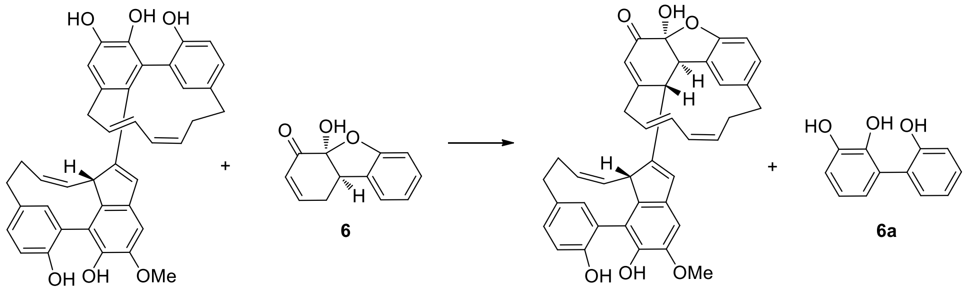

	1a	6	1	6a
E(Harthree)	-1957.980709	-688.990641	-1957.993384	-689.017902
E(kcal/mol)	-1228638.77	-432343.69	-1228646.72	-432360.80
ΔE (kcal/mol)				−25.06

Scheme S1. The isodesmic reaction used to evaluate the release of steric strain in zosterabisphenone A (**1**) compared to its aromatic tautomer **1a**. All energies were calculated, after optimization, at the B3LYP/6-31+G(d,p) level; starting geometries of compounds **6** and **6a** were taken from compounds **1** and **1a**, respectively. The release of steric strains was calculated as $\Delta E = E_{products} - E_{reaactants}$ and found to be 25.06 kcal/mol.

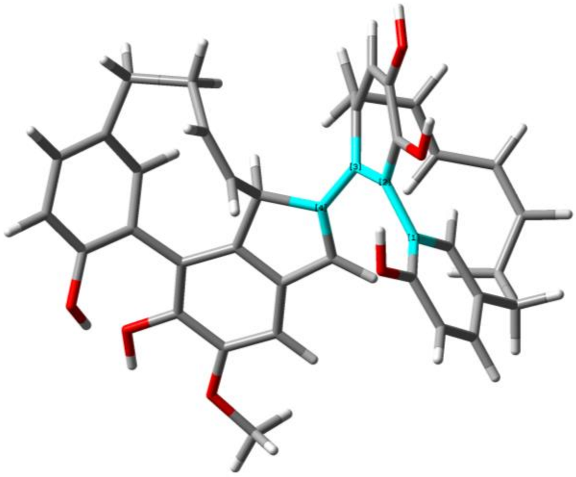

Figure S8. Minimum energy conformation of the aromatic tautomer **1a** of zosterabisphenone A. The energy of the aromatic tautomer calculated at the B3LYP/6-31+G(d,p) level was higher by 7.95 kcal/mol than that of the keto tautomer. The two ortho bonds at C-2' and C-3' are pushed out of planarity by steric hindrance up to 40.8° (the value of the dihedral angle C-15'/C-2'/C-3'/C-8, highlighted in the figure).

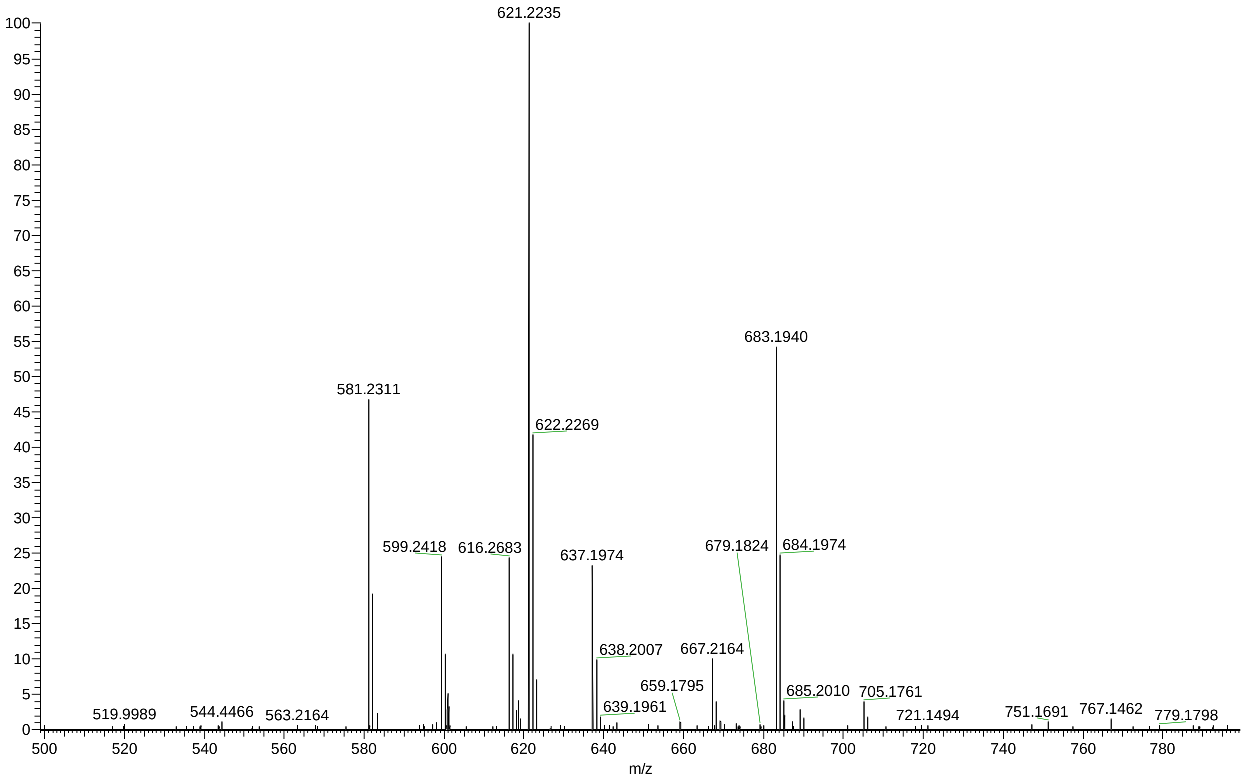

Figure S9. High-resolution ESI mass spectrum of zosterabisphenone A (**1**) (positive ion mode, MeOH/0.1% aqueous HCOOH).

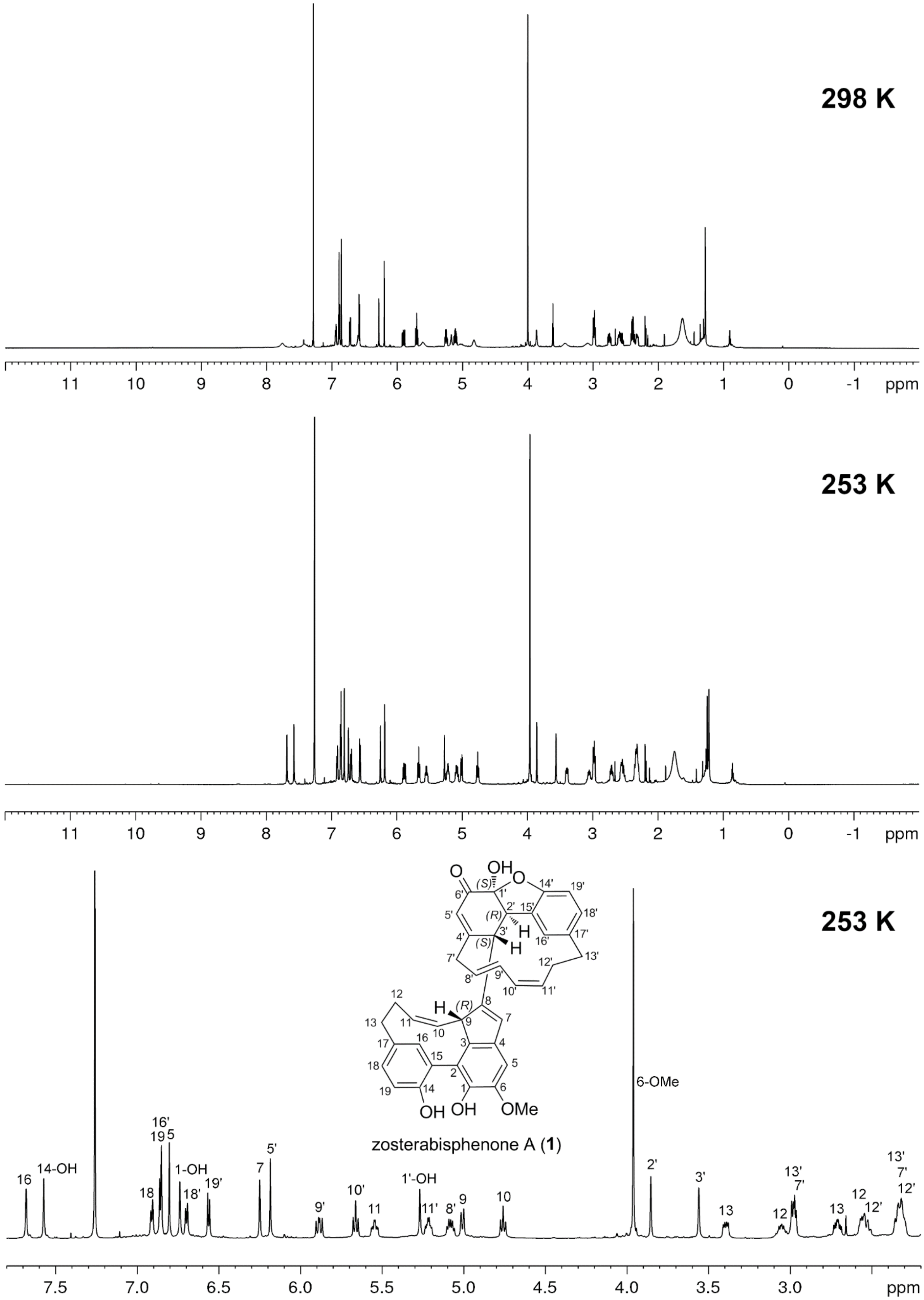

Figure S10. ^{1}H NMR spectrum of zosterabisphenone A (**1**) recorded at 298 K and 253 K (700 MHz, $CDCl_3$)

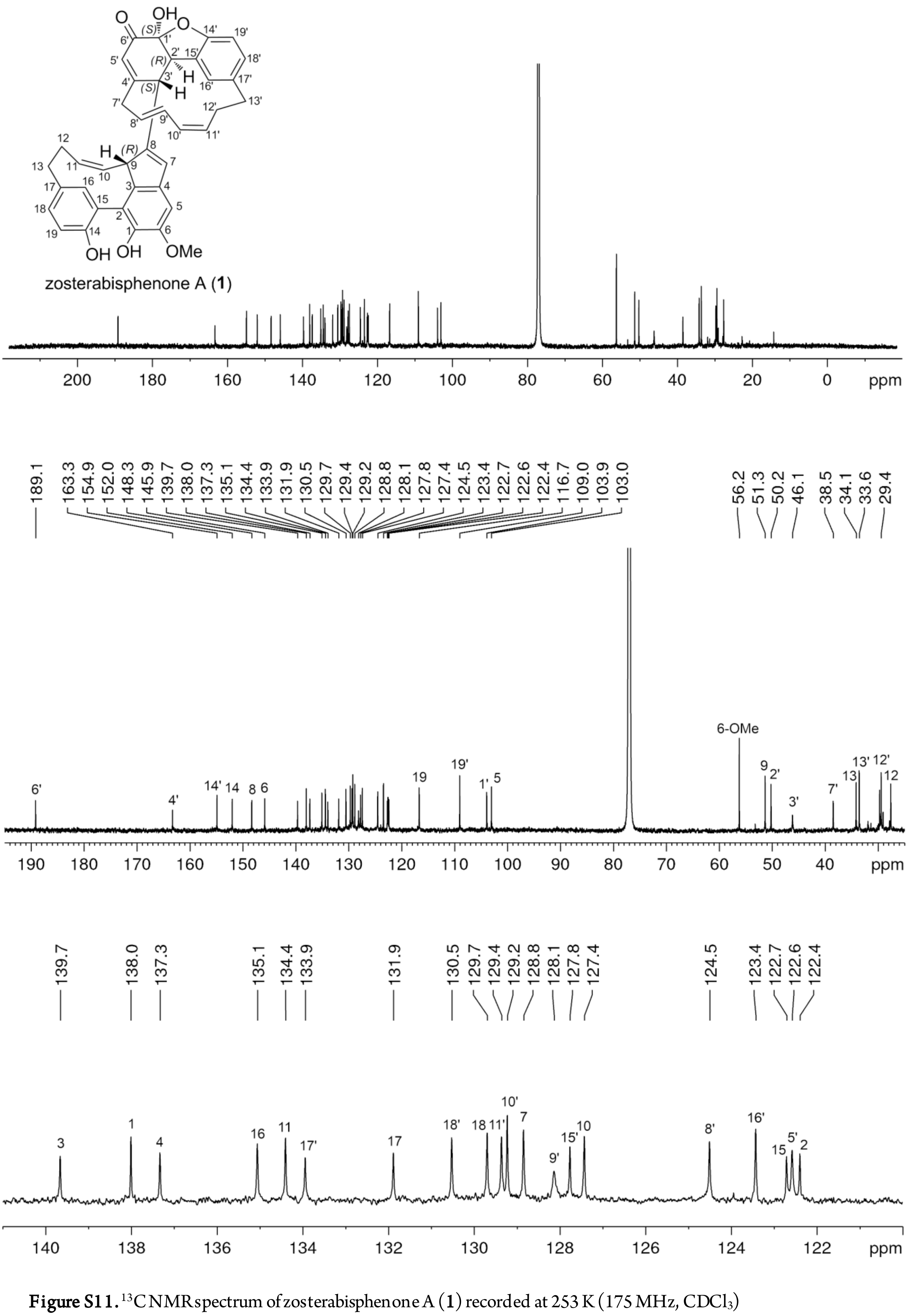

Figure S11. ^{13}C NMR spectrum of zosterabisphenone A (**1**) recorded at 253 K (175 MHz, $CDCl_3$)

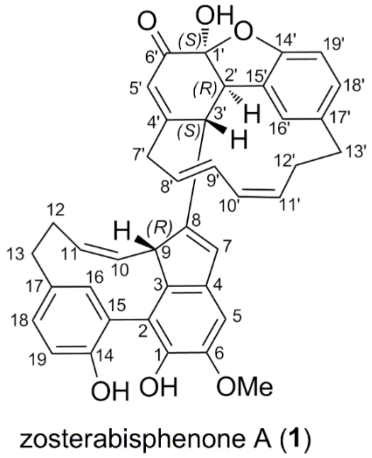

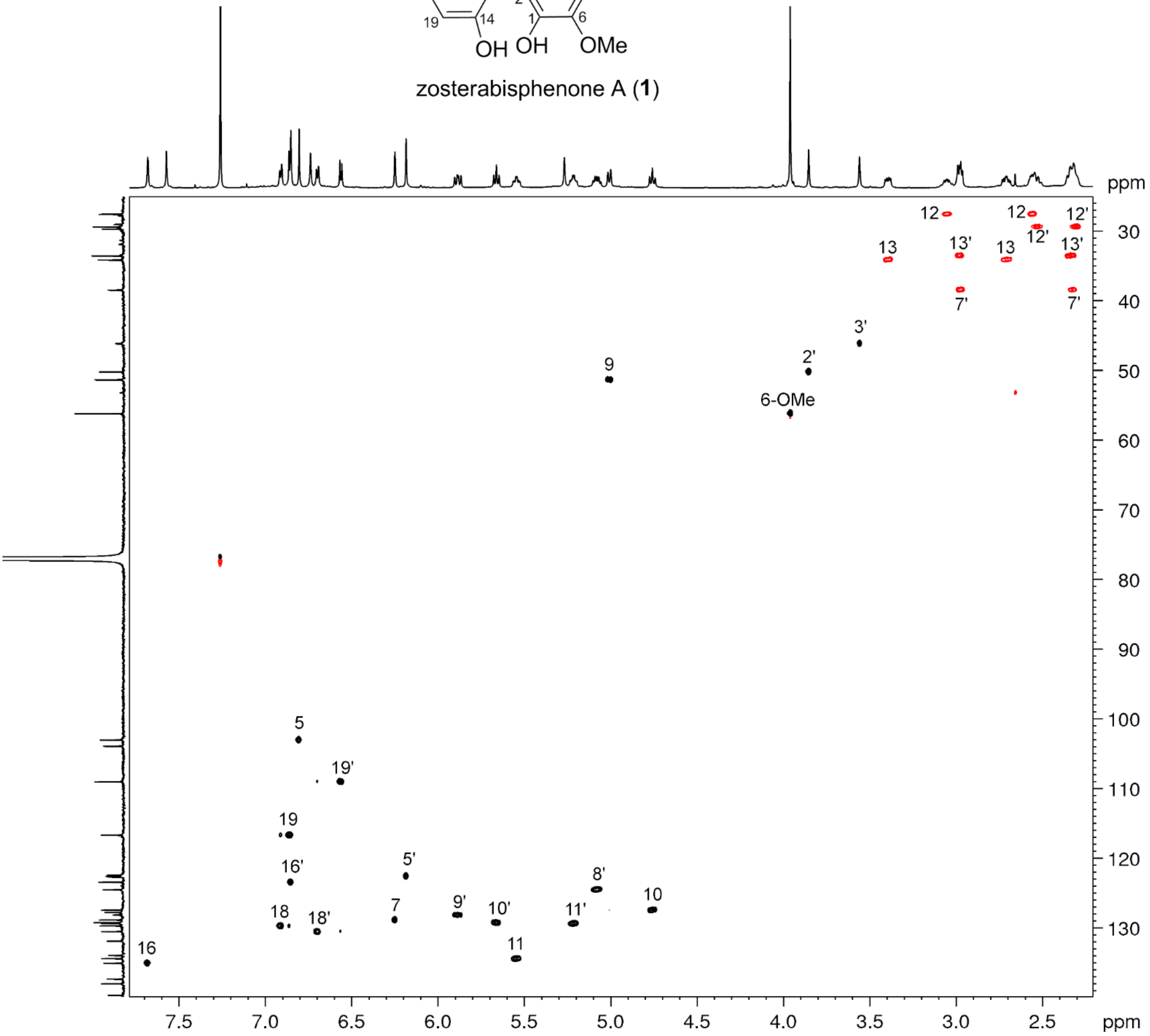

Figure S12. HSQC spectrum of zosterabisphenone A (**1**) recorded at 253 K (700 MHz, $CDCl_3$)

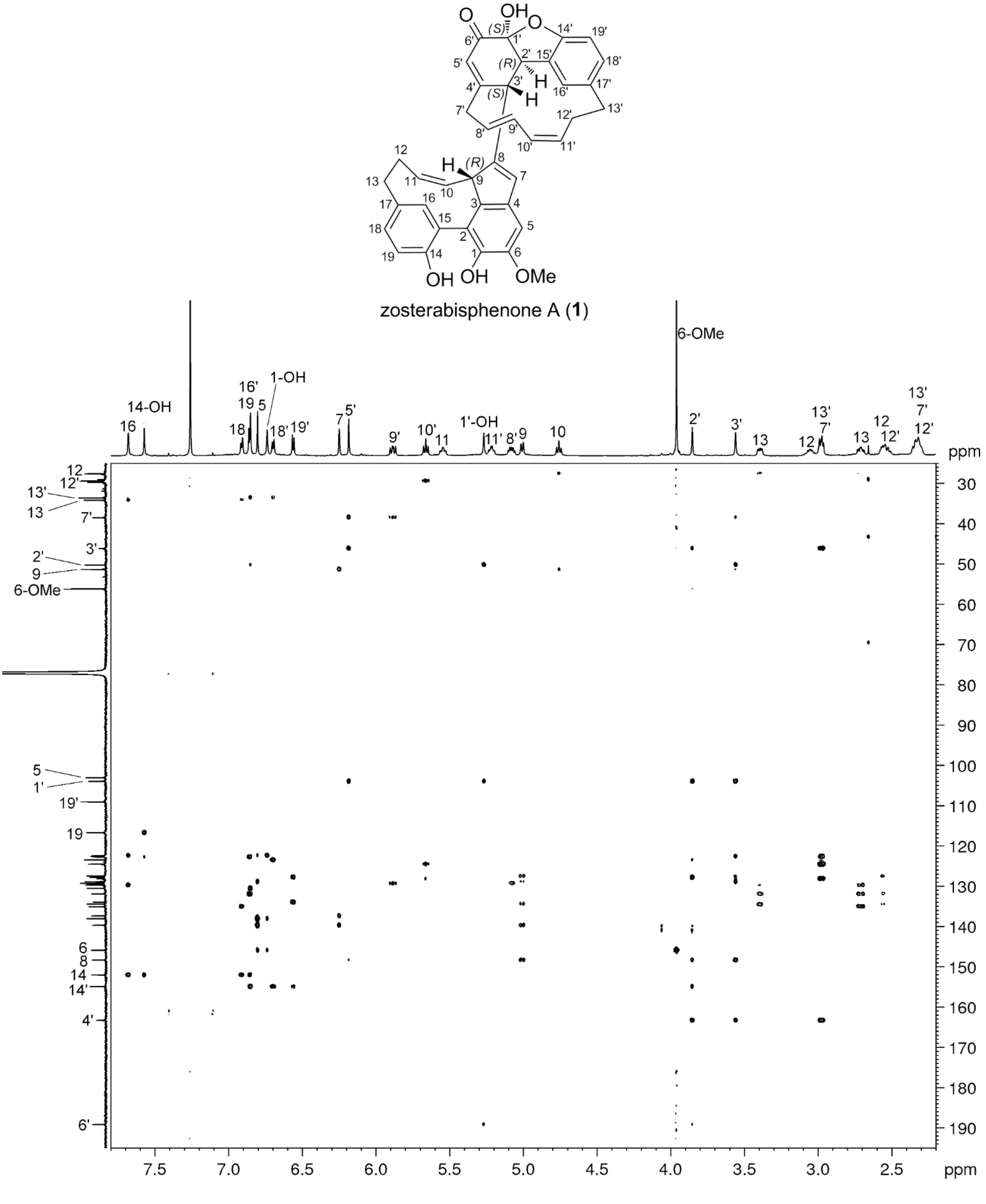

Figure S13. HMBC spectrum of zosterabisphenone A (**1**) recorded at 253 K (700 MHz, $CDCl_3$)

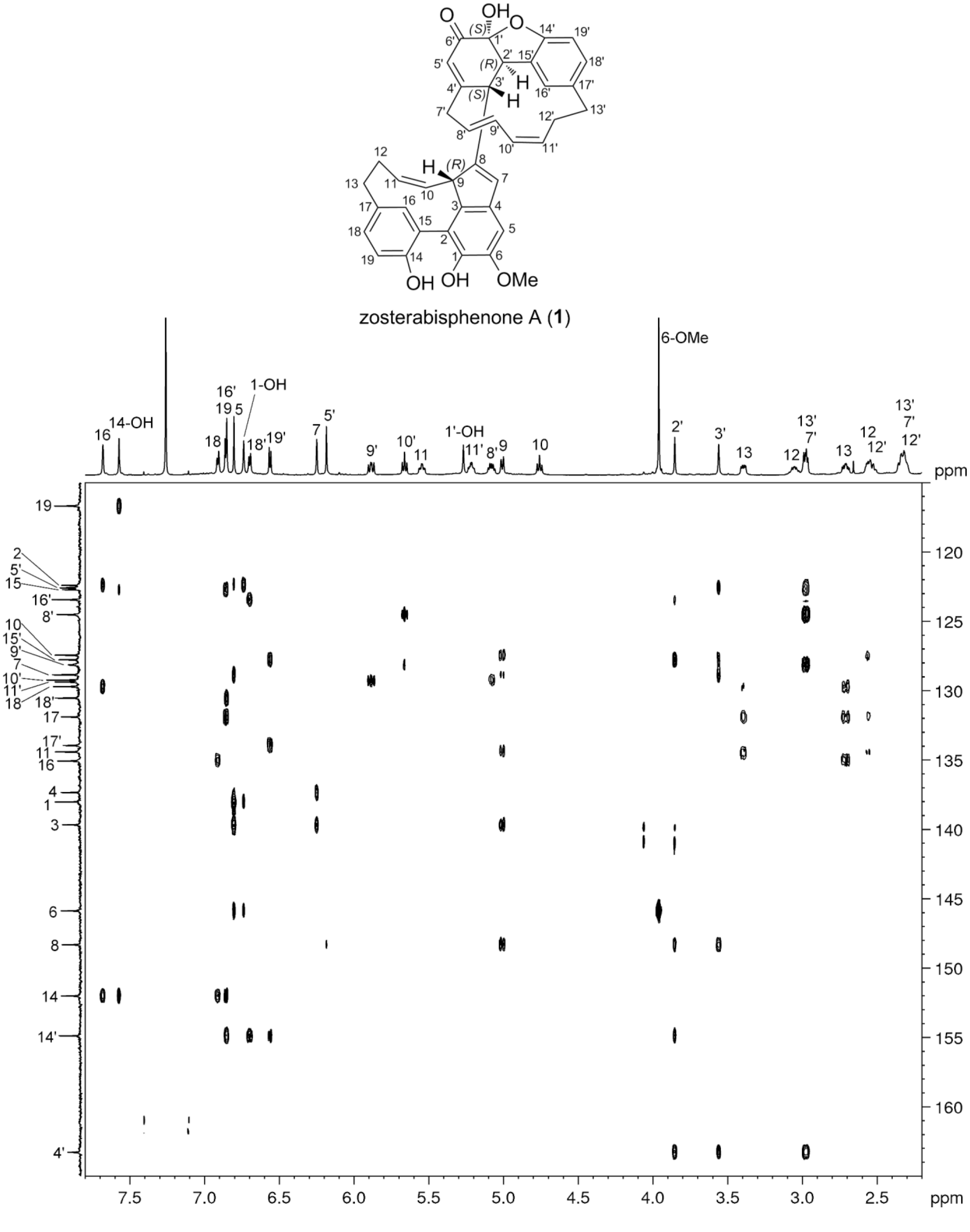

Figure S14. Expansion of the HMBC spectrum of zosterabisphenone A (**1**) recorded at 253 K (700 MHz, $CDCl_3$)

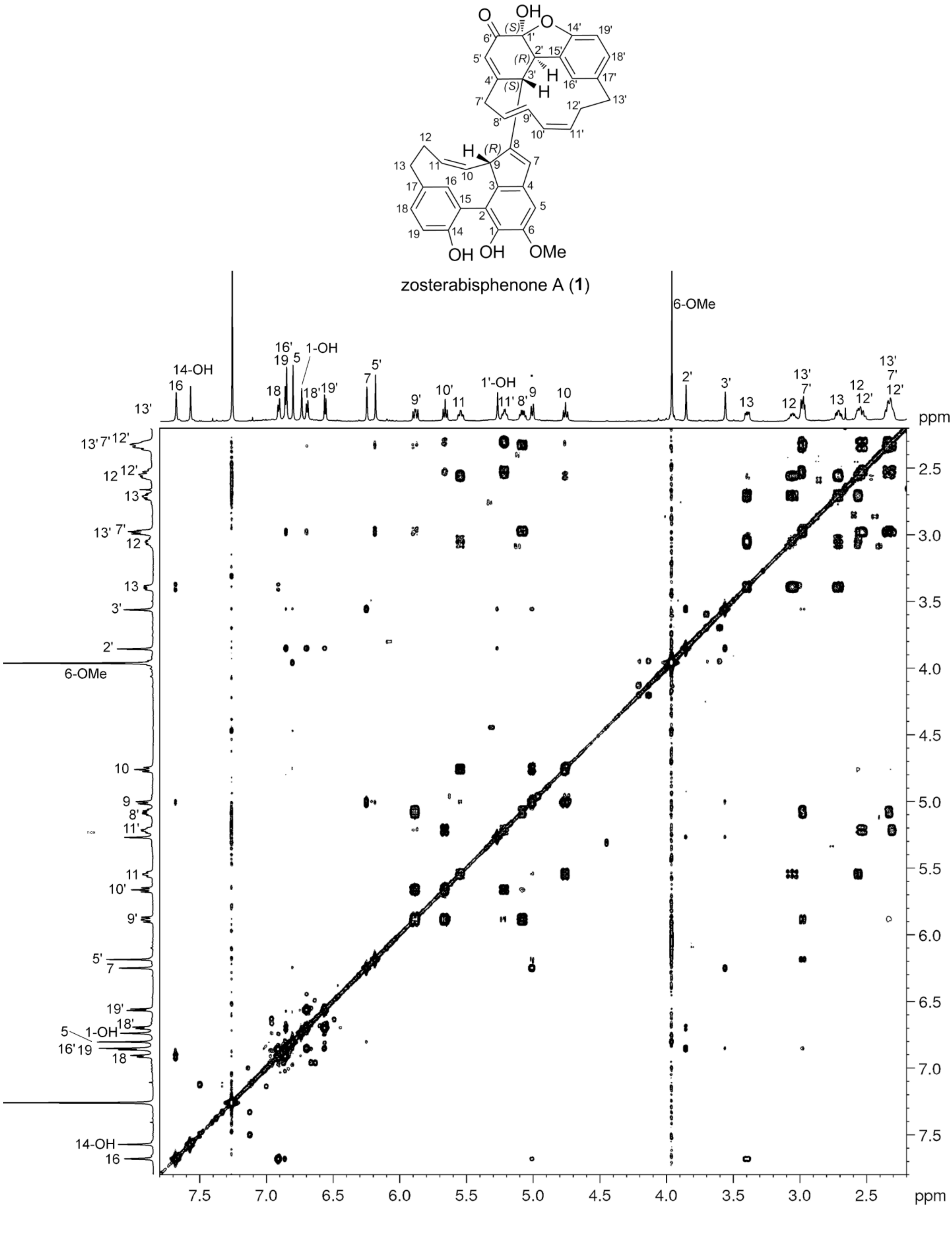

Figure S15. COSY spectrum of zosterabisphenone A (**1**) recorded at 253 K (700 MHz, $CDCl_3$)

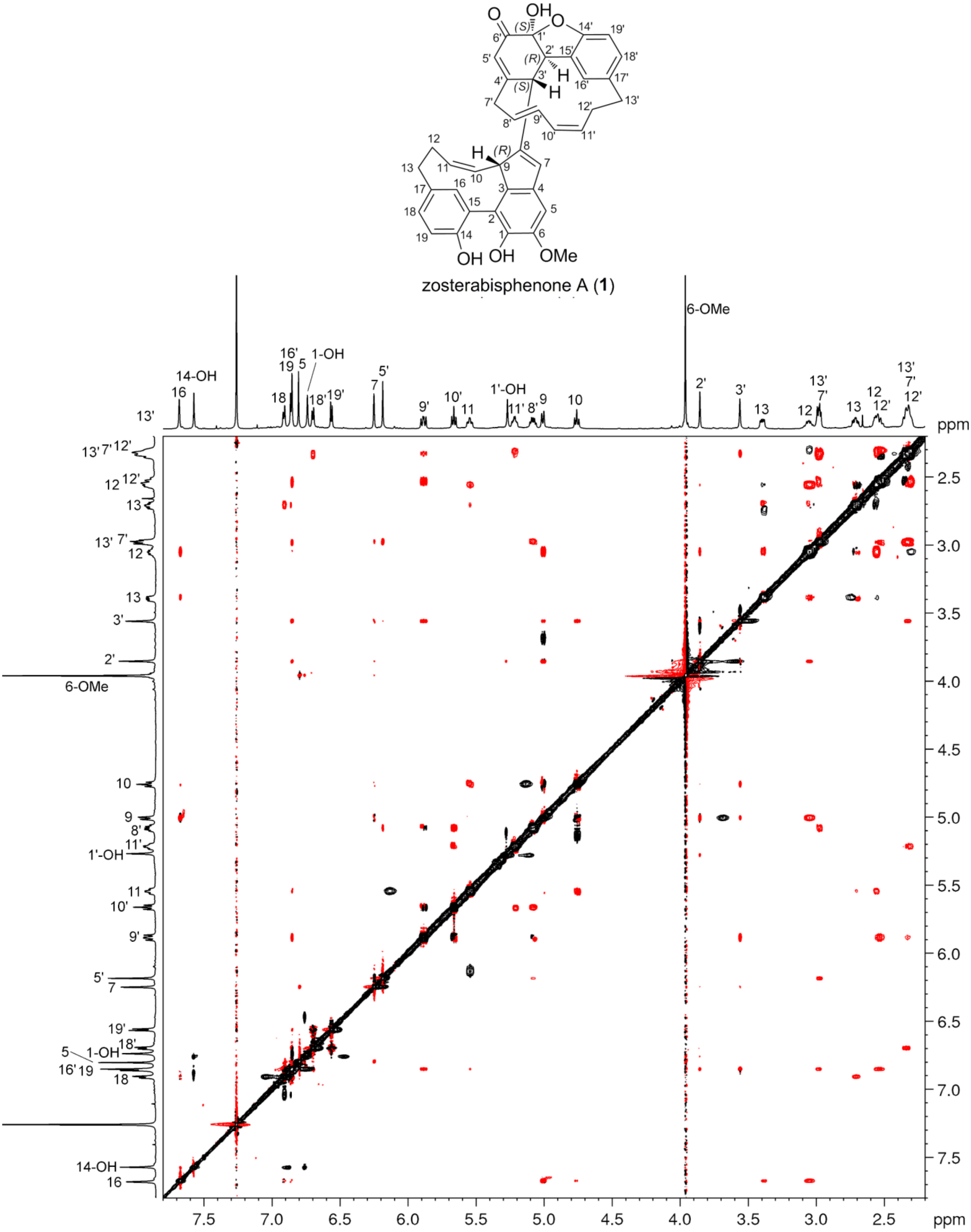

Figure S16. ROESY spectrum of zosterabisphenone A (**1**) recorded at 253 K (700 MHz, $CDCl_3$)

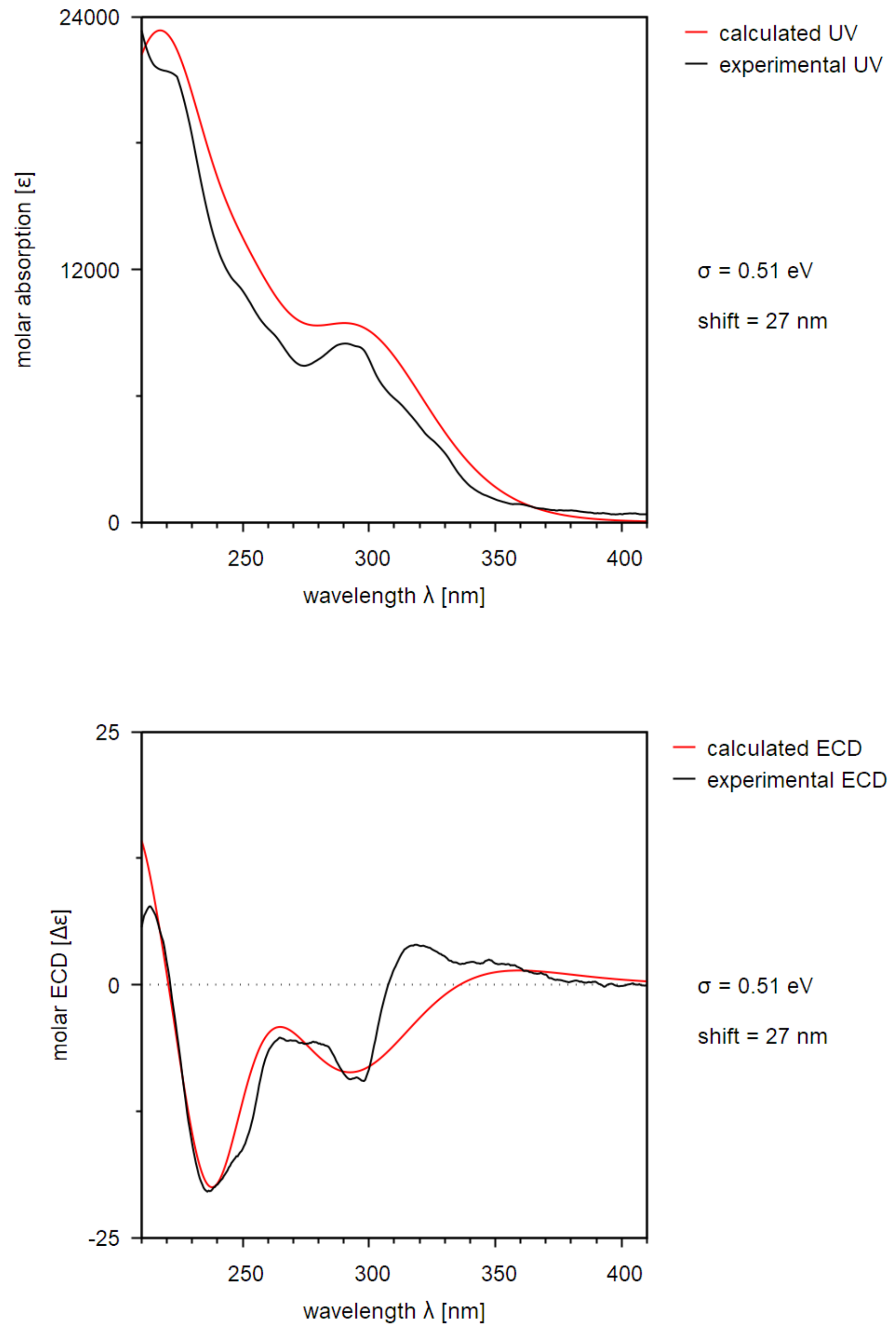

Figure S17. Top panel: calculated and experimental UV spectra of zosterabisphenone A (**1**) in acetonitrile; bottom panel: calculated and experimental ECD spectra of zosterabisphenone A (**1**) in acetonitrile.

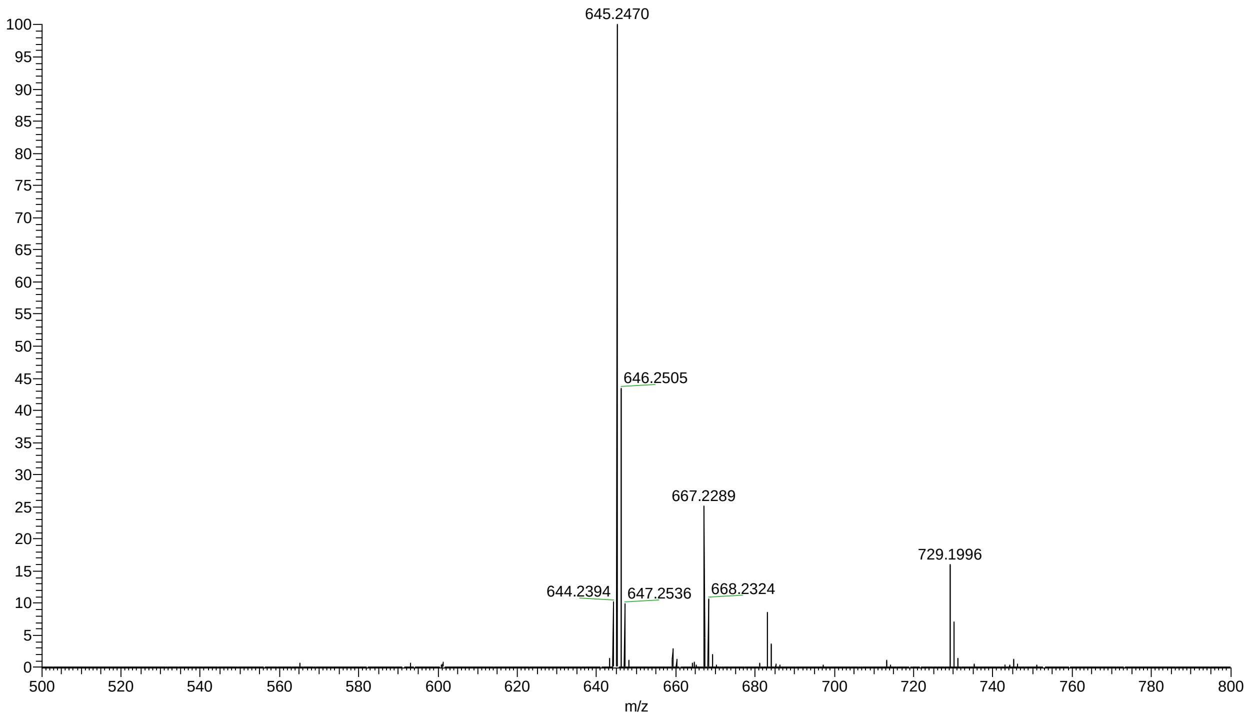

Figure S18. High-resolution ESI mass spectrum of zosterabisphenone B (**2**) (positive ion mode, MeOH/0.1% aqueous HCOOH).

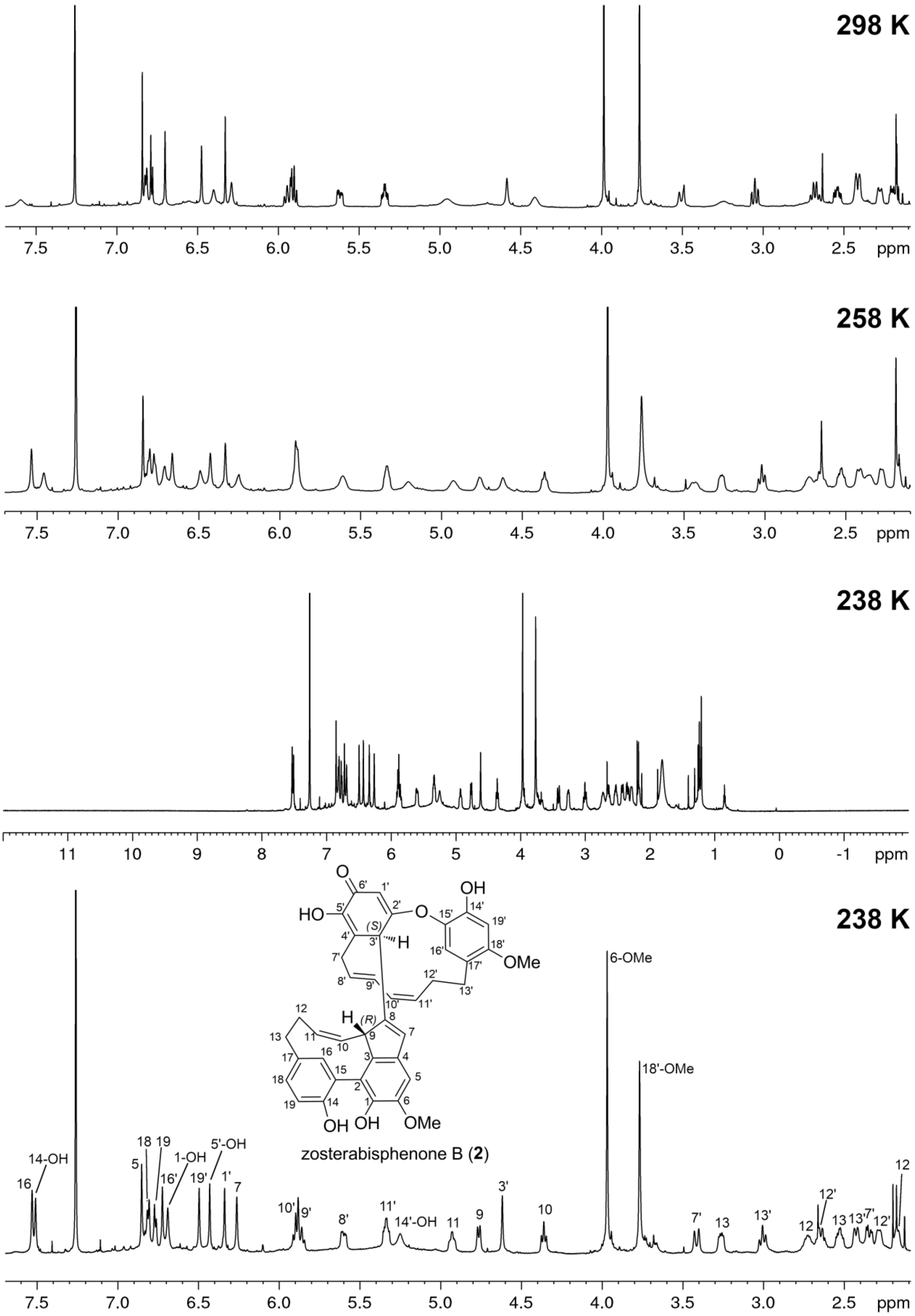

Figure S19. ^{1}H NMR spectrum of zosterabisphenone B (**2**) recorded at 298 K, 258 K and 238 K (700 MHz, $CDCl_3$)

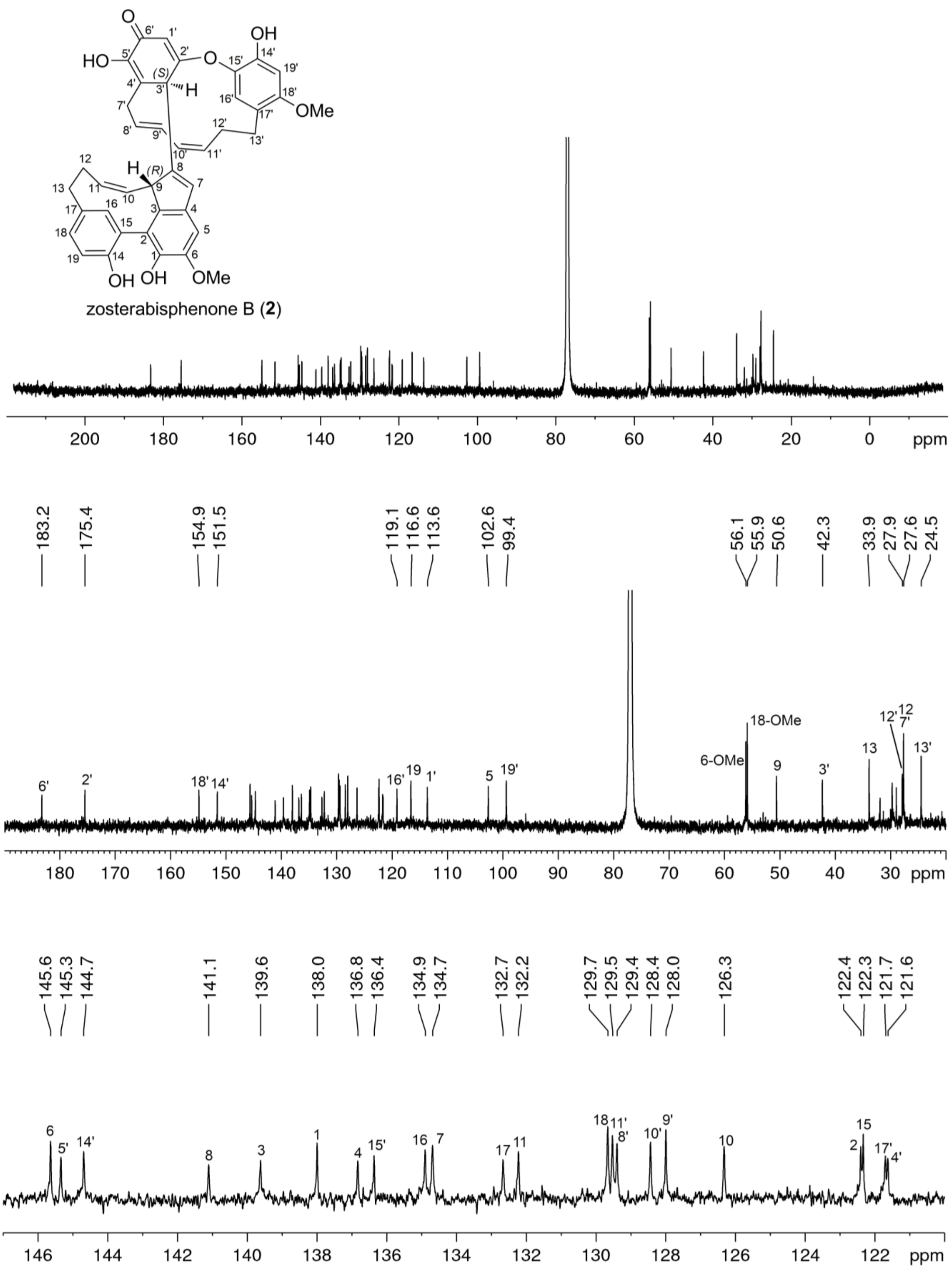

Figure S20. ^{13}C NMR spectrum of zosterabisphenone B (**2**) recorded at 238 K (700 MHz, $CDCl_3$)

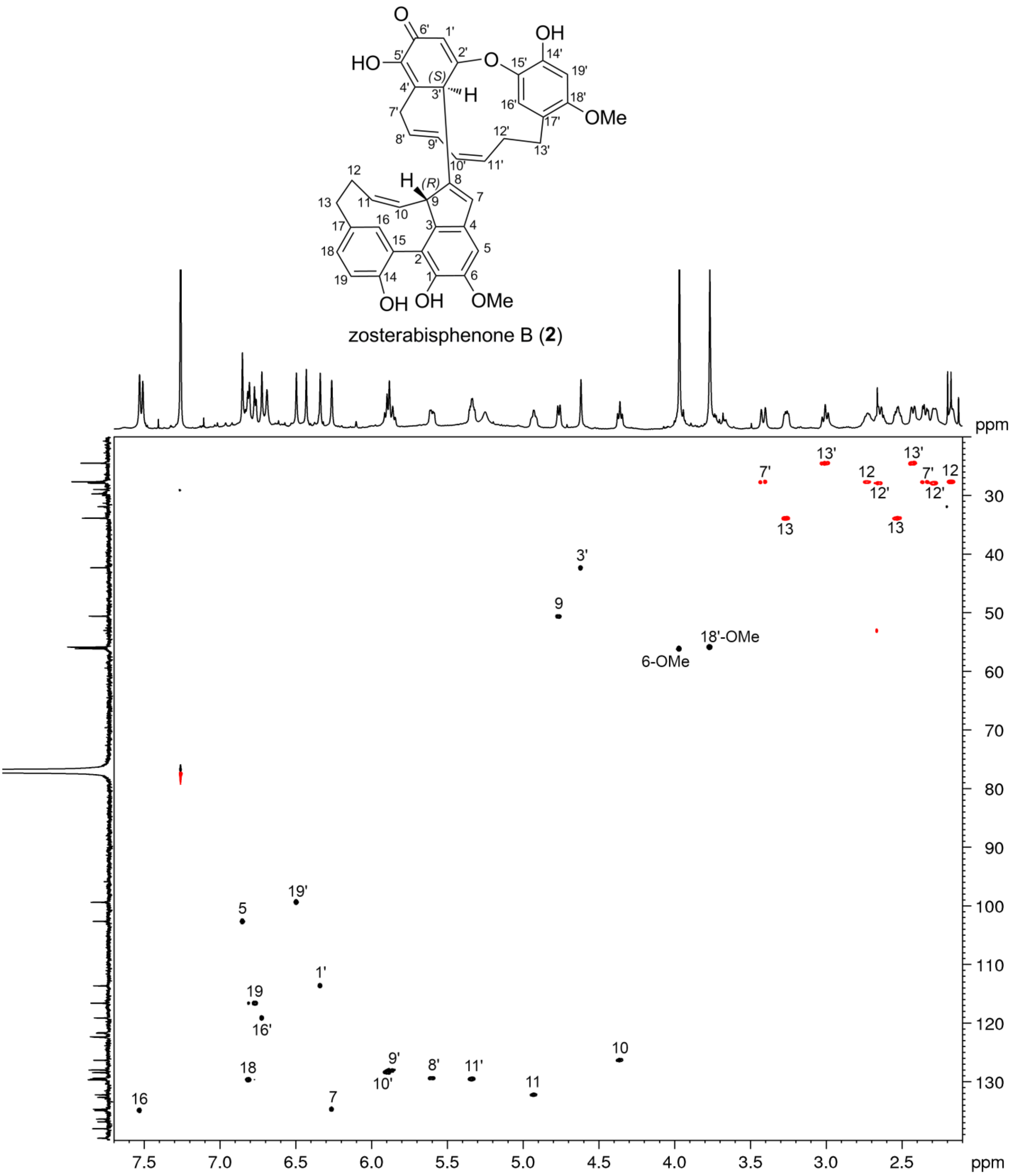

Figure S21. HSQC spectrum of zosterabisphenone B (**2**) recorded at 238 K (700 MHz, $CDCl_3$)

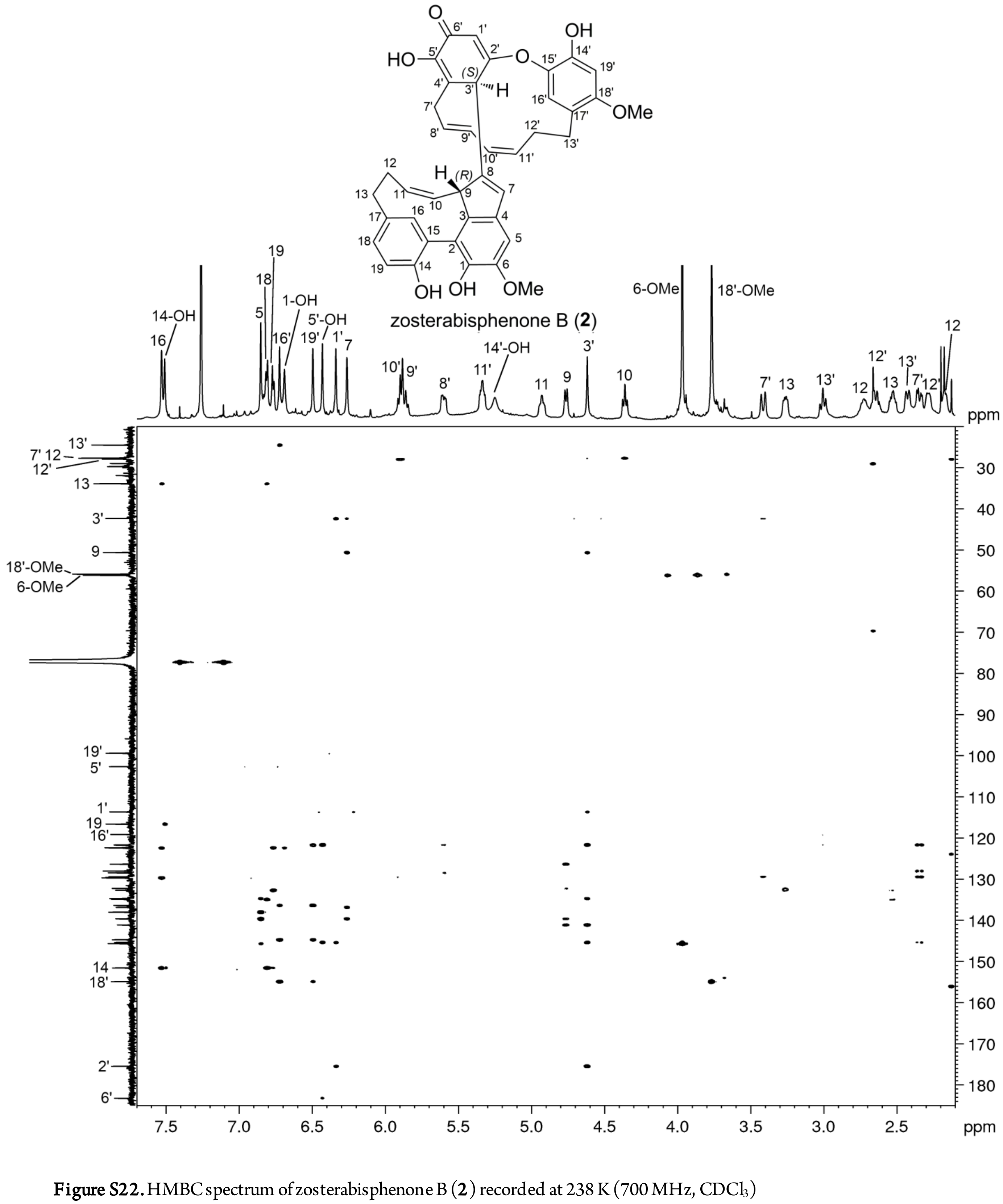

Figure S22. HMBC spectrum of zosterabisphenone B (**2**) recorded at 238 K (700 MHz, $CDCl_3$)

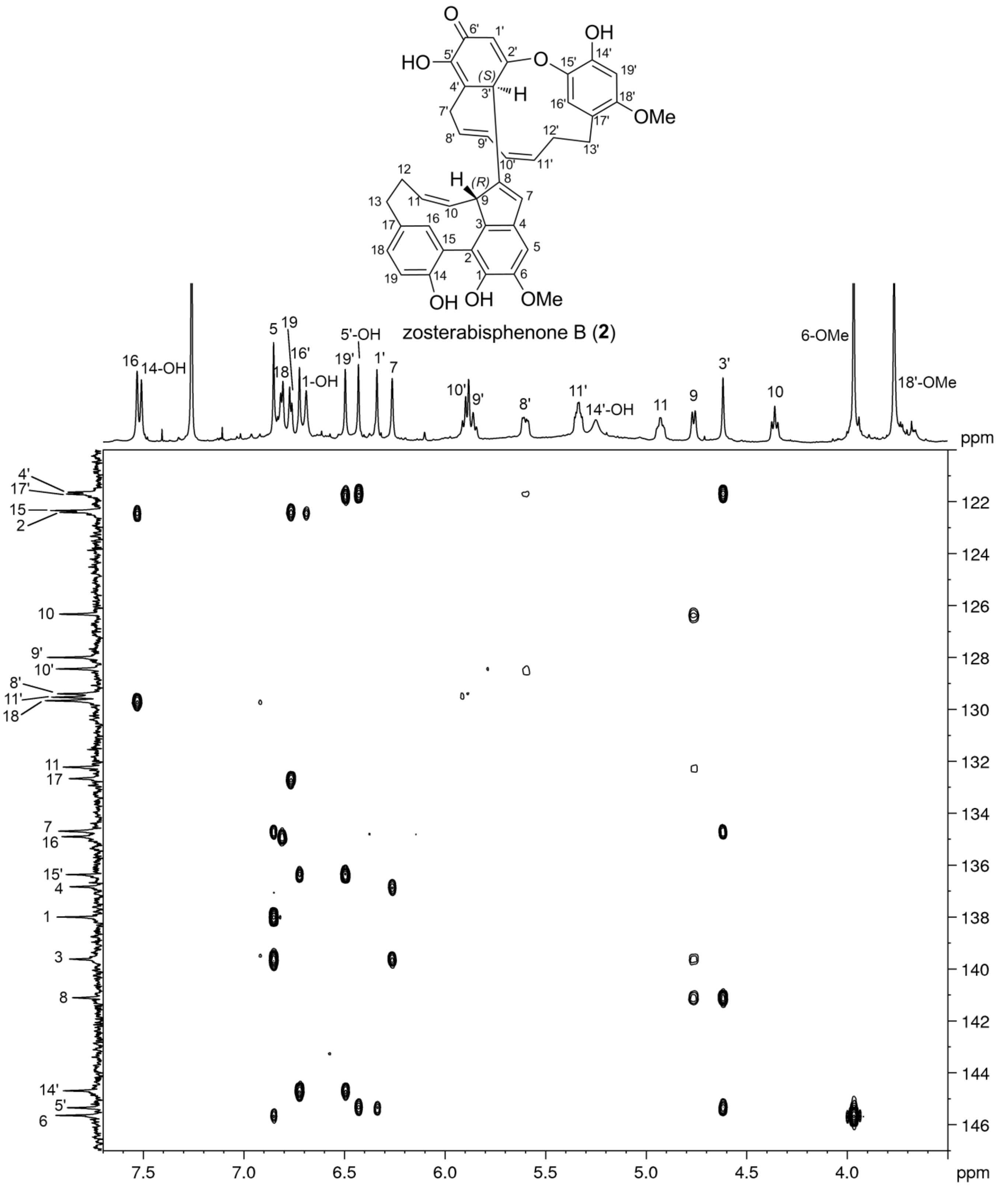

Figure S23. Expansion of the HMBC spectrum of zosterabisphenone B (**2**) recorded at 238 K (700 MHz, $CDCl_3$)

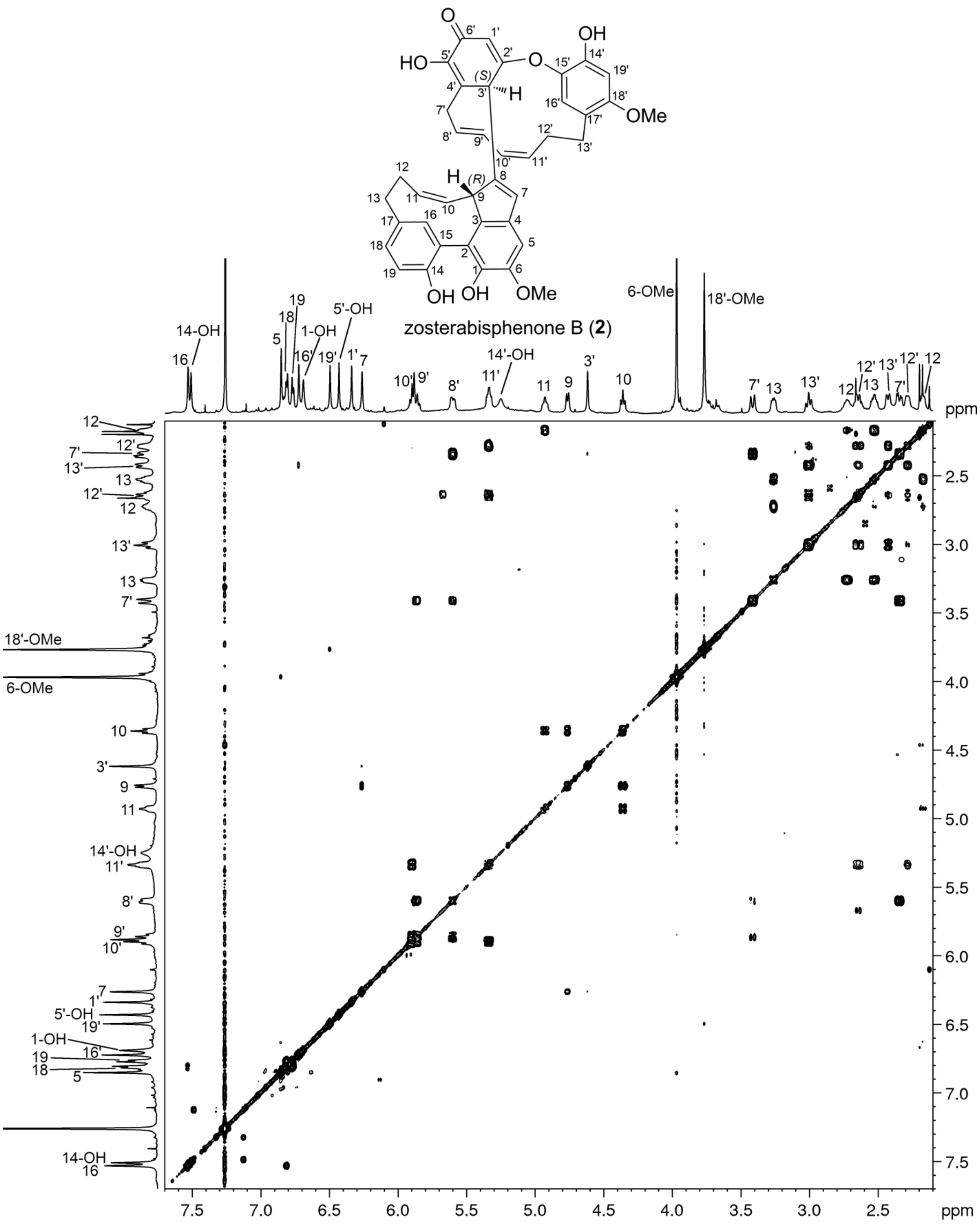

Figure S24. Expansion of the HMBC spectrum of zosterabisphenone B (**2**) recorded at 238 K (700 MHz, $CDCl_3$)

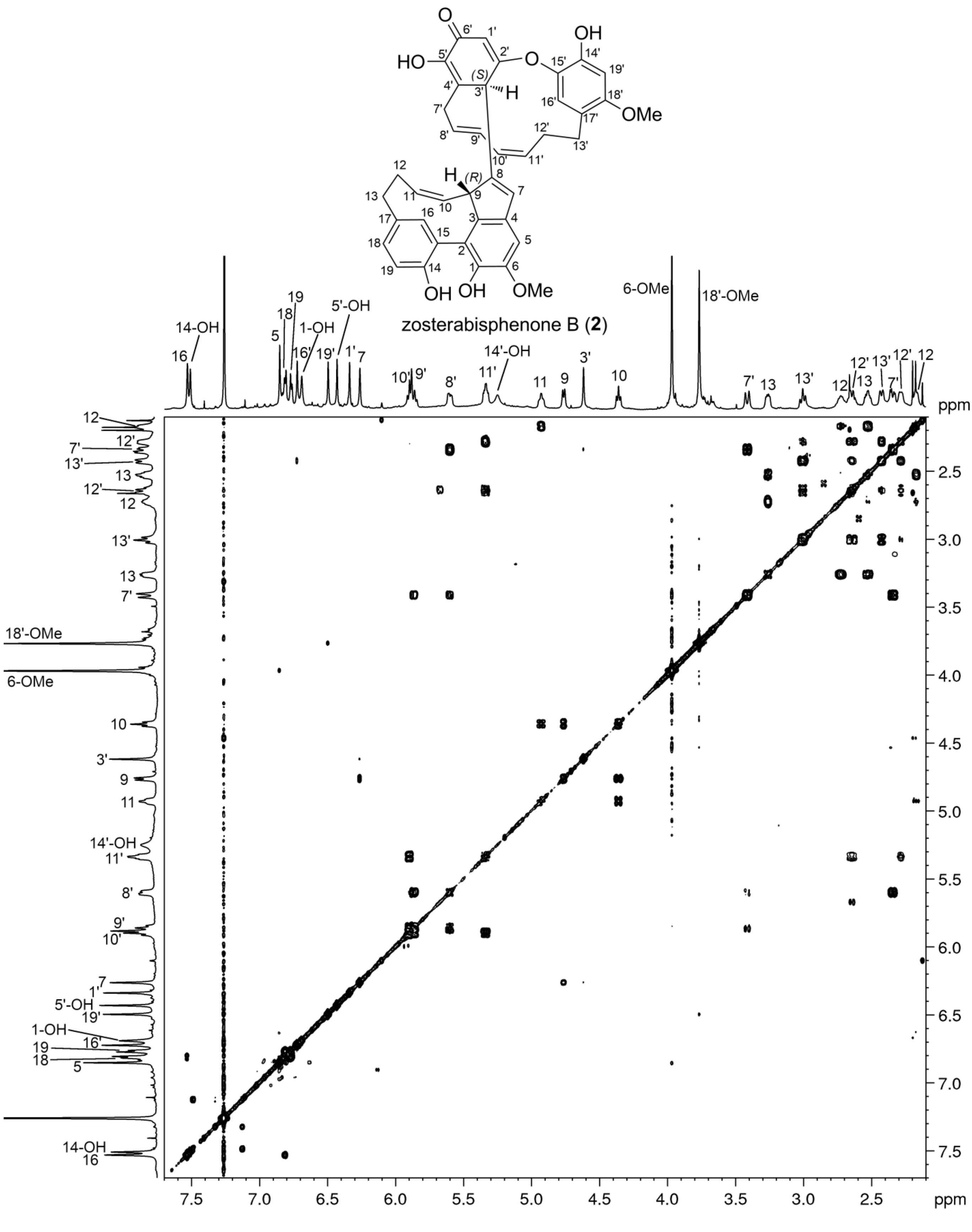

Figure S25. COSY spectrum of zosterabisphenone B (**2**) recorded at 238 K (700 MHz, $CDCl_3$)

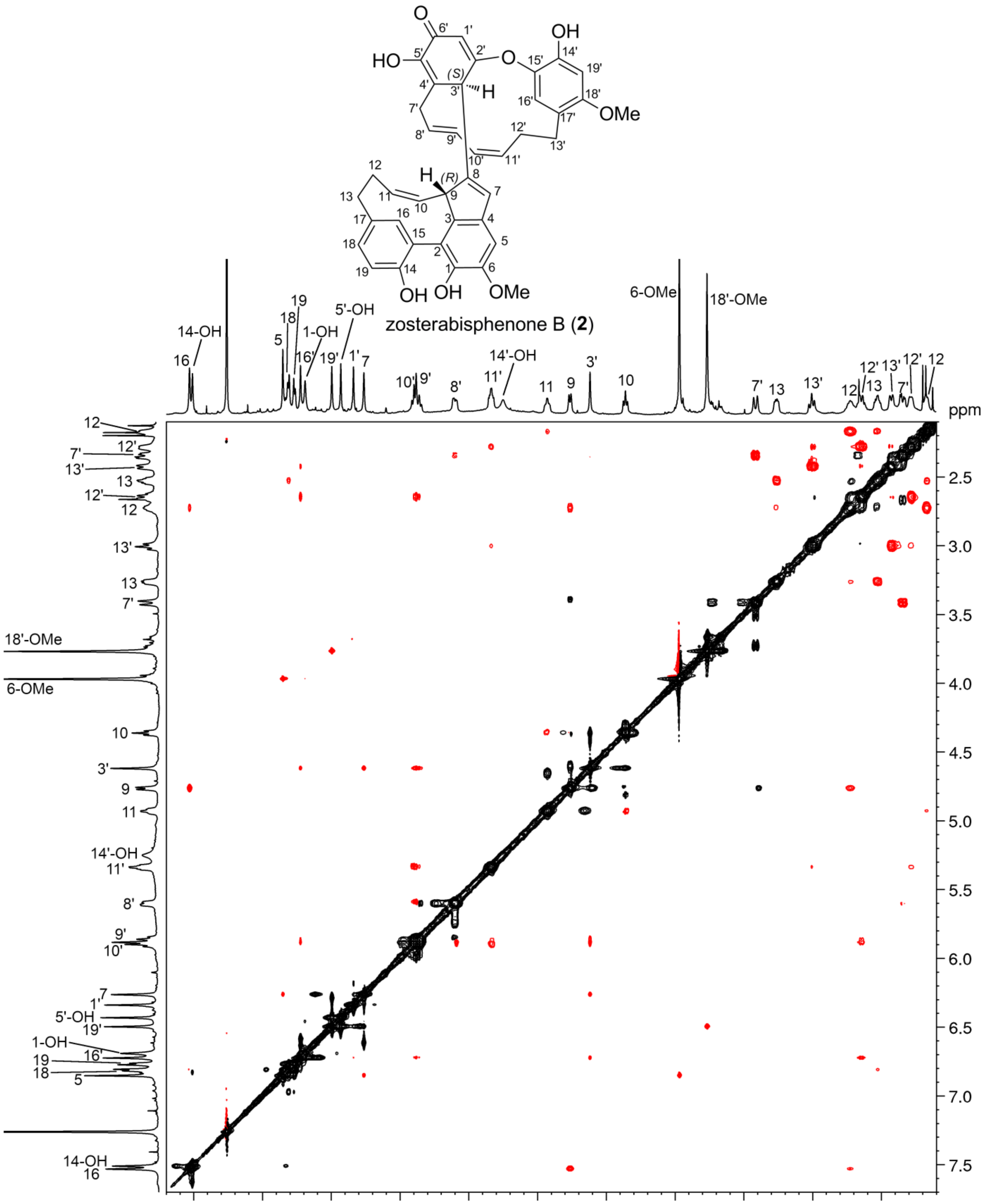

Figure S26. ROESY spectrum of zosterabisphenone B (**2**) recorded at 238 K (700 MHz, $CDCl_3$)

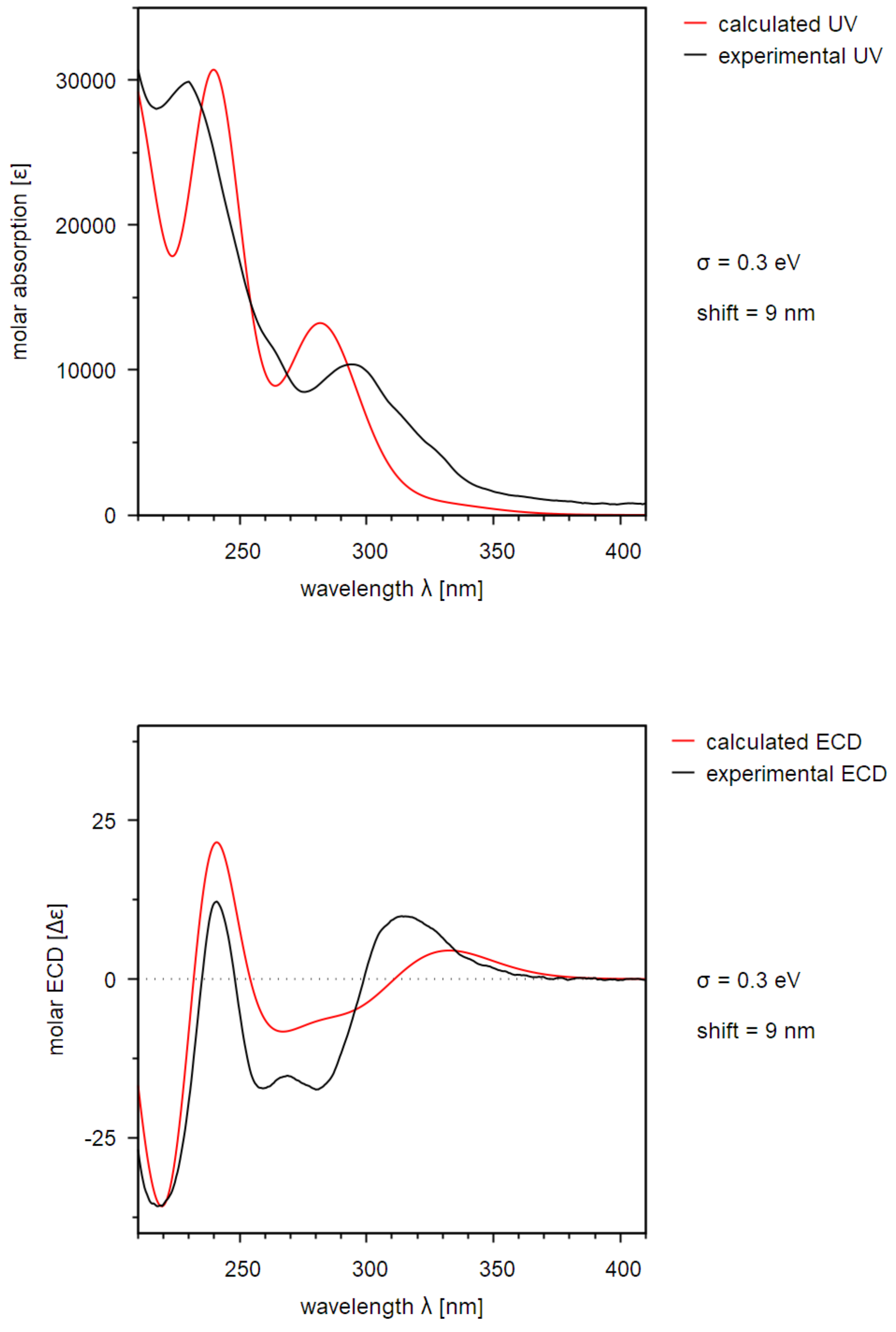

Figure S27. Top panel: calculated and experimental UV spectra of zosterabisphenone B (**2**) in acetonitrile; bottom panel: calculated and experimental ECD spectra of zosterabisphenone B (**2**) in acetonitrile.

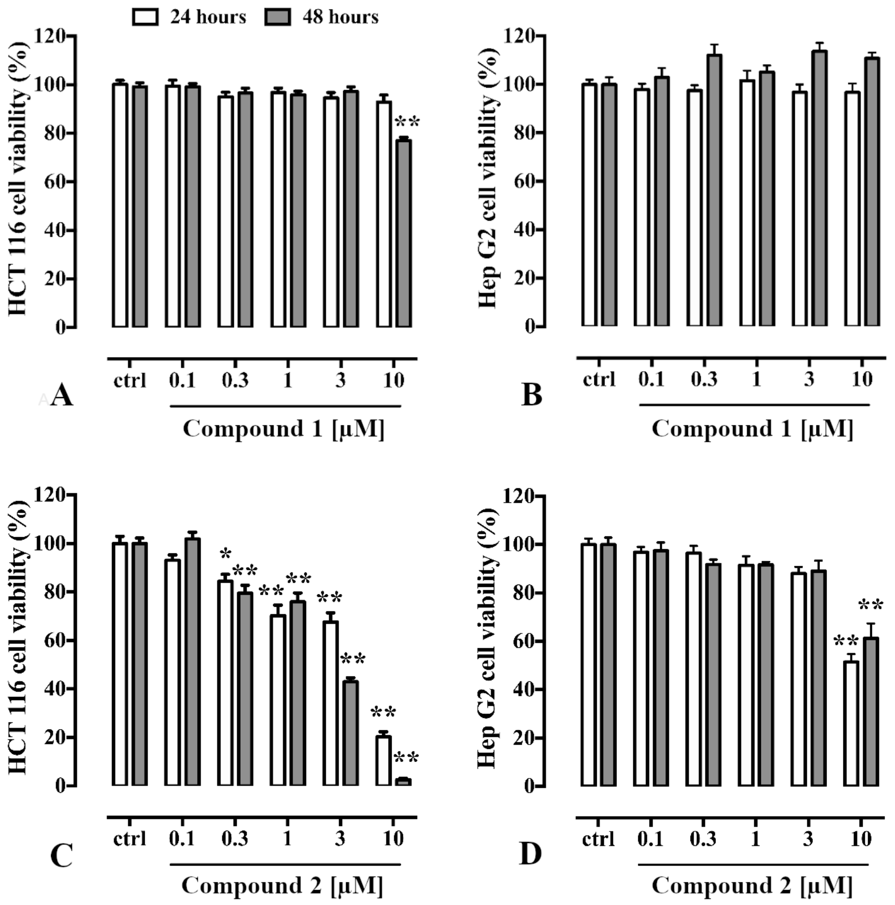

Figure S28. Cytotoxic effects of zosterabisphenone A (**1**) (panels A, B) and B (**2**) (panels C, D) on HCT116 and Hep G2 cells (0.1–10 μg/mL at 24-h and 48-h exposure). Cell viability rate (expressed as percentage) was investigated by using the MTT assay. Each bar represents the mean ± SEM of three independent experiments (including 5–6 replicates for each treatment). * $p < 0.01$ and ** $p < 0.0001$ vs. control (ctrl, i.e. untreated cells).

Table S1. ^{1}H and ^{13}C NMR Data of zosterabisphenone A (**1**) (700 MHz, 253 K, $CDCl_3$)

Position	δ_C		δ_H, mult (*J* in Hz)	HMBC	ROESY
1	138.0 (C)		-		
1-OH			6.74, s	1,2,6	-
2	122.4 (C)		-		
3	139.7 (C)		-		
4	137.3 (C)		-		
5	103.0 (CH)		6.80, s	1,3,6,7	7, 6-OMe
6	145.9 (C)		-		
6-OMe	56.2 (CH_3)		3.96, s	6	5
7	128.8 (CH)		6.25, s	3,4,9,3'	5,7'$_{proR}$
8	148.3 (C)		-		
9	51.3 (CH)		5.01, br. d (11.2)	3,7,8,10,11	12$_{proR}$,16,2',3'
10	127.4 (CH)		4.76, t (11.2)	9,12	11,3'
11	134.4 (CH)		5.55, ddd (11.8, 11.2, 4.8)		10,12$_{proS}$,13$_{proR}$,16'
12	27.6 (CH_2)	pro*R*	3.05, dddd (13.6, 12.6, 11.8, 7.0)		9,12$_{proS}$,13$_{proS}$,16,2'
		pro*S*	2.56, overlapped	10,11,17	11,12$_{proR}$
13	34.1 (CH_2)	pro*R*	2.71, ddd (14.4, 12.6, 6.0)	16,17,18	11,18
		pro*S*	3.39, dd (14.4, 7.0)	12,16,17,18	12$_{proR}$,16
14	152.0 (C)		-		
14-OH	-		7.57, s	14,15,19	-
15	122.7 (C)		-		
16	135.1 (CH)		7.68, br. s	2,13,14, 18	9,12$_{proR}$,13$_{proS}$
17	131.9 (C)		-		
18	129.7 (CH)		6.91, br. d (8.0)	13,14,16	13$_{proR}$
19	116.7 (CH)		6.86, d (8.0)	14,15,17	-
1'	103.9 (C)				
1'-OH			5.27	1', 2', 6'	2'
2'	50.2 (CH)		3.85, s	8,1',3',4',6',14',15',16'	9,12$_{proR}$,1'-OH,3',16'
3'	46.1 (CH)		3.56, s	7,8,1',2',4',5',7',15'	7,9,10,2',7'$_{proS}$,9',16'
4'	163.3 (C)		-		
5'	122.6 (CH)		6.18, s	8,1',3',7'	7'$_{proR}$,8'
6'	189.1 (C)		-		
7'	38.5 (CH_2)	pro*R*	2.97, dd (12.4, 7.4)	3',4',5',8',9'	7,5',7'$_{proS}$,8'
		pro*S*	2.33, overlapped		3',7'$_{proR}$,9'
8'	124.5 (CH)		5.08, ddd (15.3, 8.0, 8.0)	10'	5',7'$_{proR}$,10'
9'	128.1 (CH)		5.89, dd (15.3, 10.6)	7',11'	3',7'$_{proS}$,12'$_{proS}$,16'
10'	129.2 (CH)		5.65, t (10.6)	8',9',12'	8',11'
11'	129.4 (CH)		5.22, ddd (11.5, 10.6, 5.3)		10',12'$_{proR}$
12'	29.4 (CH_2)	pro*R*	2.31, overlapped		11',12'$_{proS}$
		pro*S*	2.53, dq (3.3,12.2)		9',12'$_{proR}$,13'$_{proR}$,16'
13'	33.6 (CH_2)	pro*R*	2.98, overlapped		12'$_{proS}$,13'$_{proS}$,16'
		pro*S*	2.34, overlapped		13'$_{proR}$,18'
14'	154.9 (C)		-		
15'	127.8 (C)		-		
16'	123.4 (CH)		6.85, br. s	2',13',14',18'	11,2',3',9', 12'$_{proS}$,13'$_{proR}$
17'	133.9 (C)		-		
18'	130.5 (CH)		6.70, br. d (8.0)	13',14',16'	13'$_{proS}$
19'	109.0 (CH)		6.56, d (8.0)	14',15',17'	-

Table S2. ^1H and ^{13}C NMR Data of zosterabisphenone B (**2**) (700 MHz, 238 K, $CDCl_3$).

Position	δ_C		δ_H, mult (*J* in Hz)	HMBC	ROESY
1	138.0 (C)		-		
1-OH			6.69, s	2	6-OMe
2	122.4 (C)		-		
3	139.6 (C)		-		
4	136.8 (C)		-		
5	102.6 (CH)		6.85, s	1,3,6,7	6-OMe,7
6	145.6 (C)		-		
6-OMe	56.1 (CH_3)		3.97, s	6	1-OH,5
7	134.7 (CH)		6.26, s	3,4,9,3'	5,3'
8	141.1 (C)		-		
9	50.6 (CH)		4.76, br. d (11)	3,8,10,11	12_{proR},16
10	126.3 (CH)		4.36, t (11)	12	11
11	132.2 (CH)		4.93, ddd (11, 11, 5)	-	10,12_{proS},
12	27.6 (CH_2)	pro*R*	2.72, m	-	9,12_{proS},13_{proS},16
		pro*S*	2.17, m	17	11,12_{proR},13_{proR}
13	33.9 (CH_2)	pro*R*	2.53, ddd (13, 13, 6)	16,17,18	12_{proS},13_{proS},18
		pro*S*	3.26, dd (13, 6)	11,17	12_{proR},13_{proR}
14	151.5 (C)		-		
14-OH	-		7.51, s	14,19	-
15	122.3 (C)		-		
16	134.9 (CH)		7.53, br. s	2,13,14,18	9,12_{proR}
17	132.7 (C)		-		
18	129.7 (CH)		6.81, br. d (8)	13,14,16	13_{proR}
19	116.6 (CH)		6.77, d (8)	14,15,17	
1'	113.6 (CH)		6.34, s	2',3',5'	16'
2'	175.4 (C)		-		
3'	42.3 (CH)		4.62, s	7,8,9,1',2',4',5',7'	7,9',16'
4'	121.6 (C)		-		
5'	145.3 (C)		-		
5'-OH			6.43, s	4',5',6'	-
6'	183.2 (C)		-		
7'	27.6(CH_2)	pro*R*	2.35, dd (19, 6)	4',5',8',9'	$7'_{proS}$,8'
		pro*S*	3.41, br. d (19)	3',4',5',8',9'	$7'_{proR}$
8'	129.4 (CH)		5.60, br. dd (15, 6)	4',10'	$7'_{proR}$,10'
9'	128.0 (CH)		5.86, m	-	3',$12'_{proS}$,16'
10'	128.4 (CH)		5.90, m	8',12'	8',11'
11'	129.5 (CH)		5.34, ddd (11, 11, 5)	-	10',$12'_{proR}$,$13'_{proS}$
12'	27.9 (CH_2)	pro*R*	2.28, m	-	11',$13'_{proR}$,$12'_{proS}$
		pro*S*	2.65, br. quartet (13)	-	9',$12'_{proR}$,$13'_{proR}$,16'
13'	24.5 (CH_2)	pro*R*	2.43, br. d (14)	11',17'	$12'_{proR}$,$12'_{proS}$,$13'_{proS}$,16'
		pro*S*	3.01, br. t (14)	11',16',17',18'	11',$12'_{proR}$,$13'_{proR}$
14'	144.7 (C)		-		
14'-OH			5.25, s	-	
15'	136.4 (C)		-		
16'	119.1 (CH)		6.72, s	13',14',15',18'	1',3',9',$12'_{proS}$,$13'_{proR}$
17'	121.7 (C)		-		
18'	154.9 (C)		-		
18'-OMe			3.77, s	18'	19'
19'	99.4 (CH)		6.50, s	14',15',17',18'	18'-OMe

Table S3. Cartesian coordinates of the lowest-energy conformer of zosterabisphenone A (**1**) optimized at the B3LYP/6-31+G(d,p) level.

conformer 1, *E* = -1957.9933839 Harthrees >99% population			
C	-0.8302212481	3.3360981514	-4.0016888195
C	-1.30728812	2.3620698545	-3.1098754281
C	-0.3618412221	1.8354251534	-2.1939006135
C	0.9610354032	2.3153920842	-2.1761052344
C	1.4121077453	3.2964924499	-3.0675897145
C	0.4995897203	3.7974194683	-3.9885354095
C	1.7134152178	1.6338681012	-1.1269619797
C	0.9333525279	0.7229967886	-0.5072900982
C	-0.4656212651	0.713373843	-1.1394505371
C	-1.5753078221	0.7976196798	-0.1089810056
C	-2.5567786924	-0.0919583558	0.1260603994
C	-2.8885096942	-1.3543031146	-0.6383490534
C	-4.1625601176	-1.2210392268	-1.5449773523
C	-3.8401633089	2.8223617612	-2.9937235948
C	-2.736669641	1.9363635752	-3.0673465709
C	-2.9872567506	0.583605587	-2.8116820278
C	-4.1797096687	0.1114639926	-2.2676540562
C	-5.2441042999	1.0154903715	-2.1688286537
C	-5.0890663156	2.3376310687	-2.5836657951
C	2.0823108716	5.2741341821	-5.0412678128
C	2.946134361	-1.9941237492	-0.4071699148
C	1.5051443859	-1.7045399236	0.0970207019
C	1.2952693647	-0.2423477928	0.6047965125
C	2.4830301555	0.2727400028	1.3979511879
C	3.7436745419	-0.0054586338	0.9992634367
C	3.9911190801	-0.8859778053	-0.1411397972
C	2.1854246081	1.1336156518	2.6150424402
C	2.1969723958	0.2628233564	3.8566655382
C	1.1383008362	-0.414390669	4.3386391115
C	1.2177943048	-1.3388888613	5.4659006745
C	0.3372329933	-2.3125228365	5.7757646621
C	-0.8936445204	-2.7095044925	5.0074279518
C	-0.6897527985	-4.0040769546	4.1613939109
C	2.4998347364	-3.4592012754	1.3259220207
C	1.3270451728	-2.7188328012	1.2011596401
C	0.2702117571	-2.9357029457	2.0748731967
C	0.3979322434	-3.8751209944	3.1139284869
C	1.5865112449	-4.6158526375	3.1998326232
C	2.6514638823	-4.431489465	2.3069806892
O	-1.6919054212	3.8746946588	-4.9365905276
O	0.7595419817	4.750893764	-4.9453879211
O	-3.7506490712	4.165541115	-3.2171110521
O	4.9901828143	-0.8443826084	-0.8519116253
O	2.9503572847	-2.3566526499	-1.7371651263
O	3.4609866563	-3.0999164414	0.4203216506
H	2.4381559585	3.6439308274	-3.0355852994
H	2.755062404	1.8368220509	-0.9058994971
H	-0.5514133344	-0.2419445131	-1.6733012747
H	-1.5399681635	1.6896308119	0.5163755884
H	-3.2565307977	0.1585337791	0.924466513
H	-2.042028508	-1.6589700344	-1.2615978843
H	-3.0554682815	-2.1792928764	0.0663867405
H	-4.1783167953	-2.076940719	-2.2325256861
H	-5.0635590565	-1.3013994265	-0.9259906435
H	-2.1576148591	-0.1035855516	-2.9164146366
H	-6.1919518943	0.6972120027	-1.7407096868
H	-5.9118876879	3.0432751988	-2.5281387976
H	2.0615193184	5.9915192353	-5.8619892799
H	2.3697442783	5.784544589	-4.1147050787
H	2.8024537129	4.4787818343	-5.265385414
H	0.8236967727	-1.8930627774	-0.7384771892
H	0.4302761673	-0.2836530893	1.276701934
H	4.6107583148	0.4542516191	1.4676009932
H	1.2108703414	1.6181047781	2.4934142515
H	2.9386709973	1.9231786449	2.7084614586
H	3.1721186672	0.1135058287	4.3200267237
H	0.1739149639	-0.2965593386	3.8473241025
H	2.1082505346	-1.2451086534	6.0870002137
H	0.5707409838	-2.9375167349	6.6377431528
H	-1.7199375441	-2.8920880032	5.7071869209
H	-1.2202158907	-1.9015790444	4.3447519334
H	-1.6502482764	-4.2490322736	3.6886467182
H	-0.4521586737	-4.837128577	4.8331993118
H	-0.643234342	-2.3520478342	1.9809984836
H	1.696035624	-5.345768879	3.9979206706
H	3.5692810868	-5.003273964	2.3916858959
H	-1.1903493486	4.5149287443	-5.4680558512
H	-3.0523380667	4.3341753813	-3.8766830573
H	3.8607410849	-2.2192694623	-2.0590284434

Table S4. Cartesian coordinates of the lowest-energy conformers of the C-9 epimer of zosterabisphenone A (*epi*-**1**) optimized at the B3LYP/6-31+G(d,p) level.

Conformer 1, E = -1957.9916158 Hartrees				Conformer 2, -1957.9906194 Hartrees			
ΔE = 0.00 kcal/mol, population 77.6%				ΔE = 0.63 kcal/mol, population 22.4%			
C	4.9624260509	0.7058031051	-0.4374890323	C	-1.0095253365	3.7796838506	-3.5480806728
C	3.8963971201	-0.1947465935	-0.2829552745	C	-1.3934263001	2.815341615	-2.60191899
C	2.593449951	0.3624903501	-0.3325663153	C	-0.3463694195	2.0502604632	-2.025276921
C	2.4143216762	1.7490545181	-0.4985771317	C	0.9829917579	2.2323734233	-2.4509851966
C	3.4934424062	2.6301440795	-0.641855566	C	1.3405118692	3.1870869888	-3.4107991062
C	4.7735121886	2.0899528009	-0.6122171115	C	0.3261901685	3.9696772882	-3.9480587334
C	0.9888394319	2.0609576603	-0.5010117424	C	1.854783574	1.2968557893	-1.750215629
C	0.262275536	0.9340082806	-0.3442100087	C	1.1437430853	0.5615779061	-0.8701547533
C	1.1943764366	-0.2843106243	-0.2810820754	C	-0.3343514984	0.9834487657	-0.9050538771
C	0.8797996476	-1.231224747	0.8611096513	C	-1.2714248933	-0.2026613055	-1.0404664438
C	0.5133587421	-2.5233828913	0.7884443674	C	-2.2639847535	-0.5845885434	-0.2160068959
C	0.4292361209	-3.4144423104	-0.4308398234	C	-2.8015108512	0.1150833687	1.013206563
C	1.6040481278	-4.4512478797	-0.527937033	C	-4.1978119198	0.7912013342	0.7900047499
C	4.9453054435	-2.1864517517	0.9752982777	C	-3.8469620083	2.3235952992	-3.2189986194
C	4.0990243601	-1.6505870741	-0.0270049084	C	-2.8224773197	2.5495187657	-2.2655326164
C	3.1972043646	-2.5300408272	-0.6365051801	C	-3.1137994276	2.2391234511	-0.9331027158
C	2.9269593368	-3.8100257335	-0.1579004567	C	-4.2396059573	1.5196489811	-0.5383724202
C	3.7518916495	-4.303126344	0.8601345127	C	-5.2210585045	1.2768841991	-1.5064745375
C	4.7838728581	-3.5196783015	1.3750425834	C	-5.0483202965	1.7267425346	-2.8145475414
C	5.880359173	4.2065600737	-0.9099709287	C	1.7976386718	5.2444332122	-5.3631019697
C	-2.1744149319	2.3541624378	1.5830834071	C	4.2840665151	-0.5668957281	-0.197617462
C	-1.8437805229	0.8981714754	1.1503998457	C	2.9242472543	-1.2553055847	-0.5134817848
C	-1.2481602779	0.7783579743	-0.289454259	C	1.6769199235	-0.5192778238	0.0565903171
C	-1.9051141727	1.7332116623	-1.2712674103	C	1.943210195	0.0504762739	1.452420721
C	-2.2319505624	2.9903166759	-0.8973632364	C	3.1268913511	0.6331635075	1.7442741323
C	-2.0570536682	3.4333584736	0.4835387445	C	4.2264882259	0.6266303294	0.7847616924
C	-2.1367349924	1.220960384	-2.6836817546	C	0.8310275157	-0.0575198373	2.4826724551
C	-3.5517673764	0.6854815001	-2.7916375904	C	1.0524980644	-1.296712844	3.328751489
C	-3.9327737697	-0.5693212502	-2.4877102568	C	0.5775754359	-2.522472912	3.0391677546
C	-5.3239301031	-1.0123945016	-2.4850358774	C	0.8939403877	-3.7173433208	3.816426595
C	-5.8254472073	-2.0981128402	-1.8610867195	C	0.7817440303	-4.9977476467	3.4072540234
C	-5.0878192884	-3.0701276375	-0.9813233465	C	0.371865262	-5.4867723044	2.0449825563
C	-5.3507116313	-2.8351852368	0.538481465	C	1.5846244009	-5.9443619709	1.1762349376
C	-4.1395042203	1.1364054609	1.6597901138	C	4.3588338778	-2.6951562725	0.6953516187
C	-3.1828250537	0.2074134934	1.258830867	C	3.1017513898	-2.6245082292	0.1013717611
C	-3.5602318027	-1.0946444613	0.9602896861	C	2.2275458183	-3.6986975627	0.1961778179
C	-4.9156955694	-1.4655999593	1.0193935391	C	2.594231424	-4.8418785083	0.9293451014
C	-5.8506252233	-0.5095096163	1.4439681683	C	3.8754050899	-4.8856686118	1.4993727583
C	-5.4822671349	0.8002643584	1.7816798695	C	4.7829446851	-3.8234057447	1.3858225096
H	3.3264369012	3.6938024732	-0.7653353705	H	2.3740561775	3.3042758827	-3.7147740885
H	0.5955784963	3.0627473909	-0.6316739566	H	2.9212241563	1.2203381574	-1.9326987543
H	1.0316810724	-0.8157646775	-1.2293510075	H	-0.5404031842	1.4940444555	0.0453881561
H	0.9548075912	-0.7826696757	1.8508586566	H	-1.0816137495	-0.8199603019	-1.918479028
H	0.3247141429	-3.0266439467	1.7373277011	H	-2.81047061	-1.4838256385	-0.5032471874
H	0.4129369926	-2.8133590489	-1.3458248442	H	-2.0978183915	0.8780867872	1.3598110224
H	-0.5166770886	-3.971880426	-0.4171774266	H	-2.8973485949	-0.6061185913	1.8355087362
H	1.6039403097	-4.8689729742	-1.5435006711	H	-4.3936777549	1.4523756475	1.6446725657
H	1.4113859702	-5.2878151227	0.1534338013	H	-4.9829037515	0.0263902903	0.8015164141
H	2.583290063	-2.1355172451	-1.436093346	H	-2.3503327547	2.4543109759	-0.1967162677
H	3.5733559647	-5.2922105577	1.2758252513	H	-6.1125481037	0.709402189	-1.2491479141
H	5.4402368976	-3.8971120222	2.1526749162	H	-5.8084707371	1.5588115438	-3.570842054
H	6.9125923412	4.5491051949	-0.9859687659	H	1.68757068	6.0573188815	-6.081265937
H	5.3382416926	4.4664202923	-1.8265708704	H	2.452886256	5.5628227671	-4.5441571143
H	5.3981274346	4.6797439365	-0.0469209614	H	2.2270331852	4.3682616013	-5.8624734522
H	-1.1208029971	0.4983266991	1.8659564713	H	2.8268709735	-1.3133299708	-1.6022088578
H	-1.4680098095	-0.2444684162	-0.6137180869	H	0.8869553806	-1.2717818058	0.1566778868
H	-2.5754879382	3.7315131799	-1.6150476498	H	3.2915127269	1.158838395	2.6816571503
H	-1.4090215385	0.435626943	-2.9155088993	H	-0.1380502532	-0.1188204128	1.9814819072
H	-1.9897457799	2.0310586758	-3.4056936352	H	0.8253195756	0.832508127	3.1214558649
H	-4.3165017083	1.4204656603	-3.0420980058	H	1.7235228804	-1.1768589473	4.1791002851
H	-3.1721104203	-1.2912264323	-2.1950177228	H	-0.0553874678	-2.642587091	2.1615945714
H	-6.0228240347	-0.3639322598	-3.0127300824	H	1.2967061946	-3.5367012343	4.8127141912
H	-6.9007224625	-2.2605622478	-1.9355655973	H	1.0929294524	-5.7727172085	4.10775918
H	-5.402242276	-4.0942387155	-1.2226667859	H	-0.3088166482	-6.3419161727	2.1499277707
H	-4.0094009699	-3.0244636425	-1.164628881	H	-0.180339232	-4.713911685	1.5006301108
H	-4.8241804663	-3.6225632204	1.0941574206	H	1.1901285146	-6.3248879139	0.224820033
H	-6.4192291618	-2.9716007817	0.7420123039	H	2.0796662038	-6.7890989375	1.6692073722
H	-2.811974296	-1.819441875	0.6468427756	H	1.2426599704	-3.644577948	-0.2634070636
H	-6.900862113	-0.7854532866	1.4953002429	H	4.1702297118	-5.7649705285	2.0664035085
H	-6.2164394239	1.5332134054	2.0983827598	H	5.7670286267	-3.8653532484	1.8400066913
H	6.8546104466	0.9840938731	-0.5440728502	H	-1.5276584683	5.1871090414	-4.7394400464
H	6.2133775435	-0.7488178973	1.0747992003	H	-3.1150769121	3.33533524	-4.6761953503
H	-1.4764888114	3.6944728704	2.7370241989	H	5.621594446	0.4495017396	-1.0883303618
O	6.2576055177	0.2277553589	-0.4194167542	O	-1.9751559184	4.582151378	-4.124780172
O	5.9489444331	2.7921208775	-0.7426761868	O	0.4838712052	4.964889689	-4.8843047888
O	5.8732731553	-1.4542520469	1.6558571857	O	-3.7114694567	2.5733003773	-4.5532769625
O	-1.8856380328	4.5995361391	0.8259051712	O	5.1327658232	1.4526548337	0.7469046267
O	-1.4584122855	2.7200610654	2.7026089687	O	4.9454338694	-0.2010089278	-1.3556298475
O	-3.6193434649	2.3832043001	1.8764442731	O	5.0872464913	-1.5450160329	0.5481465362

Table S5. Cartesian coordinates of the lowest-energy conformers of zosterabisphenone B (**2**) optimized at the B3LYP/6-31+G(d,p) level.

Conformer 1, *E* = -2147.7357258 Harthrees ΔE = 0.00 kcal/mol, population 85.4 %				Conformer 2, *E* = -2147.7342802 Harthrees ΔE = 0.91 kcal/mol, population 14.6%			
C	-3.9540693072	2.6117500189	0.2511530279	C	-5.033172493	0.2404203709	0.1983802419
C	-3.6353556242	1.266071911	0.0043509724	C	-3.8404870028	-0.355976338	-0.2420827363
C	-2.2767255582	0.8977939612	0.1870112582	C	-2.6591969717	0.4142309946	-0.0862159231
C	-1.3409017107	1.8495062528	0.6360815255	C	-2.7118674573	1.6827239284	0.5231104436
C	-1.6834389561	3.1831886585	0.889383472	C	-3.9114502339	2.2518543687	0.9689011338
C	-3.0054838653	3.5567138814	0.6841050472	C	-5.0767822216	1.5163488956	0.7910963514
C	-0.0326698056	1.218827088	0.7589083499	C	-1.3687746722	2.2460906078	0.5901169628
C	-0.0961813855	-0.0786462313	0.3961713963	C	-0.479576379	1.4066562571	0.0219714577
C	-1.5199101726	-0.432613426	-0.0592798011	C	-1.1967036079	0.1598769134	-0.515260854
C	-2.0854758944	-1.6942931868	0.5693751753	C	-0.5368181998	-1.1372087024	-0.0883162738
C	-2.5238103348	-2.8036354712	-0.0497521513	C	-0.0231783457	-2.1002091645	-0.8736284191
C	-2.643932661	-3.0827006113	-1.5310454336	C	-0.0556130288	-2.2164822866	-2.3802793979
C	-4.1240919156	-3.0880295762	-2.048285	C	-1.0647786776	-3.2968007895	-2.90388704
C	-5.8869510287	0.05021895	0.3121134042	C	-4.3578135285	-2.8799413517	-0.1594608411
C	-4.6676354053	0.2599858415	-0.3788386347	C	-3.7898403346	-1.743620291	-0.7877502266
C	-4.2907564523	-0.7080121008	-1.3153155575	C	-2.9002602916	-1.9769750632	-1.8419794063
C	-4.9121462479	-1.9493333294	-1.4318907553	C	-2.3742591663	-3.2312225062	-2.1429389943
C	-6.0972303375	-2.1450259145	-0.7131819451	C	-2.9259646922	-4.3350060505	-1.4819725143
C	-6.60493922	-1.1338372289	0.1017341645	C	-3.9451966595	-4.1615797085	-0.5465751101
C	-2.6500376864	5.8597450591	1.28489233	C	-6.5096835196	3.1930530546	1.7549331863
C	1.1437435716	-2.8060339479	-1.3996249638	C	1.8762420702	3.5196631452	1.298659068
C	1.3308830707	-1.5208108534	-1.0462102185	C	1.5852029556	2.2156815704	1.1254313085
C	1.1211173931	-1.002562904	0.3557989751	C	1.0257452733	1.6523020575	-0.1610993934
C	1.0940561649	-2.0894749489	1.4163383037	C	1.2941232882	2.541935601	-1.3700127282
C	0.9369654533	-3.3784140171	1.0516602903	C	1.6004865917	3.8437040344	-1.18480813
C	0.874215763	-3.805253237	-0.3702694564	C	1.8408494796	4.4286206768	0.1591574363
C	1.2311869847	-1.6544321181	2.8555917736	C	1.168522317	1.9393455368	-2.7483929198
C	2.5351777716	-0.9478181256	3.1774561422	C	2.1458768398	0.8165205904	-3.0365879085
C	3.6712824664	-1.0312551907	2.4594434845	C	3.2890418863	0.5772192343	-2.3678627711
C	4.8771201313	-0.2593507791	2.7276170381	C	4.1530139692	-0.5709412403	-2.6048672072
C	5.9925825935	-0.2210499891	1.9683199719	C	5.2548096344	-0.9090659842	-1.9029775816
C	6.2843612801	-1.0031384164	0.7151722813	C	5.8994610901	-0.1568384289	-0.7690054204
C	6.4012169839	-0.1371944494	-0.5691225939	C	5.8440488715	-0.8885868688	0.5979122569
C	2.6512868051	1.5842821612	-1.9606881625	C	1.9899371225	-0.9559056497	2.6222660367
C	2.7767932193	0.2114418728	-1.7487294973	C	2.4728995431	0.2160622018	2.0426604229
C	3.9903592125	-0.3267629804	-1.3260412178	C	3.7108086966	0.2299911274	1.4052553658
C	5.0988145068	0.4762493276	-1.0504936055	C	4.4833062082	-0.9259613814	1.2725676923
C	4.9581256525	1.8668120614	-1.2635693532	C	3.9755668871	-2.1122608188	1.8511353532
C	3.7528643177	2.4114178611	-1.7238373007	C	2.7483422153	-2.1245090601	2.5260857892
C	5.98651697	4.0393992805	-1.1908093355	C	4.3040151474	-4.4565953184	2.2770303992
H	-0.9361176537	3.8911850353	1.2280801373	H	-3.9209486145	3.2318560598	1.4315810479
H	0.8668274053	1.7394046368	1.072521979	H	-1.1408828103	3.2128061016	1.027357854
H	-1.4580931874	-0.5861724082	-1.1451834926	H	-1.1524556188	0.2310462576	-1.6098339595
H	-2.1170922841	-1.6772969227	1.6577957025	H	-0.4641630826	-1.2742440273	0.9889282776
H	-2.8875015972	-3.6031408319	0.5963749059	H	0.4244638842	-2.9511111632	-0.3595491237
H	-2.0697178006	-2.3500014763	-2.1069653599	H	-0.3036588953	-1.2521502744	-2.8342557269
H	-2.1950023144	-4.0587830685	-1.7536676134	H	0.9460918989	-2.4734170703	-2.7471104927
H	-4.6036666997	-4.0371736126	-1.7829516323	H	-0.631290814	-4.2958316288	-2.7788712678
H	-4.1024538666	-3.0450888179	-3.1453147563	H	-1.1938987459	-3.1458806393	-3.9840317146
H	-3.3816103492	-0.529863161	-1.8752068741	H	-2.5012605456	-1.1113812802	-2.3547005437
H	-6.6123242657	-3.101691127	-0.7619375168	H	-2.5407754179	-5.3339157537	-1.6739611123
H	-7.5302600548	-1.2686470764	0.6528010107	H	-4.391695254	-5.0108205214	-0.0391590452
H	1.2590817712	-3.1404957281	-2.4250366326	H	2.2118624365	3.9045353405	2.2555382402
H	1.9699133261	-0.3512902509	0.5893000785	H	1.487163085	0.6751967765	-0.3421561597
H	1.1040831051	-2.5257042221	3.5092020973	H	1.2813673132	2.733926512	-3.4956159233
H	0.4084815726	-0.9685546077	3.1056639023	H	0.1494750872	1.5501889386	-2.8908695048
H	2.5151158797	-0.3049154557	4.0581545091	H	1.8638111315	0.1478039709	-3.8500863751
H	3.683313631	-1.6852600292	1.5904309086	H	3.5700488177	1.2556676728	-1.5659981948
H	4.8452805421	0.3749914739	3.6136049648	H	3.8429160767	-1.2359282624	-3.4110272315
H	6.7894863495	0.4512926736	2.2863757973	H	5.757479955	-1.835376135	-2.1816849302
H	5.5440946895	-1.7945472874	0.5633449636	H	5.4850226736	0.8515728731	-0.6773790731
H	7.2482403475	-1.5147669656	0.8461735148	H	6.9618331617	-0.0195449853	-1.0145450745
H	7.1327245202	0.6569113921	-0.4035428311	H	6.2072134952	-1.9113604486	0.4723827375
H	6.8099936574	-0.7742460002	-1.3653189136	H	6.5567510805	-0.3938315331	1.2728252967
H	4.0611496846	-1.4032305047	-1.1985772571	H	4.0748588933	1.1709697689	1.004730419
H	3.6305692673	3.4730661736	-1.8958774716	H	2.351289716	-3.022559524	2.9815960916
H	-3.2640998943	6.7581360207	1.3524835547	H	-7.5779137655	3.2978669835	1.9461258898
H	-1.8481027165	6.0160429298	0.5540097148	H	-6.1764801414	3.9907426903	1.0809645142
H	-2.2180363904	5.6355920178	2.2672067311	H	-5.9595174718	3.25297168	2.7011666177
H	6.9693492744	4.4279943266	-0.920993719	H	5.0790793308	-5.18933281	2.0486226641
H	5.2259392773	4.4904160838	-0.5411540631	H	3.3571157131	-4.7746768108	1.8232690614
H	5.770617056	4.291306073	-2.2365872834	H	4.1819027208	-4.3837669681	3.3648746507
H	0.8129975693	1.4515113904	-2.4464740127	H	0.4097867003	-0.1004470213	3.2633376714
H	0.7291335951	-5.2064020383	1.3957362611	H	1.9918258733	5.575269254	-1.7806857775
H	-5.2822813566	3.9917375672	0.2657786348	H	-6.9343401819	0.1202823323	0.3996822692
H	-6.1271325537	1.8156582614	1.0249019344	H	-5.7769679352	-2.0044123583	0.790998388
O	1.4854882273	2.1473885623	-2.3879379782	O	0.7918896748	-0.9919146782	3.2753733618
O	1.6549105936	-0.592773672	-2.014946948	O	1.6877380662	1.3680115649	2.2045337314
O	0.6856765281	-5.0042684064	-0.6157675292	O	2.092990391	5.6386649668	0.2378160757
O	0.8450345329	-4.4012734269	1.9395109771	O	1.7665543277	4.7239583268	-2.2071039205
O	6.0624556933	2.6326059191	-1.0011322985	O	4.7588562016	-3.228606718	1.7230089937
O	-5.2523467302	3.0421409561	0.0629707604	O	-6.2238127275	-0.4436696564	0.0518651778
O	-3.5262012041	4.817433062	0.8639553394	O	-6.3442060512	1.9129468728	1.1497320879
O	-6.3807579198	0.9035433886	1.2569624039	O	-5.2378272357	-2.8118508403	0.8812029228

Table S6. Cartesian coordinates of the lowest-energy conformers of the C-9 epimer of zosterabisphenone B (*epi*-**2**) optimized at the B3LYP/6-31+G(d,p) level.

Conformer 1, *E* = -2147.7361081 Harthrees				Conformer 2, *E* = -2147.7347249 Harthrees			
Δ*E* = 0.00 kcal/mol, population 84.5 %				Δ*E* = 0.87 kcal/mol, population 15.5%			
C	3.9724629296	2.5575843289	-0.7100062783	C	4.982891869	0.2122387687	-0.550297574
C	3.6730965553	1.2178066469	-0.4118908415	C	3.7955927151	-0.4256339482	-0.1550636816
C	2.2968198955	0.87254825	-0.3843826474	C	2.632785082	0.3864937199	-0.1135190943
C	1.3155062298	1.8579365735	-0.6056127588	C	2.714617661	1.7631210419	-0.4006376931
C	1.6360712011	3.1918150195	-0.8853034029	C	3.9147982779	2.3824592292	-0.7716964212
C	2.9822580289	3.5303189551	-0.9414456416	C	5.0499623553	1.5855068405	-0.8526660304
C	-0.0069219136	1.2525225872	-0.505637238	C	1.3955362569	2.3673974477	-0.2598485982
C	0.0954195658	-0.0664802075	-0.2417929965	C	0.4856209964	1.4316233931	0.0771243184
C	1.5718411185	-0.4813164691	-0.1804587988	C	1.1532428113	0.0542640491	0.1795732468
C	1.9408899276	-1.3009376563	1.0433984246	C	0.8438608792	-0.6664127568	1.4785675677
C	2.4627964895	-2.5386218225	1.0840520435	C	0.2432229379	-1.8575222723	1.6439645917
C	2.8952534436	-3.4250821492	-0.0619314205	C	-0.1982202161	-2.8467760654	0.5905186939
C	4.452179333	-3.5529296373	-0.2012387556	C	0.7244227366	-4.112157516	0.4971435055
C	5.756751965	0.4065245797	0.8698528564	C	4.6263868699	-2.4416216371	1.2163587886
C	4.7295321835	0.2159392902	-0.0876306958	C	3.7527728933	-1.8567334506	0.2653657227
C	4.535819215	-1.0846345385	-0.5647693904	C	2.6166250337	-2.5912226559	-0.0928602061
C	5.1231855036	-2.2101514338	0.0087737116	C	2.1871405235	-3.7256063293	0.5929976095
C	6.111605951	-1.9969716542	0.9767467266	C	3.0522608257	-4.2643951888	1.5525141833
C	6.4614680027	-0.7017464688	1.356578511	C	4.2810513476	-3.660245172	1.8146285774
C	2.5762901643	5.8460059898	-1.4556928964	C	6.4963805817	3.3837383645	-1.5366781067
C	-1.2264699118	-2.6956699544	1.7131348673	C	-1.8915076841	3.4133195993	-1.348525453
C	-1.3861846089	-1.4376077365	1.2641839879	C	-1.5672966554	2.1314999607	-1.0938523647
C	-1.1042004505	-1.0099607698	-0.1584569301	C	-1.0275836559	1.6543157875	0.2364950707
C	-0.9905925923	-2.1650964208	-1.1355309544	C	-1.3447049908	2.6022710466	1.3830286256
C	-0.823915766	-3.4213244594	-0.6717393962	C	-1.6814863341	3.8826844903	1.1191043389
C	-0.8660252636	-3.7538696859	0.7781903687	C	-1.910157132	4.3846281726	-0.2603968668
C	-1.0593477211	-1.8347765434	-2.6084546632	C	-1.2390729295	2.0675754997	2.7898344018
C	-2.3556941806	-1.1764232261	-3.0441327495	C	-2.2140652755	0.9485526494	3.1018610233
C	-3.5285060551	-1.2372985604	-2.3854995166	C	-3.352377119	0.6904420806	2.4318191737
C	-4.7281426059	-0.5056084218	-2.7698776254	C	-4.2097494762	-0.4595756116	2.6842756225
C	-5.8844053119	-0.4346988947	-2.0770114931	C	-5.295085688	-0.8294067972	1.9724766447
C	-6.2370413037	-1.1342566281	-0.7912805356	C	-5.9232808685	-0.1182512777	0.8030653253
C	-6.4434527516	-0.183242964	0.4192810284	C	-5.8525208345	-0.9019492915	-0.5340150045
C	-2.8122207644	1.6922626411	1.9226941273	C	-1.9426547104	-1.1192224619	-2.4342618091
C	-2.897407903	0.3078210958	1.7749063537	C	-2.4254471678	0.0869508861	-1.9299883405
C	-4.0740420407	-0.2786711871	1.3139025998	C	-3.6826101871	0.1480566553	-1.3337136939
C	-5.181377531	0.4835426274	0.9363708271	C	-4.4775136532	-0.9892245617	-1.1741835973
C	-5.0813753214	1.8858862225	1.0849725504	C	-3.9745977275	-2.2074281931	-1.6866433642
C	-3.9150126836	2.4808677712	1.5821187525	C	-2.7251294801	-2.2696218168	-2.3170362857
C	-6.148792374	4.0275425567	0.8453806516	C	-4.3395267621	-4.5592802352	-2.0368847599
H	0.8553583605	3.9258163844	-1.047068571	H	3.9465581758	3.4440651312	-0.987322163
H	-0.9359244716	1.7951920669	-0.6485475463	H	1.191300497	3.4191710896	-0.4331452891
H	1.7456148883	-1.0958331362	-1.0733690196	H	0.7579682238	-0.5403132716	-0.6545433291
H	1.7356526402	-0.8075358806	1.9927086877	H	1.1539863984	-0.1277895963	2.3728231688
H	2.6475418257	-2.9516884221	2.0761341141	H	0.1146722625	-2.1942212518	2.6732784091
H	2.4818453438	-3.0601848637	-1.0071117253	H	-0.2407956015	-2.369355113	-0.3915690076
H	2.4783584221	-4.4299960407	0.0797042169	H	-1.2211116522	-3.1792094508	0.8081065979
H	4.6711175899	-3.9881821304	-1.1852366708	H	0.4859687554	-4.635795	-0.4382760013
H	4.8320345896	-4.2606820833	0.5444583639	H	0.4867015341	-4.8048321318	1.3129324186
H	3.7808416005	-1.2268231895	-1.3273931531	H	1.9706217363	-2.1716820909	-0.8532363283
H	6.5905530588	-2.8443162027	1.4620748292	H	2.7560793597	-5.1426873756	2.1217097703
H	7.2361148061	-0.5234890226	2.0956473522	H	4.9672932317	-4.0769125974	2.5450492747
H	-1.4053580572	-2.9606994539	2.7494549234	H	-2.2063499824	3.7360889233	-2.3348644021
H	-1.9575716806	-0.4011906084	-0.4758136352	H	-1.4733134698	0.6809440338	0.467628377
H	-0.8860048593	-2.747501998	-3.1910836776	H	-1.371989258	2.8923681621	3.4999231696
H	-0.2376436968	-1.151068559	-2.8688302609	H	-0.2217959822	1.6880051364	2.9592454058
H	-2.2970686761	-0.58848763	-3.9606178016	H	-1.9294839098	0.2952507332	3.9267914922
H	-3.5801295974	-1.8355162476	-1.4785424105	H	-3.6312812532	1.3516577297	1.6145906581
H	-4.6550330708	0.0692742785	-3.6931716652	H	-3.9069265147	-1.099511559	3.5131659577
H	-6.6703201089	0.2020636438	-2.4834418071	H	-5.7948015596	-1.7518020321	2.2690841723
H	-5.4951758194	-1.8989102709	-0.5419474441	H	-5.5034528516	0.8843294026	0.6781262835
H	-7.1835177076	-1.6723191442	-0.9417945235	H	-6.9885740142	0.0322173999	1.0276635738
H	-7.1725334755	0.5855474941	0.153563018	H	-6.2366978953	-1.9127440108	-0.3780410974
H	-6.8942727725	-0.7694928842	1.2317270157	H	-6.5407475241	-0.4210526986	-1.2434125925
H	-4.1155163888	-1.3614985942	1.2389954871	H	-4.0432704148	1.1104943551	-0.9850715024
H	-3.8246557827	3.5523752417	1.7062803353	H	-2.3298622153	-3.1931964609	-2.7200749242
H	3.1884855698	6.7283954304	-1.6437190976	H	7.5514166414	3.4901303026	-1.7900979916
H	1.9376076174	6.0209003447	-0.5821111542	H	6.2580785165	4.0230521492	-0.6787649131
H	1.9542352134	5.6362652966	-2.3337180244	H	5.8806661461	3.6721251047	-2.3965263891
H	-7.1228584474	4.3792119947	0.5028770065	H	-5.1376321535	-5.266488552	-1.8075743161
H	-5.3626581937	4.4637179038	0.2162834358	H	-3.4143940289	-4.8828040413	-1.5438056338
H	-5.9968277567	4.3358434349	1.8872951501	H	-4.1836023724	-4.5264964407	-3.1223537525
H	-0.9999555254	1.6248579077	2.5118138762	H	-0.3459900435	-0.3105154777	-3.0985050386
H	-0.5774132661	-5.2647159163	-0.8719810386	H	-2.1387505771	5.6313466451	1.611200759
H	5.3078354783	3.8989910418	-1.0038867872	H	6.8531666616	0.0728865273	-0.9397852991
H	5.8920524679	2.3185527642	0.7760491903	H	6.1492722964	-1.3031718417	0.951840271
O	-1.682966549	2.3015936823	2.3830437677	O	-0.7134676975	-1.205418838	-3.0278497346
O	-1.7763950022	-0.4521599593	2.1467855461	O	-1.6146091326	1.2155128667	-2.1228226152
O	-0.6846360669	-4.9328709278	1.1106580117	O	-2.1965870154	5.5798193073	-0.4126555616
O	-0.6554038501	-4.5004542106	-1.4788090916	O	-1.8968337924	4.8119826363	2.0876890936
O	-6.1849094274	2.6117518561	0.7233230325	O	-4.7824029998	-3.3032697635	-1.5387129843
O	5.293339071	2.9533085443	-0.7819844765	O	6.1457373272	-0.526228984	-0.6490098468
O	3.4904157508	4.7798428981	-1.2128966222	O	6.3081947621	2.0078421214	-1.21421995
O	6.0580573073	1.6148815213	1.4299320815	O	5.7682462799	-1.8451206214	1.6673427238

Table S7. Cartesian coordinates of the lowest-energy conformer of the aromatic tautomer of zosterabisphenone A (**1a**) optimized at the B3LYP/6-31+G(d,p) level, along with calculated isotropic shielding constants and predicted chemical shifts calculated at the PBE0/6-311+G(2d,p)/PCM($CHCl_3$)//B3LYP/6-31+G(d,p) level.

conformer 1, *E* = -1957.9807093 Harthrees >99% population			
C	-2.0142010139	3.6080249593	-1.9358689038
C	-0.612673093	3.5605796004	-1.8570357437
C	-0.0654679587	2.5066778337	-1.0795596923
C	-0.905409037	1.5447366036	-0.4897970639
C	-2.3008227248	1.5975790582	-0.6038205196
C	-2.846593394	2.650415709	-1.3273863811
C	-0.1012171351	0.5592668163	0.2216686948
C	1.2129938351	0.8739630653	0.1600628463
C	1.3826065846	2.1646060579	-0.6791331967
C	2.3898040927	1.9894490711	-1.8016258685
C	3.5260754522	2.6771755137	-2.0050749496
C	4.0648758602	3.8578806553	-1.2284650023
C	3.929798698	5.2297165808	-1.9776368622
C	0.1457650662	4.8000324126	-3.9858327803
C	0.2688502226	4.5054951149	-2.6042662332
C	1.4521175629	4.9054488715	-1.9731610534
C	2.581089529	5.347558007	-2.6597003571
C	2.4434927164	5.5969058089	-4.0300393431
C	1.2226113418	5.3813714476	-4.6676474398
C	-5.1251719046	1.9584841683	-0.9790820353
C	4.1046079283	-1.5327431228	0.2860883813
C	2.7634069843	-1.1357782251	0.2725763148
C	2.4453463761	0.2000059715	0.6478236555
C	3.3920147991	0.9278168008	1.4085577528
C	4.7145022057	0.4737618226	1.4779064453
C	5.088558133	-0.7025380963	0.8320686331
C	2.8886998613	1.9499799992	2.4321305208
C	2.450286971	1.13775316	3.6456506762
C	1.2552538049	0.5215578391	3.7527364966
C	0.9697223108	-0.5302542061	4.7249549999
C	0.0125117321	-1.4819283719	4.6462854274
C	-1.0816684325	-1.6662252536	3.6200620067
C	-1.0454868664	-3.0130396575	2.8310573573
C	1.3822398652	-3.0643911255	-0.8013811742
C	1.6662107478	-2.1456331246	0.2207812888
C	0.8879023886	-2.1970622511	1.3934703474
C	-0.2133373423	-3.0332043708	1.5545116145
C	-0.5205910757	-3.8846554149	0.4789389595
C	0.271844848	-3.9111493103	-0.6696416232
O	-2.6189579828	4.6296416886	-2.6441760515
O	-4.1888546231	2.8779525904	-1.5338382712
O	-0.94292587	4.4715331783	-4.7397959393
O	6.3744106916	-1.2017426474	0.7885378117
O	4.431733161	-2.7783917652	-0.1989883422
O	2.1204481034	-3.1519609089	-1.9499091569
H	-2.923122394	0.8395750452	-0.1425061293
H	-0.5292286782	-0.2921835885	0.7313764335
H	1.7503016615	2.9426552123	-0.0016219511
H	2.1470283518	1.1888937932	-2.4989790017
H	4.1180440707	2.3894136266	-2.8745985169
H	3.5642453902	3.9346344013	-0.2582541449
H	5.1272618599	3.6929559563	-1.0051306572
H	4.1089865328	6.0330116163	-1.2502217239
H	4.7176946044	5.31433919	-2.7350959829
H	1.5325005442	4.7249583041	-0.9093580803
H	3.299340113	5.9310269757	-4.6122501843
H	1.0987141784	5.584903066	-5.7265744739
H	-6.111190285	2.3255652111	-1.2653385987
H	-4.9748051007	0.952003391	-1.3866989222
H	-5.0464670668	1.9306225314	0.1141028236
H	5.4501138507	1.0275110575	2.0591085927
H	2.0442338835	2.5248961227	2.0456237855
H	3.6803275955	2.6568198233	2.7035887735
H	3.2293079124	0.9057754451	4.373261549
H	0.5002672818	0.730870397	2.9982718991
H	1.6735045375	-0.604298606	5.5543035947
H	0.0135532063	-2.2372226845	5.4332825343
H	-2.0365054427	-1.6428785358	4.1641181245
H	-1.1147742487	-0.8277682273	2.9178145736
H	-2.0754163644	-3.2803660348	2.5652064152
H	-0.7015699782	-3.8061851506	3.5108565184
H	1.1736066277	-1.5360404554	2.2000032198
H	-1.3781428316	-4.5512554944	0.5419655769
H	0.0485707144	-4.5937996537	-1.4835356252
H	-3.579901611	4.4987543078	-2.5870893294
H	-1.7396107839	4.496575544	-4.1777564362
H	6.9829910979	-0.6138832029	1.2534212979
H	5.3801590807	-2.9284256136	-0.0663513899
H	3.0421052701	-2.9206093243	-1.7495348111

	shielding constants	calcd. δ_C[a]
C-1	43.62	136.42
C-2	58.14	122.64
C-3	39.83	140.02
C-4	42.08	137.88
C-5	82.12	99.87
C-6	35.62	144.01
C-7	52.13	128.34
C-8	27.79	151.45
C-9	125.58	58.61
C-10	50.25	130.13
C-11	46.46	133.73
C-12	156.47	29.28
C-13	148.99	36.38
C-14	26.87	152.32
C-15	58.36	122.43
C-16	46.32	133.86
C-17	49.50	130.84
C-18	52.24	128.24
C-19	65.80	115.37
6-OMe	131.44	53.05
C-1'	41.40	138.53
C-2'	49.50	130.84
C-3'	48.54	131.75
C-4'	43.38	136.65
C-5'	71.16	110.28
C-6'	42.11	137.86
C-7'	146.74	38.52
C-8'	41.21	138.71
C-9'	54.22	126.35
C-10'	52.16	128.31
C-11'	46.97	133.24
C-12'	156.30	29.44
C-13'	153.15	32.43
C-14'	28.37	150.90
C-15'	56.94	123.78
C-16'	42.40	137.58
C-17'	48.34	131.94
C-18'	52.05	128.42
C-19'	65.74	115.42
H-5	24.70	6.44
H-7	24.76	6.38
H-9	26.27	5.00
H-10	27.17	4.18
H-11	26.46	4.83
H-12	28.69	2.79
H-12	29.42	2.13
H-13	28.17	3.27
H-13	29.01	2.51
H-16	23.54	7.49
H-18	24.47	6.65
H-19	24.56	6.57
H-5'	24.82	6.32
H-7'	27.64	3.75
H-7'	28.45	3.02
H-8'	25.57	5.65
H-9'	24.21	6.88
H-10'	25.43	5.77
H-11'	25.85	5.39
H-12'	29.04	2.47
H-12'	28.61	2.87
H-13'	28.63	2.85
H-13'	28.81	2.68
H-16'	24.55	6.57
H-18'	24.21	6.88
H-19'	24.42	6.69
6-OMe	27.48	3.71

Table S8. Experimental chemical shifts, calculated isotropic shielding constants, and predicted chemical shifts of zosterabisphenone A (**1**). Calculations were performed at the PBE0/6-311+G(2d,p)/PCM($CHCl_3$)//B3LYP/6-31+G(d,p) level.

	exp. δ_C	shielding constants	calcd. δ_C[a]	$\Delta\delta_C$
C-1	138.0	42.81	137.19	-0.81
C-2	122.4	57.71	123.04	0.64
C-3	139.7	39.94	139.92	0.22
C-4	137.3	44.03	136.03	-1.27
C-5	103.0	81.52	100.44	-2.56
C-6	145.9	35.51	144.12	-1.78
C-7	128.8	49.92	130.44	1.64
C-8	148.3	29.57	149.76	1.46
C-9	51.3	130.85	53.61	2.31
C-10	127.4	50.49	129.90	2.50
C-11	134.4	42.66	137.33	2.93
C-12	27.6	156.11	29.62	2.02
C-13	34.1	148.96	36.41	2.31
C-14	152.0	26.82	152.37	0.37
C-15	122.7	58.19	122.59	-0.11
C-16	135.1	45.91	134.25	-0.85
C-17	131.9	49.50	130.84	-1.06
C-18	129.7	51.97	128.50	-1.20
C-19	116.7	65.62	115.54	-1.16
6-OMe	56.2	131.18	53.29	-2.91
C-1'	103.9	78.54	103.27	-0.63
C-2'	50.2	131.30	53.18	2.98
C-3'	46.1	136.09	48.63	2.53
C-4'	163.3	12.38	166.08	2.78
C-5'	122.6	57.60	123.15	0.55
C-6'	189.1	-10.01	187.34	-1.76
C-7'	38.5	145.33	39.86	1.36
C-8'	124.5	55.17	125.45	0.95
C-9'	128.1	51.10	129.32	1.22
C-10'	129.2	50.61	129.79	0.59
C-11'	129.4	48.65	131.64	2.24
C-12'	29.4	154.32	31.32	1.92
C-13'	33.6	150.20	35.24	1.64
C-14'	154.9	24.80	154.29	-0.61
C-15'	127.8	52.50	127.99	0.19
C-16'	123.5	57.20	123.53	0.03
C-17'	133.9	47.39	132.84	-1.06
C-18'	130.5	51.10	129.32	-1.18
C-19'	109.0	73.95	107.62	-1.38
			**RMSD ^{13}C**	**1.66**

	exp. δ_H	shielding constants	calcd. δ_H[b]	$\Delta\delta_H$
H-5	6.80	24.56	6.56	-0.24
H-7	6.25	25.00	6.16	-0.09
H-9	5.01	26.35	4.93	-0.08
H-10	4.76	26.50	4.80	0.04
H-11	5.55	25.58	5.63	0.08
H-12	3.05	28.39	3.07	0.02
H-12	2.56	28.95	2.56	0.00
H-13	3.39	28.03	3.40	0.01
H-13	2.71	28.79	2.70	-0.01
H-16	7.68	23.52	7.51	-0.17
H-18	6.91	24.34	6.77	-0.14
H-19	6.86	24.46	6.66	-0.20
H-2'	3.85	27.69	3.71	-0.14
H-3'	3.56	27.84	3.57	0.01
H-5'	6.18	25.14	6.04	-0.14
H-7'	2.33	29.17	2.36	0.03
H-7'	2.97	28.56	2.91	-0.06
H-8'	5.08	26.31	4.97	-0.11
H-9'	5.89	25.19	5.99	0.10
H-10'	5.65	25.56	5.65	0.00
H-11'	5.22	26.05	5.21	-0.01
H-12'	2.31	29.28	2.25	-0.06
H-12'	2.53	28.91	2.59	0.06
H-13'	2.98	28.51	2.96	-0.02
H-13'	2.34	29.26	2.28	-0.06
H-16'	6.85	24.22	6.88	0.03
H-18'	6.70	24.50	6.61	-0.09
H-19'	6.56	24.85	6.30	-0.26
		27.45		
6-OMe	3.96	27.73	3.75	-0.21
		27.76		
			**RMSD ^{1}H**	**0.113**

[a] ^{13}C chemical shifts were obtained from the isotropic shielding constants according to ref. 10, using the equation: $\delta = (187.3123 - \text{shielding})/1.0533$
[b] ^{1}H chemical shifts were obtained from the isotropic shielding constants according to ref. 10, using the equation: $\delta = (31.7532 - \text{shielding})/1.0958$

Table S9. Experimental chemical shifts, calculated isotropic shielding constants, and predicted chemical shifts of the two conformers of the C-9 epimer of zosterabisphenone A (*epi*-**1**). Calculations were performed at the PBE0/6-311+G(2d,p)/PCM($CHCl_3$)//B3LYP/6-31+G(d,p) level.

		isotropic shieldings				
	exp. δ_C	Conformer 1 ΔE = 0.00 kcal/mol population 77.6%	Conformer 2 ΔE = 0.63 kcal/mol population 22.4%	average	calcd. δ_C[a]	$\Delta\delta_C$
C-1	138.0	42.70	43.13	42.79	137.21	-0.79
C-2	122.4	57.93	57.73	57.89	122.87	0.47
C-3	139.7	40.93	41.34	41.02	138.89	-0.81
C-4	137.3	43.27	42.77	43.16	136.86	-0.44
C-5	103.0	81.57	81.64	81.58	100.38	-2.62
C-6	145.9	35.45	35.50	35.46	144.17	-1.73
C-7	128.8	48.72	50.62	49.15	131.17	2.37
C-8	148.3	30.86	24.60	29.46	149.87	1.57
C-9	51.3	126.00	129.25	126.72	57.52	6.22
C-10	127.4	51.65	50.26	51.34	129.09	1.69
C-11	134.4	43.14	43.49	43.22	136.80	2.40
C-12	27.6	156.58	155.52	156.35	29.40	1.80
C-13	34.1	148.73	149.14	148.82	36.54	2.44
C-14	152.0	26.61	26.71	26.63	152.55	0.55
C-15	122.7	58.34	58.20	58.31	122.48	-0.22
C-16	135.1	46.10	46.22	46.13	134.04	-1.06
C-17	131.9	49.74	50.11	49.82	130.53	-1.37
C-18	129.7	51.88	52.26	51.97	128.50	-1.20
C-19	116.7	65.77	65.46	65.70	115.46	-1.24
6-OMe	56.2	131.21	131.18	131.20	53.27	-2.93
C-1'	103.9	78.70	78.26	78.60	103.21	-0.69
C-2'	50.2	131.84	131.82	131.83	52.67	2.47
C-3'	46.1	135.75	137.29	136.09	48.63	2.53
C-4'	163.3	11.98	7.98	11.08	167.31	4.01
C-5'	122.6	58.91	59.33	59.00	121.82	-0.78
C-6'	189.1	-10.55	-9.59	-10.34	187.65	-1.45
C-7'	38.5	145.32	145.23	145.30	39.89	1.39
C-8'	124.5	55.40	56.01	55.54	125.11	0.61
C-9'	128.1	51.39	50.87	51.27	129.15	1.05
C-10'	129.2	50.57	50.60	50.58	129.81	0.61
C-11'	129.4	48.41	48.38	48.40	131.88	2.48
C-12'	29.4	154.27	154.25	154.26	31.38	1.98
C-13'	33.6	150.39	150.37	150.38	35.06	1.46
C-14'	154.9	25.01	25.21	25.05	154.05	-0.85
C-15'	127.8	52.44	52.43	52.44	128.05	0.25
C-16'	123.5	57.55	57.40	57.52	123.23	-0.27
C-17'	133.9	47.81	47.51	47.74	132.51	-1.39
C-18'	130.5	51.38	51.08	51.31	129.12	-1.38
C-19'	109.0	73.84	74.04	73.88	107.69	-1.31
					**RMSD ^{13}C**	**1.93**

	exp. δ_H	Conformer 1	Conformer 2	average	calcd. δ_H[b]	$\Delta\delta_H$
H-5	6.80	24.56	24.50	24.54	6.58	-0.22
H-7	6.25	24.92	24.27	24.77	6.37	0.12
H-9	5.01	26.51	26.64	26.54	4.76	-0.25
H-10	4.76	26.39	26.45	26.40	4.88	0.12
H-11	5.55	25.66	25.65	25.66	5.56	0.01
H-12	3.05	28.32	28.49	28.36	3.10	0.05
H-12	2.56	28.84	29.07	28.89	2.61	0.05
H-13	3.39	27.98	28.07	28.00	3.42	0.03
H-13	2.71	28.75	28.83	28.77	2.72	0.01
H-16	7.68	23.49	23.60	23.51	7.52	-0.16
H-18	6.91	24.31	24.36	24.33	6.78	-0.13
H-19	6.86	24.43	24.45	24.44	6.68	-0.18
H-2'	3.85	27.44	27.50	27.45	3.93	0.08
H-3'	3.56	27.98	27.90	27.96	3.46	-0.10
H-5'	6.18	25.20	25.46	25.26	5.92	-0.26
H-7'	2.33	29.02	28.63	28.93	2.58	0.25
H-7'	2.97	28.55	28.51	28.54	2.93	-0.04
H-8'	5.08	26.30	26.38	26.32	4.96	-0.12
H-9'	5.89	25.09	25.20	25.11	6.06	0.17
H-10'	5.65	25.53	25.56	25.54	5.67	0.02
H-11'	5.22	26.02	26.03	26.02	5.23	0.01
H-12'	2.31	29.23	29.29	29.24	2.29	-0.02
H-12'	2.53	28.87	28.96	28.89	2.61	0.08
H-13'	2.98	28.51	28.55	28.52	2.95	-0.03
H-13'	2.34	29.24	29.26	29.24	2.29	-0.05
H-16'	6.85	24.21	24.31	24.23	6.87	0.02
H-18'	6.70	24.61	24.56	24.60	6.53	-0.17
H-19'	6.56	24.90	24.85	24.89	6.27	-0.29
		27.77	27.44			
6-OMe	3.96	27.76	27.74	27.76	3.65	-0.31
		27.85	27.71			
					**RMSD ^{1}H**	**0.148**

[a] ^{13}C chemical shifts were obtained from the isotropic shielding constants according to ref. 10, using the equation: δ = (187.3123 − shielding)/1.0533
[b] ^{1}H chemical shifts were obtained from the isotropic shielding constants according to ref. 10, using the equation: δ = (31.7532 − shielding)/1.0958

Table S10. Experimental chemical shifts, calculated isotropic shielding constants, and predicted chemical shifts of the two conformers of zosterabisphenone B (**2**). Calculations were performed at the PBE0/6-311+G(2d,p)/PCM($CHCl_3$)//B3LYP/6-31+G(d,p) level.

		isotropic shieldings				
	exp. δ_C	Conformer 1 ΔE = 0.00 kcal/mol population 85.4%	Conformer 2 ΔE = 0.91 kcal/mol Population 14.6%	average	calcd. δ_C[a]	$\Delta\delta_C$
C-1	138.0	42.58	42.69	42.59	138.05	0.06
C-2	122.4	57.65	57.78	57.67	123.63	1.23
C-3	139.6	39.92	40.39	39.99	140.55	0.94
C-4	136.8	44.59	43.62	44.45	136.28	-0.54
C-5	102.6	81.95	81.79	81.93	100.40	-2.22
C-6	145.6	35.70	35.60	35.68	144.67	-0.96
C-7	134.7	43.40	51.53	44.58	136.15	1.47
C-8	141.1	35.82	31.12	35.14	145.19	4.10
C-9	50.6	130.93	127.76	130.47	53.94	3.37
C-10	126.3	51.74	53.12	51.94	129.11	2.79
C-11	132.2	45.70	42.25	45.20	135.56	3.35
C-12	27.6	156.40	156.11	156.36	29.16	1.53
C-13	33.9	149.54	149.34	149.51	35.72	1.87
C-14	151.5	27.19	26.83	27.14	152.85	1.31
C-15	122.3	58.41	58.39	58.41	122.92	0.59
C-16	134.9	46.22	46.35	46.24	134.57	-0.32
C-17	132.7	49.04	49.86	49.16	131.77	-0.89
C-18	129.7	52.01	51.86	51.99	129.06	-0.59
C-19	116.6	65.36	65.61	65.40	116.22	-0.36
6-OMe	56.1	131.31	131.36	131.32	53.13	-2.98
C-1'	113.6	66.09	65.62	66.02	115.63	2.01
C-2'	175.4	-0.41	-0.48	-0.42	179.23	3.79
C-3'	42.3	139.66	142.95	140.14	44.69	2.40
C-4'	121.6	57.38	51.99	56.59	124.66	3.03
C-5'	145.3	33.57	35.47	33.84	146.43	1.10
C-6'	183.2	-2.43	-2.62	-2.46	181.18	-2.03
C-7'	27.6	155.60	154.82	155.49	29.99	2.36
C-8'	129.4	47.76	48.62	47.88	132.99	3.61
C-9'	128.0	52.30	52.71	52.36	128.70	0.72
C-10'	128.4	51.90	52.05	51.92	129.13	0.70
C-11'	129.5	48.79	48.57	48.75	132.16	2.64
C-12'	27.9	155.80	157.50	156.05	29.45	1.59
C-13'	24.5	160.00	160.07	160.01	25.66	1.20
C-14'	144.7	34.67	35.42	34.78	145.54	0.85
C-15'	136.4	44.52	44.45	44.51	136.22	-0.13
C-16'	119.1	62.65	64.85	62.97	118.55	-0.55
C-17'	121.7	60.34	61.04	60.44	120.97	-0.72
C-18'	154.9	25.43	25.82	25.49	154.43	-0.43
C-19'	99.4	86.14	86.07	86.13	96.38	-2.98
18'-OMe	55.8	132.04	132.09	132.05	52.43	-3.41
					**RMSD ^{13}C**	**2.05**

	exp. δ_H	Conformer 1	Conformer 2	average	calcd. δ_H[b]	$\Delta\delta_H$
H-5	6.85	24.57	24.56	24.56	6.67	-0.18
H-7	6.26	24.92	24.57	24.87	6.39	0.13
H-9	4.76	26.61	26.68	26.62	4.78	0.02
H-10	4.36	26.93	26.74	26.90	4.52	0.16
H-11	4.93	26.24	26.37	26.26	5.11	0.18
H-12	2.72	28.77	28.84	28.78	2.79	0.07
H-12	2.17	29.41	29.44	29.41	2.20	0.03
H-13	2.53	29.00	29.00	29.00	2.59	0.06
H-13	3.26	28.20	28.20	28.20	3.32	0.06
H-16	7.53	23.65	23.64	23.65	7.52	-0.01
H-18	6.81	24.46	24.44	24.46	6.77	-0.04
H-19	6.77	24.52	24.48	24.52	6.72	-0.05
H-1'	6.34	25.06	25.03	25.06	6.22	-0.12
H-3'	4.62	26.88	27.18	26.92	4.50	-0.12
H-7'	3.41	27.93	27.64	27.89	3.61	0.20
H-7'	2.35	29.00	28.76	28.96	2.62	0.27
H-8'	5.60	25.64	25.53	25.62	5.70	0.10
H-9'	5.86	25.31	25.23	25.30	6.00	0.14
H-10'	5.90	25.35	25.32	25.34	5.96	0.06
H-11'	5.34	25.92	25.92	25.92	5.42	0.08
H-12'	2.65	28.81	28.77	28.80	2.76	0.11
H-12'	2.28	29.32	29.37	29.32	2.28	0.00
H-13'	3.01	28.45	28.49	28.45	3.09	0.08
H-13'	2.43	29.14	29.18	29.14	2.45	0.02
H-16'	6.72	24.50	24.56	24.50	6.73	0.01
H-19'	6.50	25.03	25.19	25.05	6.22	-0.28
		27.43	27.48			
6-OMe	3.97	27.74	27.79	27.64	3.83	-0.14
		27.74	27.75			
		27.62	27.69			
18'-OMe	3.77	28.01	28.14	27.89	3.60	-0.17
		28.01	28.09			
					**RMSD ^{1}H**	**0.126**

[a] ^{13}C chemical shifts were obtained from the isotropic shielding constants according to ref. 10, using the equation: δ = (187.3123 − shielding)/1.0533
[b] ^{1}H chemical shifts were obtained from the isotropic shielding constants according to ref. 10, using the equation: δ = (31.7532 − shielding)/1.0958

Table S11. Experimental chemical shifts, calculated isotropic shielding constants, and predicted chemical shifts of the two conformers of the C-9 epimer of zosterabisphenone B (*epi*-**2**). Calculations were performed at the PBE0/6-311+G(2d,p)/PCM($CHCl_3$)//B3LYP/6-31+G(d,p) level.

		isotropic shieldings				
	exp. δ_C	Conformer 1 ΔE = 0.00 kcal/mol population 84.5%	Conformer 2 ΔE = 0.87 kcal/mol population 25.5%	average	calcd. δ_C[a]	$\Delta\delta_C$
C-1	138.0	42.55	42.74	42.58	138.07	0.08
C-2	122.4	57.63	57.54	57.62	123.67	1.27
C-3	139.6	39.74	40.52	39.86	140.67	1.06
C-4	136.8	44.47	43.37	44.30	136.42	-0.40
C-5	102.6	82.04	81.82	82.01	100.33	-2.29
C-6	145.6	35.67	35.48	35.64	144.71	-0.92
C-7	134.7	43.34	49.63	44.31	136.41	1.73
C-8	141.1	37.19	32.38	36.44	143.94	2.85
C-9	50.6	130.85	127.55	130.33	54.07	3.50
C-10	126.3	52.34	54.12	52.61	128.46	2.14
C-11	132.2	43.51	41.57	43.21	137.47	5.26
C-12	27.6	156.53	156.48	156.53	29.00	1.37
C-13	33.9	149.39	149.14	149.35	35.87	2.02
C-14	151.5	27.08	26.86	27.04	152.94	1.40
C-15	122.3	58.86	58.52	58.81	122.53	0.20
C-16	134.9	46.09	45.78	46.04	134.76	-0.13
C-17	132.7	49.13	49.32	49.16	131.77	-0.89
C-18	129.7	52.17	52.13	52.16	128.90	-0.75
C-19	116.6	65.74	65.74	65.74	115.90	-0.68
6-OMe	56.1	131.26	131.21	131.25	53.19	-2.92
C-1'	113.6	64.77	67.02	65.12	116.49	2.87
C-2'	175.4	1.95	-2.25	1.30	177.58	2.14
C-3'	42.3	140.42	142.81	140.79	44.06	1.77
C-4'	121.6	56.05	51.84	55.39	125.80	4.17
C-5'	145.3	33.79	35.34	34.03	146.25	0.92
C-6'	183.2	-2.45	-3.02	-2.54	181.26	-1.95
C-7'	27.6	155.45	155.25	155.42	30.06	2.43
C-8'	129.4	48.06	47.17	47.92	132.95	3.57
C-9'	128.0	52.32	53.52	52.51	128.57	0.59
C-10'	128.4	52.07	51.69	52.01	129.04	0.61
C-11'	129.5	48.52	49.45	48.67	132.24	2.72
C-12'	27.9	155.80	157.49	156.06	29.44	1.58
C-13'	24.5	160.07	159.89	160.05	25.63	1.17
C-14'	144.7	34.94	35.72	35.06	145.26	0.57
C-15'	136.4	44.04	44.13	44.05	136.66	0.31
C-16'	119.1	63.02	64.91	63.32	118.22	-0.88
C-17'	121.7	60.44	60.32	60.42	120.99	-0.70
C-18'	154.9	25.59	25.84	25.63	154.29	-0.57
C-19'	99.4	86.06	85.54	85.98	96.53	-2.83
18'-OMe	55.8	132.02	131.91	132.01	52.47	-3.37
					**RMSD ^{13}C**	**2.08**

	exp. δ_H	Conformer 1	Conformer 2	average	calcd. δ_H[b]	$\Delta\delta_H$
H-5	6.85	24.55	24.55	24.55	6.69	-0.16
H-7	6.26	24.91	24.66	24.87	6.39	0.13
H-9	4.76	26.82	26.72	26.81	4.61	-0.15
H-10	4.36	26.81	26.94	26.83	4.58	0.22
H-11	4.93	26.23	26.14	26.22	5.15	0.22
H-12	2.72	28.90	28.91	28.90	2.67	-0.05
H-12	2.17	29.52	29.83	29.57	2.05	-0.12
H-13	2.53	28.25	28.15	28.24	3.29	0.03
H-13	3.26	29.02	29.03	29.02	2.56	0.03
H-16	7.53	23.74	23.57	23.71	7.46	-0.07
H-18	6.81	24.44	24.43	24.44	6.79	-0.02
H-19	6.77	24.51	24.49	24.51	6.72	-0.05
H-1'	6.34	25.17	25.18	25.17	6.12	-0.22
H-3'	4.62	26.86	27.37	26.94	4.49	-0.13
H-7'	3.41	27.69	27.87	27.72	3.76	0.35
H-7'	2.35	28.92	29.10	28.95	2.63	0.28
H-8'	5.60	25.65	25.77	25.67	5.66	0.06
H-9'	5.86	25.28	25.25	25.27	6.02	0.16
H-10'	5.90	25.36	25.41	25.37	5.93	0.03
H-11'	5.34	25.92	25.98	25.93	5.42	0.08
H-12'	2.65	28.80	28.76	28.80	2.77	0.12
H-12'	2.28	29.31	29.37	29.32	2.29	0.01
H-13'	3.01	28.44	28.45	28.44	3.10	0.09
H-13'	2.43	29.14	29.14	29.14	2.45	0.02
H-16'	6.72	24.49	24.53	24.50	6.74	0.02
H-19'	6.50	24.97	24.83	24.95	6.32	-0.18
		27.43	27.43			
6-OMe	3.97	27.74	27.77	27.65	3.83	-0.14
		27.77	27.76			
		27.62	27.58			
18'-OMe	3.77	27.99	27.96	27.86	3.63	-0.14
		27.99	27.95			
					**RMSD ^{1}H**	**0.145**

[a] ^{13}C chemical shifts were obtained from the isotropic shielding constants according to ref. 10, using the equation: δ = (187.3123 − shielding)/1.0533
[b] ^{1}H chemical shifts were obtained from the isotropic shielding constants according to ref. 10, using the equation: δ = (31.7532 − shielding)/1.0958

Table S12. Experimental multiplicity of ^{1}H NMR signals and predicted ^{1}H-^{1}H *J* couplings (Hz) of zosterabisphenone A (**1**). Predicted couplings whose magnitude is smaller than 0.2 Hz are not reported.

southern unit		predicted ^{1}H-^{1}H *J* couplings (Hz)												
		H-5	H-7	H-9	H-10	H-11	H-12 proR	H-12 proS	H-13 proR	H-13 proS	H-16	H-18	H-19	H-3'
Position	δ_H, mult (*J* in Hz)													
H-5	6.80, s		-0.3											-0.2
H-7	6.25, s	-0.3		-2.1										-1.4
H-9	5.01, br. d (11.2)		-2.1		10.8	-0.8	-0.7				-0.4			-0.5
H-10	4.76, t (11.2)			10.8		11.4	-0.9	-1.7		0.5				
H-11	5.55, ddd (11.8, 11.2, 4.8)			-0.8	11.4		12.1	5.1		-0.4				
H-12 proR	3.05, dddd (13.6, 12.6, 11.8, 7.0)			-0.7	-0.9	12.1		-13.4	11.8	7.2				
H-12 proS	2.56, overlapped				-1.7	5.1	-13.4		6.0	1.0				
H-13 proR	2.71, ddd (14.4, 12.6, 6.0)						11.8	6.0		-14.4		-0.4		
H-13 proS	3.39, dd (14.4, 7.0)				0.5	-0.4	7.2	1.0	-14.4		-1.2	-1.1	0.6	
H-16	7.68, br. s			-0.4						-1.2		2.0	0.4	
H-18	6.91, br. d (8.0)								-0.4	-1.1	2.0		8.1	
H-19	6.86, d (8.0)									0.6	0.4	8.1		

northern unit		predicted ^{1}H-^{1}H *J* couplings (Hz)															
		H-2'	H-3'	H-5'	H-7' proR	H-7' proS	H-8'	H-9'	H-10'	H-11'	H-12' proR	H-12' proS	H-13' proR	H-13' proS	H-16'	H-18'	H-19'
Position	δ_H, mult (*J* in Hz)																
H-2'	3.85, s		1.1										-0.4		-1.2	-0.9	0.5
H-3'	3.56, s	1.1			0.4	-0.4									-0.2		
H-5'	6.18, s				-1.1	-0.5											
H-7' proR	2.97, dd (12.4, 7.4)		0.4	-1.1		-11.8	7.5	-1.0	0.5	-0.5	0.5						
H-7' proS	2.33, overlapped		-0.4	-0.5	-11.8		9.1	-1.1	0.3	-0.4	0.4						
H-8'	5.08, ddd (15.3, 8.0, 8.0)				7.5	9.1		15.6	-1.0	1.0	-0.8						
H-9'	5.89, dd (15.3, 10.6)				-1.0	-1.1	15.6		10.7	-1.4	0.6	-0.4					
H-10'	5.65, t (10.6)				0.5	0.3	-1.0	10.7		11.5	-1.6	-0.9	0.3				
H-11'	5.22, ddd (11.5, 10.6, 5.3)				-0.5	-0.4	1.0	-1.4	11.5		5.7	11.8	-0.2	-0.2			
H-12' proR	2.31, overlapped				0.5	0.4	-0.8	0.6	-1.6	5.7		-12.8	3.5	3.4			
H-12' proS	2.53, dq (3.3,12.2)							-0.4	-0.9	11.8	-12.8		3.9	13.0			
H-13' proR	2.98, overlapped	-0.4							0.3	-0.2	3.5	3.9		-13.5	-0.9	-0.8	0.5
H-13' proR	2.34, overlapped									-0.2	3.4	13.0	-13.5			-0.4	
H-16'	6.85, br. s	-1.2	-0.2										-0.9	-		1.7	0.5
H-18'	6.70, br. d (8.0)	-0.9											-0.8	-0.4	1.7		8.1
H-19'	6.56, d (8.0)	0.5											0.5		0.5	8.1	

Table S13. Experimental multiplicity of ^{1}H NMR signals and predicted ^{1}H-^{1}H J couplings of zosterabisphenone B (**2**). Predicted couplings whose magnitude is smaller than 0.2 Hz are not reported.

southern unit		predicted ^{1}H-^{1}H J couplings (Hz)												
		H-5	H-7	H-9	H-10	H-11	H-12 proR	H-12 proS	H-13 proR	H-13 proS	H-16	H-18	H-19	H-3'
Position	δ_H, mult (J in Hz)													
H-5	6.85, s		-0.3											
H-7	6.26, s	-0.3		-2.2										-0.5
H-9	4.76, br. d (11)		-2.2		10.4	-0.9	-0.7				-0.4			-0.5
H-10	4.36, t (11)			10.4		11.5	-0.9	-1.8		0.4				
H-11	4.93, ddd (11, 11, 5)			-0.9	11.5		12.2	5.1	-0.2	-0.5				
H-12 proR	2.72, m			-0.7	-0.9	12.2		-13.5	11.8	7.2				
H-12 proS	2.17, m				-1.8	5.1	-13.5		6.0	1.0				
H-13 proR	2.53, ddd (13, 13, 6)					-0.2	11.8	6.0		-14.4		-0.4		
H-13 proS	3.26, dd (13, 6)				0.4	-0.5	7.2	1.0	-14.4		-1.2	-1.1	0.6	
H-16	7.53, br. s			-0.4						-1.2		2.0	0.4	
H-18	6.81, br. d (8)								-0.4	-1.1	2.0		8.1	
H-19	6.77, d (8)									0.6	0.4	8.1		

northern unit		predicted ^{1}H-^{1}H J couplings (Hz)													
		H-1'	H-3'	H-7' proR	H-7' proS	H-8'	H-9'	H-10'	H-11'	H-12' proR	H-12' proS	H-13' proR	H-13' proS	H-16'	H-19'
Position	δ_H, mult (J in Hz)														
H-1'	6.34, s														
H-3'	4.62, s			-1.6	-0.3										
H-7' proR	2.35, dd (19, 6)		-1.6		-19.7	7.0	-1.6	0.4	-0.8	1.0					
H-7' proS	3.41, br. d (19)		-0.3	-19.7		3.1	-3.4	1.1	-2.2	2.4		0.2			
H-8'	5.60, br. dd (15, 6)			7.0	3.1		15.8	-0.8	1.1	-1.2					
H-9'	5.86, m			-1.6	-3.4	15.8		11.6	-1.2	0.6	-0.5				
H-10'	5.90, m			0.4	1.1	-0.8	11.6		11.2	-2.1	-0.7	0.3			
H-11'	5.34, ddd (11, 11, 5)			-0.8	-2.2	1.1	-1.2	11.2		5.6	11.8	-0.2	-0.2		
H-12' proR	2.28, m			1.0	2.4	-1.2	0.6	-2.1	5.6		-13.9	5.0	2.5		
H-12' proS	2.65, br. quartet (13)						-0.5	-0.7	11.8	-13.9		2.7	13.4	-0.3	
H-13' proR	2.43, br. d (14)				0.2			0.3	-0.2	5.0	2.7		-14.8	-1.2	0.6
H-13' proS	3.01, br. t (14)								-0.2	2.5	13.4	-14.8			
H-16'	6.72, s										-0.3	-1.2			0.3
H-19'	6.50, s											0.6		0.3	

Table S14. Rotatory strengths (length formalism) calculated for zosterabisphenone A (**1**). Calculations were performed at the ωB97XD/6-31+G(d,p)//B3LYP/6-31+G(d,p) level.

Wavelength (nm)	Rotatory strength (10^{-40} erg esu cm Gauss^{-1})	Wavelength (nm)	Rotatory strength (10^{-40} erg esu cm Gauss^{-1})
154.2	-5.772300	172.4	12.977700
154.3	1.541200	173.3	28.706500
154.5	3.893400	174.0	-4.519900
154.6	-10.440700	174.4	-27.216800
154.7	-7.697300	175.2	-15.858000
154.8	7.970900	175.8	24.097800
155.2	-31.635200	176.1	59.054500
155.3	12.115700	176.5	67.683100
155.6	-5.555600	176.7	13.526000
156.1	-4.629800	177.1	-0.621700
156.3	18.611000	177.4	-21.616900
156.6	-13.818100	177.7	35.119500
156.7	5.712400	178.0	-36.729400
156.9	1.595000	178.6	28.256100
157.0	-3.321700	179.0	-13.432700
157.3	27.079500	179.6	-3.024600
157.5	22.680600	179.7	-30.905200
157.6	28.708000	180.0	34.459000
158.1	5.242200	180.2	41.971900
158.3	-10.164400	180.9	-5.846900
158.5	-0.567000	181.1	37.356200
159.0	-52.780000	181.4	-4.326600
159.1	-3.250000	181.5	-85.923100
159.2	13.062600	182.9	-6.718500
159.5	12.140200	183.8	-44.258000
159.7	34.046700	184.6	147.774000
159.8	-1.469900	184.8	-40.092300
160.0	10.957300	185.5	-71.196700
160.1	-0.695600	186.0	-19.141000
160.3	8.561600	187.4	92.584100
160.4	-16.036500	187.8	-5.689900
160.8	-55.483000	188.5	12.489100
160.9	-1.154500	188.8	124.362100
161.2	-0.585200	189.3	15.474100
161.5	-27.228300	190.1	32.238800
161.6	4.859500	191.2	-100.354400
161.9	2.391500	192.2	-106.711400
162.2	-52.850900	192.3	-92.401300
162.4	-1.034100	193.8	-45.835500
162.7	5.868300	194.2	7.917400
163.0	7.152700	195.3	-19.843200
163.2	1.120300	195.5	-19.959400
163.5	-0.615500	196.1	45.606000
163.7	-26.574100	197.1	42.327800
164.1	-67.908300	198.2	69.862700
164.4	14.053000	198.4	18.801200
164.6	-23.579100	200.5	52.275300
165.0	15.012400	200.9	86.801900
165.3	-14.487900	202.1	-78.328000
165.7	36.021900	202.7	-5.918400
165.9	19.796600	203.9	223.407200
166.2	56.541300	206.0	-263.886900
166.3	-46.183900	206.4	12.806700
166.6	-1.189200	206.8	-73.070300
166.7	-54.545500	209.0	-26.396900
166.9	-5.216300	210.4	-59.320200
167.2	33.239600	215.1	-64.030700
167.5	8.547700	216.5	14.225400
167.8	-6.360400	217.2	-159.651700
168.2	8.413500	218.5	-24.128500
168.6	-3.269600	219.7	36.298000
168.7	2.910900	225.1	218.617000
169.1	-2.461300	226.8	189.465800
169.4	-39.800000	231.6	-291.876800
169.5	-24.449300	239.3	35.972300
169.7	2.466500	253.4	40.881000
170.0	-8.438600	264.0	-188.955400
170.6	-1.489600	264.7	47.877300
171.3	11.400800	270.4	109.088900
171.4	33.352500	272.9	-100.810700
171.5	-0.981200	275.0	-8.082200
172.0	-3.698200	285.8	3.111100
172.2	-1.949900	315.4	30.210900

Table S15. Rotatory strengths (length formalism) calculated for the two conformers of zosterabisphenone B (**2**). Calculations were performed at the ωB97XD/6-31+G(d,p)//B3LYP/6-31+G(d,p) level.

Conformer 1, Δ*E* = 0.00 kcal/mol, population 85.4%		Conformer 2, Δ*E* = 0.91 kcal/mol, population 14.6%	
Wavelength (nm)	Rotatory strength (10^{-40} erg esu cm $Gauss^{-1}$)	Wavelength (nm)	Rotatory strength (10^{-40} erg esu cm $Gauss^{-1}$)
157.4	10.695000	157.5	7.422900
157.5	7.878900	157.7	23.052500
157.7	30.015100	157.8	-7.352800
157.9	-3.903700	158.0	-8.935200
158.1	11.341500	158.2	20.811200
158.2	-6.628300	158.5	-20.022900
158.3	1.738600	158.8	-2.355600
158.4	-2.726700	159.0	13.069900
158.6	6.436900	159.1	-36.318800
158.9	4.174900	159.4	10.940500
159.2	-29.048700	159.5	-20.836100
159.4	-9.213800	159.8	-1.502500
159.7	27.055500	160.1	-18.692300
159.8	26.716500	160.4	-6.776300
160.0	-12.860000	160.5	22.978000
160.3	-0.881700	160.6	7.936000
160.4	-8.400100	160.8	-10.933100
160.5	1.422000	161.0	-1.208600
160.8	0.814900	161.3	-3.707200
161.1	-25.898000	161.6	-9.683800
161.4	-3.591100	162.0	-1.269500
161.6	-23.514400	162.3	13.831800
161.8	15.463200	162.4	-4.985100
161.9	-0.227900	162.5	5.799800
162.0	-9.241400	162.9	-6.869100
162.3	-6.218800	163.0	23.561200
162.5	-9.932300	163.2	-12.109200
162.7	-43.780200	163.3	10.420800
163.1	1.234600	163.5	-20.024700
163.3	-4.189000	163.7	46.345400
163.5	19.068100	164.0	-32.850400
163.7	18.930500	164.2	0.458100
163.8	-6.920200	164.4	-10.645800
164.0	13.454300	164.6	-15.024300
164.4	2.465900	165.0	-7.340500
164.8	8.656900	165.3	5.918500
164.9	-18.239300	165.6	6.680500
165.4	6.317600	165.9	-4.114300
165.6	0.834500	166.1	-0.437000
165.9	9.111500	166.2	-1.458000
166.2	3.212300	166.8	-7.150100
166.4	-13.012500	167.2	-24.239600
166.6	7.802100	167.3	-7.370600
166.8	7.808700	167.5	11.572800
167.0	-44.886300	167.6	-9.866600
167.2	-6.793700	168.1	10.924400
167.5	-8.949700	168.4	-8.839600
167.7	34.076400	168.5	9.612900
168.1	17.703500	168.8	-9.111300
168.3	-24.049300	169.2	-21.623000
168.4	-4.713900	169.3	11.535900
168.9	-14.873200	169.5	17.727300
169.2	-7.410100	169.7	-2.586700
169.8	-2.649200	169.8	2.877600
170.0	34.952300	170.0	-50.819800
170.4	4.109300	170.5	-22.541100
170.6	-33.338600	170.7	25.009300
170.9	27.664900	171.0	-9.545700
171.3	-5.784700	171.4	12.855000
171.4	-15.723300	171.9	-20.441500
171.6	1.438100	172.3	14.726900
171.8	-8.947900	172.4	4.285000
172.2	-0.131700	172.7	3.055100
172.6	-22.696000	172.8	6.293900
173.0	3.991700	173.4	36.684300
173.4	-7.924000	174.1	-11.563700
173.8	0.438800	174.3	-19.232200
174.2	9.996100	174.4	4.563300
174.6	29.170500	174.8	11.591200
174.8	-8.373000	175.1	21.195200
175.2	58.104100	175.7	-13.861400
175.5	-8.174000	175.9	-21.134200
176.4	15.202300	176.1	25.709900
176.8	-19.474500	176.2	43.046500
177.3	0.428600	177.2	-8.758200
177.4	3.756200	177.5	28.682400
177.6	13.496000	177.8	-40.761700
178.2	-12.263900	178.2	-40.775300
179.0	105.205200	178.7	40.186100
179.4	-65.625200	179.0	25.232700
179.8	0.148300	179.2	20.465300

180.4	-53.543800	179.5	-3.996400
180.6	-16.170300	179.7	-29.328800
181.0	-16.126800	179.8	13.125100
181.3	50.062300	179.9	-0.688800
181.5	-34.422100	180.3	47.758900
181.8	16.247400	180.4	-47.865300
182.9	145.109900	181.0	24.699500
183.4	70.388500	181.2	21.442700
183.6	-6.940200	181.4	-4.414000
184.2	-34.444600	182.4	8.346900
184.7	-66.281200	183.0	-9.676700
185.2	-175.027600	183.6	-7.660700
185.5	-2.387500	184.3	59.696200
186.3	76.099000	185.2	-94.917300
186.6	46.920000	185.4	-4.499400
186.8	13.026400	186.6	241.948800
187.2	31.558300	187.2	-155.501700
187.5	71.671100	187.8	-22.002300
188.8	123.496000	188.4	12.366100
189.1	-117.271600	188.7	123.844800
189.5	126.382600	189.0	-52.067100
190.4	-11.592400	189.6	67.812700
192.3	-29.985900	190.1	-61.410100
192.9	64.312900	192.1	28.899900
194.0	-44.472300	193.1	-14.705800
194.5	34.931600	193.5	-122.324700
195.1	-9.721300	194.7	-38.745800
196.0	25.222300	195.0	-67.400900
196.2	-2.360700	195.4	150.982300
197.0	43.157100	197.0	-39.845500
198.1	0.926000	197.8	-39.257900
198.8	-111.100200	198.6	-54.483800
199.8	19.194400	199.6	115.692700
200.9	-9.548400	200.0	188.851600
201.9	-11.150300	201.0	28.754800
202.1	0.291400	201.5	22.303200
203.8	-54.910800	202.4	-28.152500
204.0	-45.061800	204.3	29.945900
205.3	-4.543600	204.9	-8.621600
206.4	9.851500	205.3	-66.458900
207.7	-32.310600	205.6	-45.324000
208.0	-130.152500	207.0	-15.682100
211.0	-21.341500	208.4	26.425000
212.0	-8.066200	208.5	14.180200
212.4	2.229500	210.8	31.262700
214.9	-109.322300	211.7	-248.795100
217.0	-0.993200	211.8	-36.374900
218.2	-74.338500	213.8	-42.555200
219.2	-22.882900	215.7	-123.511200
222.5	-46.927700	217.6	-185.555200
225.8	195.291500	218.1	49.057400
226.5	-136.384100	219.3	-9.291700
230.6	20.305800	224.2	30.399500
232.0	439.674400	225.9	-20.845800
233.7	-126.587000	228.3	2.441500
239.4	-2.924500	232.4	723.136500
244.4	-94.636100	237.3	-363.145000
257.5	-42.293900	239.5	-13.833600
263.9	-10.720800	258.3	-67.448600
268.8	-136.052500	265.6	-11.359900
270.6	109.171600	269.8	-32.713900
277.0	19.384500	273.5	48.933000
278.1	1.933700	276.2	-64.725700
285.2	-119.194000	279.6	50.076700
292.5	18.452100	286.2	-2.397900
319.5	86.494600	294.7	-32.153400
		332.2	81.472400

Table S16. Effects of zosterabisphenone A (**1**) on the viability of HCT116 and Hep G2 cells after 24 hours and 48 hours of treatment. The results are expressed as percentage of cell viability. Each value represents the mean ± SEM of 3 experiments including 5–6 replicates for each treatment. **P<0.0001

Concentration of zosterabisphenone A (**1**) [µM]	HCT116 24 h	HCT116 48 h	Hep G2 24 h	Hep G2 48h
Ctrl	100.0±1.53	100.0±1.53	100.0±1.92	100.0±2.95
0.1	102.0±2.10	97.6±1.47	97.9±2.43	103.0±4.00
0.3	98.6±2.08	95.2±1.83	97.5±2.13	112.0±4.49
1	98.9±2.07	96.0±1.59	102.0±4.16	105.0±2.75
3	98.5±2.25	95.3±2.06	96.7±3.25	114.0±3.56
10	97.6±2.27	77.3±1.41 **	96.7±3.68	111.0±2.35

Table S17. Effects of zosterabisphenone B (**2**) on the viability of HCT116 and Hep G2 cells after 24 hours and 48 hours of treatment. The results are expressed as percentage of cell viability. Each value represents the mean ± SEM of 3 experiments including 5–6 replicates for each treatment. *P<0.01; **P<0.0001

Concentration of zosterabisphenone B (**2**) [µM]	HCT116 24 h	HCT116 48 h	Hep G2 24 h	Hep G2 48h
Ctrl	100.0±2.90	100.0±2.96	100.0±2.52	100.0±2.87
0.1	93.1±2.17	102.0±2.75	96.8±2.27	97.4±3.38
0.3	84.4±2.94 *	79.4±3.30 **	96.5±2.96	91.7±2.04
1	70.2±4.36 **	76.0±3.69 **	91.4±3.79	91.6±3.69
3	67.6±3.75 **	42.9±1.65 **	88.0±2.82	89.0±4.33
10	20.3±2.10 **	2.6±0.65 **	51.4±3.31 **	61.2±6.23 **

References

(1) Alberti, Á.; Riethmüller, E.; Béni, S. Characterization of Diarylheptanoids: An Emerging Class of Bioactive Natural Products. *J. Pharm. Biomed. Anal.* **2018**, *147*, 13–34. https://doi.org/10.1016/j.jpba.2017.08.051.

(2) Smyth, J. E.; Butler, N. M.; Keller, P. A. A Twist of Nature-the Significance of Atropisomers in Biological Systems. *Nat. Prod. Rep.* **2015**, *32* (11), 1562–1583. https://doi.org/10.1039/c4np00121d.

(3) Jahng, Y.; Park, J. G. Recent Studies on Cyclic 1,7-Diarylheptanoids: Their Isolation, Structures, Biological Activities, and Chemical Synthesis. *Molecules* **2018**, *23* (12), 3107. https://doi.org/10.3390/molecules23123107.

(4) Costantino, V.; Fattorusso, E.; Mangoni, A.; Perinu, C.; Teta, R.; Panza, E.; Ianaro, A. Tedarenes A and B: Structural and Stereochemical Analysis of Two New Strained Cyclic Diarylheptanoids from the Marine Sponge Tedania Ignis. *J. Org. Chem.* **2012**, *77* (15), 6377–6383. https://doi.org/10.1021/jo300295j.

(5) Li, Y.; Mangoni, A.; Shulha, O.; Çiçek, S. S.; Zidorn, C. Cyclic Diarylheptanoids Deoxycymodienol and Isotedarene A from Zostera Marina (Zosteraceae). *Tetrahedron Lett.* **2019**, *60* (32), 150930. https://doi.org/10.1016/j.tetlet.2019.07.021.

(6) Grauso, L.; Li, Y.; Scarpato, S.; Shulha, O.; Rárová, L.; Strnad, M.; Teta, R.; Mangoni, A.; Zidorn, C. Structure and Conformation of Zosteraphenols, Tetracyclic Diarylheptanoids from the Seagrass Zostera Marina: An NMR and DFT Study. *Org. Lett.* **2020**, *22* (1), 78–82. https://doi.org/10.1021/acs.orglett.9b03964.

(7) Marcarino, M. O.; Zanardi, M. M.; Cicetti, S.; Sarotti, A. M. NMR Calculations with Quantum Methods: Development of New Tools for Structural Elucidation and Beyond. *Acc. Chem. Res.* **2020**, *53* (9), 1922–1932. https://doi.org/10.1021/acs.accounts.0c00365.

(8) Wolinski, K.; Hinton, J. F.; Pulay, P. Efficient Implementation of the Gauge-Independent Atomic Orbital Method for NMR Chemical Shift Calculations. *J. Am. Chem. Soc.* **1990**, *112* (23), 8251–8260. https://doi.org/10.1021/ja00179a005.

(9) Tomasi, J.; Mennucci, B.; Cammi, R. Quantum Mechanical Continuum Solvation Models. *Chem. Rev.* **2005**, *105* (8), 2999–3094. https://doi.org/10.1021/cr9904009.

(10) Lodewyk, M. W.; Siebert, M. R.; Tantillo, D. J. Computational Prediction of ^{1}H and ^{13}C Chemical Shifts: A Useful Tool for Natural Product, Mechanistic, and Synthetic Organic Chemistry. *Chem. Rev.* **2012**, *112* (3), 1839–1862. https://doi.org/10.1021/cr200106v. Scaling factors are also available at http://cheshirenmr.info.

(11) Grimblat, N.; Zanardi, M. M.; Sarotti, A. M. Beyond DP4: An Improved Probability for the Stereochemical Assignment of Isomeric Compounds Using Quantum Chemical Calculations of NMR Shifts. *J. Org. Chem.* **2015**, *80* (24), 12526–12534. https://doi.org/10.1021/acs.joc.5b02396.

(12) Moosmann, P.; Ueoka, R.; Grauso, L.; Mangoni, A.; Morinaka, B. I.; Gugger, M.; Piel, J. Cyanobacterial Ent-Sterol-Like Natural Products from a Deviated Ubiquinone Pathway. *Angew. Chemie Int. Ed.* **2017**, *56* (18), 4987–4990. https://doi.org/10.1002/anie.201611617.

(13) Bally, T.; Rablen, P. R. Quantum-Chemical Simulation of 1H NMR Spectra. 2. Comparison of DFT-Based Procedures for Computing Proton–Proton Coupling Constants in Organic Molecules. *J. Org. Chem.* **2011**, *76* (12), 4818–4830. https://doi.org/10.1021/jo200513q.

(14) Grauso, L.; Teta, R.; Esposito, G.; Menna, M.; Mangoni, A. Computational Prediction of Chiroptical Properties in Structure Elucidation of Natural Products. *Nat. Prod. Rep.* **2019**, *36* (7), 1005–1030. https://doi.org/10.1039/C9NP00018F.

(15) Bruhn, T.; Schaumlöffel, A.; Hemberger, Y.; Bringmann, G. SpecDis: Quantifying the Comparison of Calculated and Experimental Electronic Circular Dichroism Spectra. *Chirality* **2013**, *25* (4), 243–249. https://doi.org/10.1002/chir.22138.

(16) Badertscher, M.; Bühlmann, P.; Pretsch, E. *Structure Determination of Organic Compounds*, Fourth edit.; Springer Berlin Heidelberg: Berlin, Heidelberg, 2009. https://doi.org/10.1007/978-3-540-93810-1.

(17) Singldinger, B.; Dunkel, A.; Bahmann, D.; Bahmann, C.; Kadow, D.; Bisping, B.; Hofmann, T. New Taste-Active 3-(O-β- d -Glucosyl)-2-Oxoindole-3-Acetic Acids and Diarylheptanoids in Cimiciato-Infected Hazelnuts. *J. Agric. Food Chem.* **2018**, *66* (18), 4662–4673. https://doi.org/10.1021/acs.jafc.8b01216.

(18) Shen, L.; Sun, D. Total Synthesis and Structural Revision of Engelhardione. *Tetrahedron Lett.* **2011**, *52* (35), 4570–4574. https://doi.org/10.1016/j.tetlet.2011.06.112.

(19) Cacciola, N. A.; Squillaci, G.; D'Apolito, M.; Petillo, O.; Veraldi, F.; La Cara, F.; Peluso, G.; Margarucci, S.; Morana, A. Castanea Sativa Mill. Shells Aqueous Extract Exhibits Anticancer Properties Inducing Cytotoxic and Pro-Apoptotic Effects. *Molecules* **2019**, *24* (18), 3401. https://doi.org/10.3390/molecules24183401.
